# Computational identification of natural CXCR4-binding candidates relevant to endometriosis and ovarian cancer progression

**DOI:** 10.1371/journal.pone.0359833

**Published:** 2026-10-07

**Authors:** Sinan Karageçili, Deniz Inan, Nouman Ali

**Affiliations:** 1 Özel Nev Hospital, Sanliurfa, Türkiye; 2 Department of Molecular Biosciences, Faculty of Physical and Biological Sciences, Rashid Latif Khan University, Lahore, Pakistan; AMET University, INDIA

## Abstract

Endometriosis and ovarian cancer share invasive, inflammatory, and pro-survival mechanisms in which CXCR4 signaling may play an important role, yet phytochemical modulators of this receptor remain insufficiently characterized. This study addressed that gap by integrating receptor reconstruction, ligand-guided docking, pharmacokinetic and toxicity prediction, pharmacophore mapping, density functional theory, replicated membrane-embedded molecular dynamics, and MM/GBSA analysis. A native human CXCR4 model was generated using SWISS-MODEL with 98.2% template coverage and 100% sequence identity, while the co-crystallized IT1t ligand defined the docking cavity. Eighteen phytochemicals were screened, and naringenin achieved the most favorable predicted docking score (−6.361 kcal/mol), exceeding apigenin (−5.927 kcal/mol) and IT1t (−5.155 kcal/mol). Naringenin formed predicted interactions with Tyr116, Arg188, and Tyr255, showed acceptable drug-likeness, solubility, and gastrointestinal absorption, and displayed a wider HOMO–LUMO gap than apigenin. Two independent 100 ns simulations of the CXCR4 complexes with naringenin, apigenin, and IT1t indicated comparatively consistent structural behavior for naringenin, supported by RMSD, SASA, radius of gyration, hydrogen-bond, PCA, FEL, and DCCM analyses. MM/GBSA estimates were also more favorable for naringenin than apigenin across both replicas. Its predicted LD50 was 2000 mg/kg (toxicity class 4), emphasizing that favorable receptor interactions do not establish safety and require experimental toxicological assessment during further preclinical development. Collectively, naringenin emerged as the most coherent in silico prioritized CXCR4-binding candidate. However, the absence of receptor-binding assays, functional validation, and in vivo confirmation remains a major limitation.

## Introduction

Endometriosis is a chronic, estrogen-dependent inflammatory disorder characterized by the presence of endometrium-like tissue outside the uterine cavity and is frequently associated with pelvic pain, infertility, and impaired quality of life. A population-based investigation using magnetic resonance imaging estimated that approximately 11% of women may have previously undiagnosed endometriosis, although prevalence estimates vary substantially with the population and diagnostic method used [1]. Endometriosis is biologically distinct from ovarian cancer and does not generally undergo malignant transformation. Nevertheless, epidemiological evidence indicates that women with ovarian endometrioma have an increased subsequent risk of ovarian malignancy, with the association being particularly relevant to endometrioid and clear-cell histological subtypes [2]. This association, together with the persistence and recurrence of both conditions, supports investigation of molecular pathways that may contribute to cell survival, invasion, inflammatory signaling, and tissue dissemination.

The C-X-C chemokine receptor type 4 (CXCR4) is a seven-transmembrane G protein-coupled receptor activated principally by C-X-C motif chemokine ligand 12 (CXCL12). In endometriosis, CXCR4 expression has been detected in human endometrial and endometriotic tissues, with variation among lesion types. Experimental stimulation of endometriotic epithelial cells with CXCL12 increased AKT phosphorylation and influenced cellular proliferation, migration, and invasion, whereas pharmacological CXCR4 blockade produced context-dependent effects depending on the presence of CXCL12 [3]. In ovarian cancer models, activation of the CXCL12–CXCR4 axis promoted cellular proliferation, migration, invasion, and peritoneal metastatic dissemination [4]. These findings support CXCR4 as a pharmacologically relevant candidate target in both disease settings, although its biological effects remain dependent on cellular and ligand context.

The pharmacological tractability of CXCR4 is supported by the development of chemically distinct antagonists. Plerixafor, also known as AMD3100, has demonstrated clinical utility in combination with granulocyte colony-stimulating factor for hematopoietic stem-cell mobilization, but it is not an established treatment for either endometriosis or ovarian cancer [5]. Structural studies have also resolved human CXCR4 in complex with the small-molecule antagonist IT1t and the cyclic peptide antagonist CVX15, defining experimentally observed antagonist-binding modes within the receptor cavity [6]. The co-crystallized IT1t ligand therefore provides an appropriate structural benchmark for validating docking within the CXCR4 orthosteric pocket.

The phytochemical library investigated in this study comprised 18 structurally diverse natural compounds selected on the basis of experimentally reported activities relevant to the underlying biology of endometriosis or ovarian cancer, including modulation of inflammatory signaling, cellular proliferation, apoptosis, invasion, angiogenesis, and oxidative-stress responses. Apigenin was included because experimental treatment reduced proliferation and induced apoptosis, mitochondrial dysfunction, reactive oxygen species accumulation, and endoplasmic-reticulum stress in human endometriosis cell models [7]. Naringenin similarly suppressed proliferation and induced mitochondria-mediated apoptosis and endoplasmic-reticulum stress in human endometriosis cells [8]. Its biological relevance was further supported by a rat endometriosis model in which naringenin treatment reduced lesion burden and inhibited invasion-associated molecular markers [9]. These experimental observations justify the inclusion of apigenin and naringenin in the screening panel but do not establish that either compound directly antagonizes CXCR4.

Structure-based computational screening provides a means of generating testable hypotheses regarding ligand orientation, receptor contacts, and relative compound prioritization. Molecular-docking algorithms such as AutoDock Vina can efficiently sample and rank predicted ligand poses, but their scoring functions provide approximate estimates rather than experimentally measured binding affinities [10,11]. Consequently, docking scores should be interpreted together with binding-site interactions, pharmacokinetic and toxicity predictions, electronic properties, and receptor dynamics. For a transmembrane receptor such as CXCR4, dynamic evaluation must also preserve the membrane-associated environment of the receptor and assess reproducibility across independent trajectories.

Accordingly, the present study screened 18 phytochemicals against a reconstructed native human CXCR4 model using an IT1t-defined binding pocket and a redocking-validated protocol. Candidate prioritization was based on comparative docking, residue-level interaction patterns, predicted ADMET properties, and toxicity-related liabilities rather than docking score alone. Apigenin and naringenin were subsequently subjected to comparative pharmacophore characterization, density functional theory calculations, independent membrane-embedded molecular dynamics simulations, and binding free-energy analysis. The objective was to determine whether these compounds could be computationally prioritized as candidate CXCR4-binding ligands for subsequent biochemical and experimental evaluation. The analyses were not intended to establish direct CXCR4 antagonism, therapeutic efficacy, or clinical safety.

## Methodology

### Reconstruction and structural validation of native human C-X-C chemokine receptor (CXCR4)

The X-ray crystallographic structure of human C-X-C chemokine receptor type 4 (CXCR4) bound to the small-molecule antagonist IT1t was retrieved from the RCSB Protein Data Bank (https://www.rcsb.org/) in PDB format using accession ID 3OE6 [6,12]. The 3OE6 structure contains an engineered T4 lysozyme fusion inserted in place of the native third intracellular loop of CXCR4. Therefore, the crystallographic construct was not used directly as the receptor model for molecular docking and molecular dynamics simulations. The coordinates of the co-crystallized IT1t ligand were retained separately for subsequent binding-site identification and redocking validation.

A native human CXCR4 model was generated using the template-based modeling option of the SWISS-MODEL server (https://swissmodel.expasy.org/) [13]. Before modeling, the T4 lysozyme sequence was excluded from the crystallographic construct, and the native human CXCR4 amino-acid sequence, including intracellular loop 3, was used as the target sequence. The CXCR4 component of PDB ID 3OE6 was selected as the structural template because it provides experimentally resolved coordinates for the receptor transmembrane domain. Template–target alignment was inspected to verify the correspondence of the seven transmembrane helices and to ensure that non-CXCR4 residues from the crystallographic fusion construct were not incorporated into the reconstructed receptor.

The generated models were evaluated using the structural quality estimates provided by SWISS-MODEL, including the Global Model Quality Estimate and QMEANDisCo score. The model selected for downstream analysis was downloaded in PDB format and inspected to confirm the continuity of the protein backbone, reconstruction of the native intracellular loop, and absence of T4 lysozyme residues. The selected model was subsequently superimposed on the experimentally resolved CXCR4 portion of the 3OE6 structure to examine preservation of the receptor transmembrane architecture.

Stereochemical validation of the reconstructed CXCR4 model was performed using the SAVES server (https://saves.mbi.ucla.edu/). Backbone dihedral-angle distributions were evaluated through Ramachandran plot analysis using PROCHECK, while overall non-bonded atomic interactions were assessed using ERRAT [14,15]. The reconstructed and structurally evaluated native CXCR4 model was then used for receptor preparation, molecular docking, and membrane-embedded molecular dynamics simulations.

### Co-crystallized ligand-guided definition of the CXCR4 binding site

The ligand-binding site of CXCR4 was defined using the experimentally resolved position of the co-crystallized small-molecule antagonist IT1t in PDB structure 3OE6. The original 3OE6–IT1t complex was opened in BIOVIA Discovery Studio Visualizer V25.1.0.24284, and the co-crystallized ligand was selected to identify its spatial location within the receptor-binding cavity [16]. The geometric center of IT1t was determined from its Cartesian coordinates and used as the reference point for positioning the molecular-docking grid.

The resulting grid center was defined at X = 17.6 Å, Y = 28.8 Å, and Z = +73.4 Å. Particular attention was given to retaining the positive sign of the Z coordinate. The position of the grid box was visually inspected in Discovery Studio Visualizer to confirm that it encompassed the complete co-crystallized IT1t ligand and the surrounding CXCR4 binding-site residues. The same binding-site location was subsequently used for redocking IT1t and for docking all screened phytochemicals.

### Selection and retrieval of phytochemical structures

A panel of 18 phytochemicals was compiled based on peer-reviewed experimental reports describing biological activities relevant to endometriosis or ovarian cancer, including modulation of inflammatory signaling, cellular proliferation, apoptosis, migration, invasion, angiogenesis, and oxidative-stress-associated pathways. The compounds, their reported natural sources, PubChem Compound Identifiers, and the experimental literature supporting their inclusion are summarized in S1 Table.

Compound eligibility was assessed against predefined criteria applied before any structural or docking analysis was performed. A compound was considered eligible when peer-reviewed in vitro or in vivo evidence described an effect on at least one of the biological processes listed above in endometriosis, ovarian cancer, or a mechanistically related gynecological or oncological model; when it originated from a dietary or medicinal plant source and was therefore obtainable for subsequent experimental follow-up; when an unambiguous PubChem Compound Identifier and a complete three-dimensional structure were available for retrieval; and when its physicochemical properties fell within the chemical space typically associated with orally administered small molecules, so that the downstream pharmacokinetic and toxicity predictions would remain interpretable. The eligible compounds were then grouped by chemical class, and representatives of flavones, flavanones, flavonols, stilbenes, phenolic acids, terpenoids, and alkaloids were retained so that the final panel sampled structurally distinct scaffolds rather than close analogues of a single class. Compounds were excluded when the supporting evidence originated from unrelated disease contexts, when the reported activity could not be traced to a primary experimental study, when the deposited structure was incomplete or stereochemically ambiguous, or when the compound belonged to a chemical class associated with non-specific assay interference. Because the panel was defined solely by literature-based and structural criteria, and because none of the selected compounds had an established CXCR4-directed mechanism of action, the screen was designed to be hypothesis-generating rather than confirmatory.

The three-dimensional structures of the selected phytochemicals were retrieved from the PubChem database (https://pubchem.ncbi.nlm.nih.gov/) in Structure Data File format using their respective PubChem Compound Identifiers [17,18]. Each downloaded structure was inspected for chemical identity, atom connectivity, bond-order assignment, and valence consistency. The corresponding Simplified Molecular Input Line Entry System strings were recorded for subsequent pharmacokinetic and toxicity predictions. The ligand structures were converted into Protein Data Bank format for molecular docking, while ligand energy minimization and docking-specific preparation were performed as described in the molecular docking subsection.

### Preparation of the reconstructed CXCR4 receptor

The reconstructed and structurally evaluated native human CXCR4 model was used for receptor preparation. The model was processed using PDBFixer V1.12 to inspect the structure for missing heavy atoms, incomplete side chains, non-standard residues, and connectivity-related inconsistencies [19]. Hydrogen atoms were added using PDBFixer, and the protonation states of ionizable residues were assigned to represent a physiological pH of 7.4. Any structural issues identified during this inspection were corrected before energy minimization. The processed receptor was subsequently imported into Swiss-PdbViewer V4.10 and subjected to energy minimization to reduce local steric clashes and geometrical strain [13]. The minimized receptor structure was saved in Protein Data Bank format and used for subsequent molecular docking and membrane-system preparation.

### Molecular docking and interaction studies of phytochemicals with CXCR4

Molecular docking was performed to evaluate the binding affinity and interaction patterns of the selected phytochemicals with CXCR4 using AutoDock Vina as implemented in SAMSON Connect [20,21]. Before docking, ligand geometries were energy minimized using the Universal Force Field to obtain stable conformations and to reduce strain that could bias docking poses. The prepared receptor and ligand files were converted into the required input format, and all atom types and bond assignments were verified to ensure compatibility with the docking engine.

The molecular-docking grid was defined using the experimentally resolved position of IT1t in the 3OE6 structure. The final reconstructed, protonated, and energy-minimized CXCR4 model was structurally aligned with the CXCR4 component of the original 3OE6–IT1t complex in BIOVIA Discovery Studio Visualizer V25.1.0.24284. Following receptor alignment, the crystallographic IT1t coordinates were retained as the spatial reference for defining the ligand-binding region. The grid center was positioned at X = 17.6 Å, Y = 28.8 Å, and Z = 73.4 Å, corresponding to the geometric center of the aligned IT1t ligand. The grid dimensions were set to 20 × 20 × 20.1 Å to encompass the complete crystallographic ligand and the surrounding binding-site residues. The grid placement was visually inspected before docking, and identical grid parameters were applied to IT1t redocking and phytochemical screening. The AutoDock Vina exhaustiveness value was set to 8.

To ensure the reliability and accuracy of the docking protocol, a validation step was performed using the co crystal ligand present in the CXCR4 structure. The co crystal ligand, Isothiourea-1t, was first extracted from the protein structure and then redocked into the defined binding pocket using the same docking parameters applied to all phytochemicals. Following redocking, the predicted IT1t pose was superimposed on its crystallographic conformation, and the heavy-atom RMSD was calculated using DockRMSD (https://zhanggroup.org/DockRMSD/) [22]. The crystallographic and redocked poses were also visually compared to evaluate reproduction of the ligand orientation and binding-site contacts.

Protein ligand interaction profiling was then carried out using Ligplot+ and Discovery Studio Visualizer to generate two dimensional and three-dimensional interaction maps [16,23–26]. These tools were used to identify hydrogen bonds, hydrophobic contacts, and aromatic interactions between CXCR4 and each ligand, which helps explain docking affinity at the residue level. Interaction analysis is important because docking scores alone do not describe binding mechanisms, and visual inspection of residue level contacts improves confidence in selecting lead compounds for further evaluation.

### *In-silico* ADMET analysis on lead

In silico pharmacokinetic and toxicity profiling of the lead compound was performed to evaluate its drug likeness, absorption, distribution, metabolism, excretion, and potential toxicity risks using established web-based tools. ADME properties were predicted using the SwissADME server (http://www.swissadme.ch/), where the SMILES format of the lead compound was used as input [27,28]. The analysis included evaluation of physicochemical parameters, lipophilicity, water solubility, gastrointestinal absorption, blood brain barrier permeability, P glycoprotein interaction, and compliance with drug likeness rules such as Lipinski, Ghose, Veber, Egan, and Muegge filters. SwissADME was used because it provides a comprehensive and reproducible platform for early-stage drug screening by integrating multiple predictive models into a single workflow.

Toxicity assessment was conducted using the ProTox 3.0 server (https://tox.charite.de/protox3/) where the SMILES structure of the compound was submitted to predict acute toxicity, LD50 values, toxicity class, organ specific toxicity, and various molecular initiating events [29]. The platform also provided predictions related to carcinogenicity, mutagenicity, immunotoxicity, and interaction with nuclear receptor signaling pathways. Toxicity profiling is essential in computational drug discovery because it allows early identification of potential safety concerns, thereby reducing the likelihood of failure in later experimental or clinical stages.

### Pharmacophore characterization and Density functional theory (DFT) of lead drug candidate

Pharmacophore characterization of the lead compound was performed using the Pharmit server (https://pharmit.csb.pitt.edu/) to identify the key spatial and chemical features required for interaction with the CXCR4 binding pocket [30,31]. The optimized lead ligand structure was uploaded to the server, and pharmacophore features such as aromatic centers, hydrogen bond donors, hydrogen bond acceptors, and hydrophobic regions were mapped based on the docked conformation. This analysis was included to define the essential interaction pattern of the lead molecule in a reproducible way and to support future virtual screening or lead optimization studies using feature-based filtering.

Quantum chemical analysis of the lead compound was then carried out using Density Functional Theory in Gaussian 09W software to evaluate its electronic properties and chemical reactivity [32]. The molecular geometry was optimized at the B3LYP level of theory with the 6 31G d p basis set to obtain a minimum energy conformation suitable for electronic structure calculations. Frontier molecular orbitals, including the highest occupied molecular orbital and lowest unoccupied molecular orbital, were calculated from the optimized structure, and the HOMO LUMO energy gap was determined as the difference between the orbital energies. Molecular electrostatic potential mapping was also performed to visualize electron density distribution and identify regions susceptible to electrophilic or nucleophilic attack. DFT analysis was used because it provides mechanistic insight into the intrinsic reactivity and stability of the lead compound, which complements docking and simulation by explaining how electronic properties may influence binding behavior.

### Comparative membrane-embedded molecular dynamics simulations and post-simulation analyses

Comparative all-atom molecular dynamics simulations were performed for three receptor–ligand systems: CXCR4 complexed with naringenin, CXCR4 complexed with apigenin, and CXCR4 complexed with the crystallographic reference antagonist IT1t. Naringenin was evaluated as the computationally prioritized lead compound, apigenin was included as a comparative phytochemical, and IT1t was used as the reference ligand. The selected docked pose of each ligand was combined with the reconstructed native CXCR4 model and used as the starting structure for membrane-system construction.

Each CXCR4–ligand complex was oriented relative to the membrane normal using the Positioning of Proteins in Membranes server (PPM) and subsequently submitted to the CHARMM-GUI Membrane Builder for system construction [33,34]. The receptor–ligand complexes were embedded in a symmetric, pre-equilibrated 1-palmitoyl-2-oleoyl-*sn*-glycero-3-phosphocholine bilayer containing 204 POPC molecules. The systems were hydrated using the CHARMM-modified TIP3P water model, with a minimum water thickness of 22.5 Å on both sides of the membrane. Sodium and chloride ions were added to neutralize the net system charge and obtain an ionic concentration of 0.15 M. The resulting systems were placed in rectangular periodic boxes of approximately 90 × 90 × 113 Å and contained approximately 85,300 atoms, with minor differences arising from ligand identity and the number of counterions.

The CHARMM36m force field was applied to CXCR4, while CHARMM36 lipid parameters were used for the POPC bilayer [35,36]. Ligand topology and parameter files for naringenin, apigenin, and IT1t were generated using the CHARMM General Force Field, and the assigned atom types, net charges, bonded parameters, and molecular connectivity were inspected before system assembly [36]. The CHARMM-compatible TIP3P model was used for water to maintain consistency with the selected force-field framework.

Each assembled system was subjected to energy minimization using the input parameters generated by CHARMM-GUI. The minimized systems were then equilibrated using the standard six-step CHARMM-GUI membrane-equilibration protocol. Positional restraints applied to the receptor, ligand, and lipid headgroups were progressively reduced during consecutive constant-volume and constant-pressure equilibration stages. Temperature and pressure were gradually adjusted to the production conditions while maintaining the receptor orientation within the membrane. The complete equilibration procedure was conducted separately for each receptor–ligand system before production simulation.

Production simulations were performed using GROMACS version 2026.1 [37]. Each of the three receptor–ligand systems was simulated for 100 ns in two independent replicas, resulting in six production trajectories and an aggregate production-simulation time of 600 ns. The replicas were initiated using different random velocity seeds to provide independent sampling of the starting systems. Production simulations were conducted under the NPT ensemble at 310 K and 1 bar. Temperature was controlled using the velocity-rescale thermostat, with the protein–ligand complex, membrane, and solvent–ion groups coupled separately [38]. Pressure was controlled using the C-rescale barostat with semi-isotropic coupling, allowing independent adjustment of the membrane-plane and membrane-normal dimensions [39]. Periodic boundary conditions were applied in all three dimensions.

A 2 fs integration time step was used, and covalent bonds involving hydrogen atoms were constrained using the LINCS algorithm [40]. Long-range electrostatic interactions were treated using the particle-mesh Ewald method with a real-space cutoff of 1.2 nm. Lennard–Jones interactions were calculated using a force-switching function between 1.0 and 1.2 nm, consistent with the CHARMM36 simulation settings. Coordinates were recorded every 100 ps for subsequent trajectory analysis.

Binding free-energy calculations were performed independently for both replicas of each receptor–ligand complex using gmx_MMPBSA (Version v1.6.5) following completion of the production simulations [41]. Trajectory frames were extracted from the production trajectories using the same sampling interval for all complexes. A single-trajectory molecular mechanics–generalized Born surface area (MM/GBSA) protocol was employed to estimate the relative binding free energies of naringenin, apigenin, and IT1t. The calculations were performed using the Generalized Born (GB-OBC1) implicit solvent model (igb = 2) with the default internal dielectric constant of 1.0, external dielectric constant of 80.0, and an ionic strength of 0.15 M. The non-polar solvation energy was estimated from the solvent-accessible surface area (SASA) using the default LCPO surface-area model implemented in gmx_MMPBSA. Entropic contributions were not included, as the objective of the analysis was to compare the relative binding affinities of the receptor–ligand complexes under identical computational conditions. Per-residue free-energy decomposition was performed using the same trajectory frames to identify the major CXCR4 residues contributing to ligand binding. Representative receptor–ligand structures presented in the manuscript were extracted from the final simulation frame (100 ns) of each replica for structural visualization and interaction analysis.

All structural, conformational, and energetic analyses were first performed separately for Replica 1 and Replica 2. Replica-specific profiles were retained to evaluate whether the principal trends were reproduced across independent trajectories. Where combined summary values were reported, the mean and standard deviation were calculated from the two replica-level estimates rather than treating individual trajectory frames as statistically independent observations. Because only two replicas were available for each system, the comparisons were interpreted descriptively and were not used to claim definitive statistical convergence.

### Use of generative artificial intelligence

Generative artificial intelligence tools were used solely to assist with language refinement and editorial improvement of the manuscript text. These tools were not used to generate, analyze, or interpret any of the computational data described in this study. All scientific interpretations, analyses, and conclusions were reviewed and verified by the authors, who take full responsibility for the content of the final manuscript.

## Results

### Reconstruction and structural validation of the human CXCR4 model

The X-ray crystal structure of CXCR4 deposited under PDB ID 3OE6 was used as the structural template for receptor reconstruction. The template was classified in the Protein Data Bank as a signaling protein–hydrolase complex and was resolved by X-ray diffraction at a resolution of 3.20 Å. The structure was expressed in *Spodoptera frugiperda* cells and was deposited as a mutated membrane-protein construct containing components derived from *Homo sapiens* and Tequatrovirus T4. The reported crystallographic refinement statistics included depositor and diffraction-component-correction R-free values of 0.306 and 0.318, respectively, and corresponding R-work values of 0.232 and 0.254. The observed depositor R-value was 0.235.

Template-based modeling in SWISS-MODEL produced a reconstructed human CXCR4 structure with 98.2% template coverage and 100% sequence identity across the aligned region. The resulting model contained 306 CXCR4 residues, without the T4 lysozyme fusion partner or the co-crystallized IT1t ligand, as shown in Fig 1A. The model achieved a Global Model Quality Estimate of 0.92 and a QMEANDisCo global score of 0.89 ± 0.05. The high sequence identity, near-complete template coverage, and model-quality estimates supported the suitability of the reconstructed receptor for subsequent structure-based analyses.

**Fig 1:**
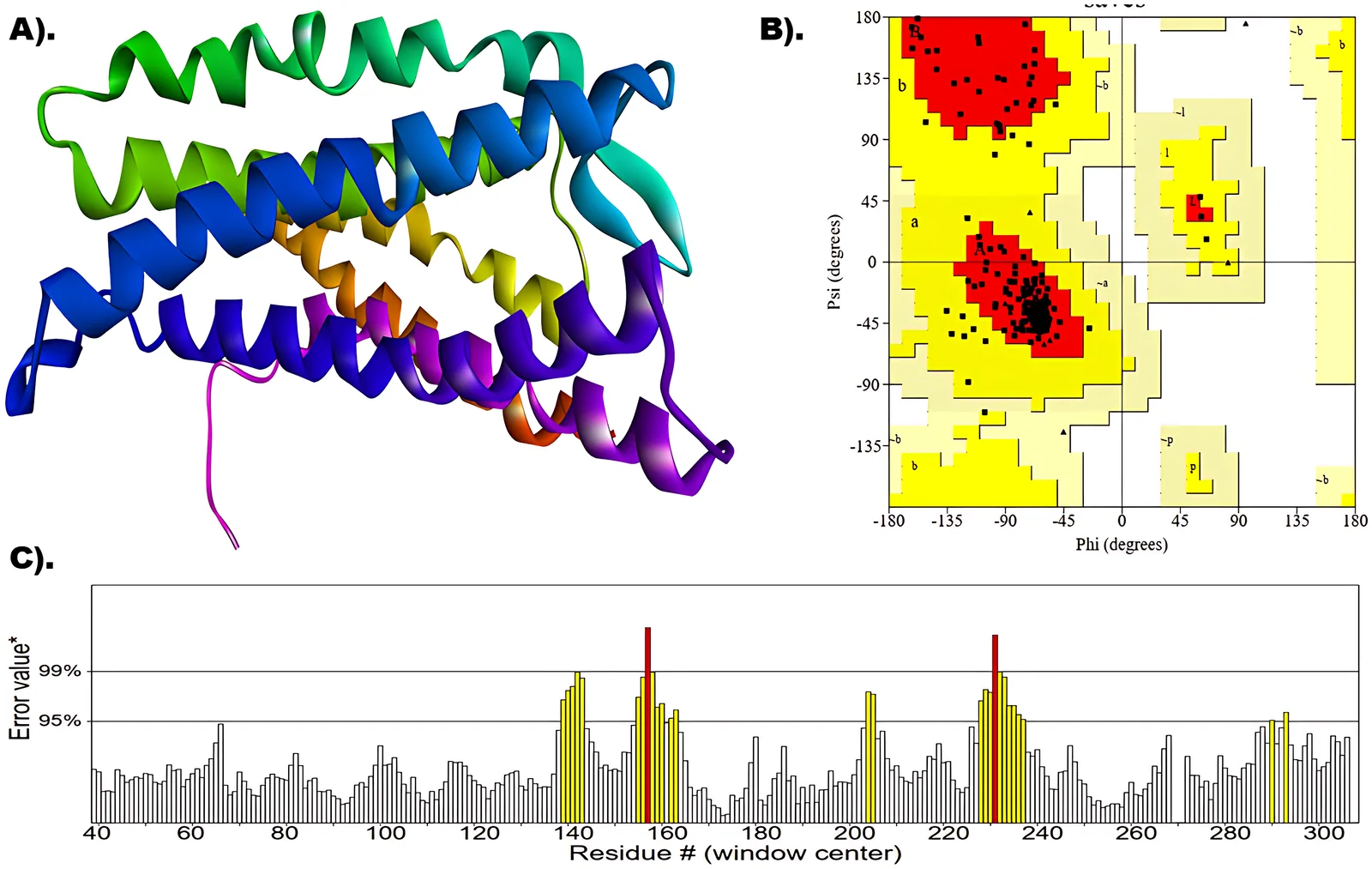
Structural reconstruction and validation of the human CXCR4 receptor model. (A) Three-dimensional structure of the reconstructed CXCR4 model generated by template-based modeling in SWISS-MODEL using PDB ID 3OE6 as the structural template. (B) Ramachandran plot showing the distribution of backbone dihedral angles in the reconstructed model, with 92.9% of the assessed residues in the most favored regions, 7.1% in additionally allowed regions, and no residues in disallowed regions. (C) ERRAT analysis of the reconstructed CXCR4 model, showing an overall quality factor of 89.811 and supporting the favorable quality of the non-bonded atomic interactions in the model.

Stereochemical assessment of the reconstructed model showed that 237 non-glycine and non-proline residues, corresponding to 92.9%, were located within the most favored regions of the Ramachandran plot (Fig 1B). The remaining 18 residues, corresponding to 7.1%, occurred within additionally allowed regions, whereas no residues were identified in generously allowed or disallowed regions. The model also achieved an ERRAT overall quality factor of 89.811, indicating favorable non-bonded atomic interaction patterns (Fig 1C). Collectively, these validation outcomes supported the structural quality of the reconstructed CXCR4 model used for molecular docking and membrane-embedded molecular dynamics simulations.

### Co-crystallized ligand-guided identification of the CXCR4 binding site

The experimentally resolved position of the co-crystallized antagonist IT1t in the 3OE6 structure was used to define the CXCR4 ligand-binding region. Visualization of the crystallographic complex showed that IT1t was positioned within the transmembrane binding cavity of CXCR4, providing an experimentally supported spatial reference for docking-grid placement (Fig 2).

**Fig 2:**
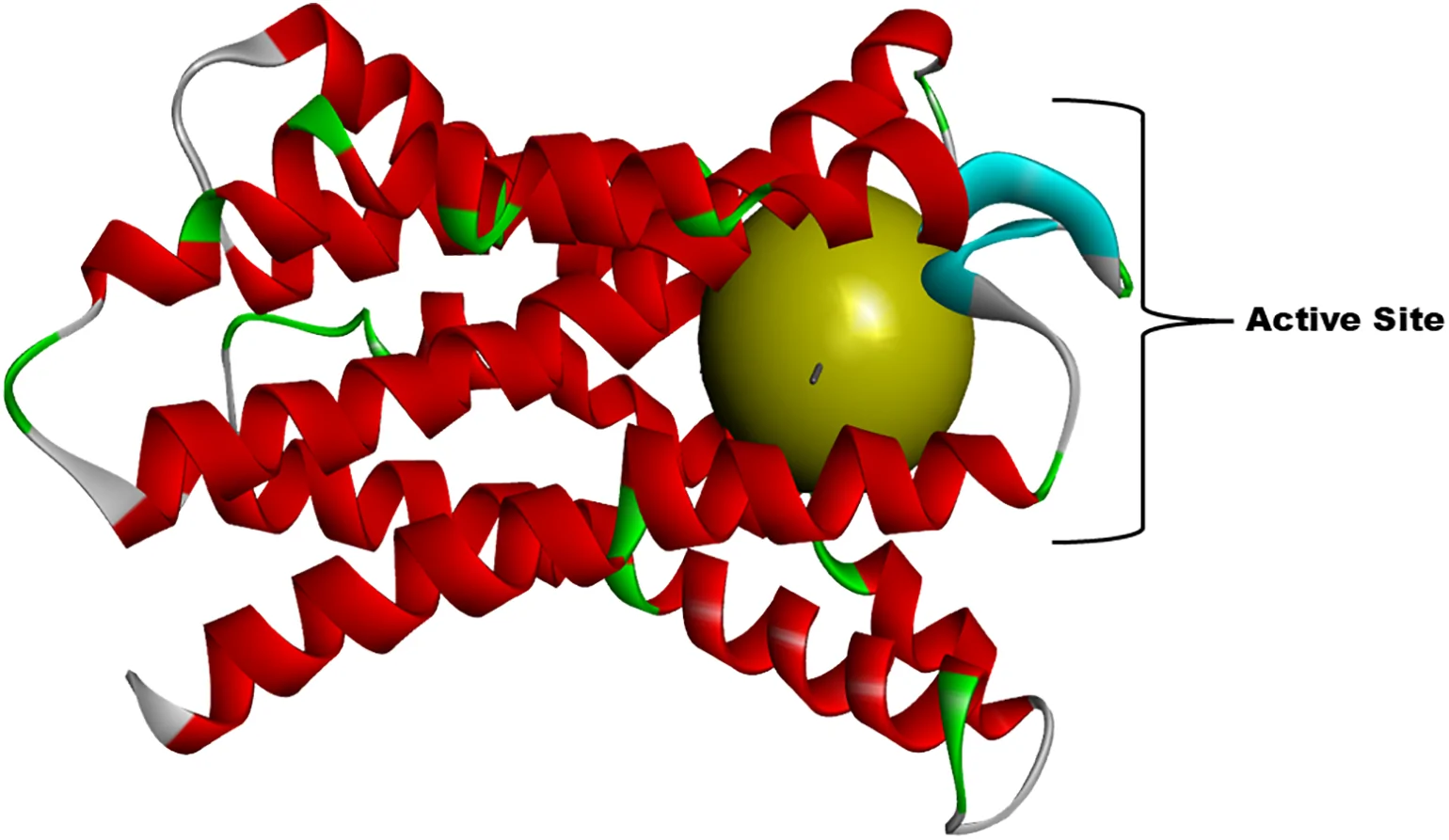
Co-crystallized ligand-guided definition of the CXCR4 binding site. The molecular-docking grid was positioned according to the experimentally resolved location of IT1t after structural alignment of the reconstructed CXCR4 model with the 3OE6–IT1t complex.

Following structural alignment of the reconstructed CXCR4 model with the receptor component of the 3OE6–IT1t complex, the crystallographic ligand position was transferred to the reconstructed receptor coordinate system. The geometric center of the aligned IT1t ligand was located at X = 17.679 Å, Y = 28.848 Å, and Z = 73.411 Å.

The docking grid was centered on this ligand-defined position and visually inspected in Discovery Studio Visualizer. The final grid encompassed the complete IT1t structure and the surrounding CXCR4 binding cavity (Fig 2). This ligand-guided binding-site definition was subsequently applied consistently during IT1t redocking and docking of the complete phytochemical library.

### Molecular docking and comparative interaction analysis

Molecular docking of the 18 phytochemicals against the reconstructed CXCR4 receptor produced predicted docking scores ranging from −3.025 to −6.361 kcal/mol. Naringenin achieved the most favorable predicted docking score of −6.361 kcal/mol, followed by zerumbone at −6.301 kcal/mol, resveratrol at −6.250 kcal/mol, ellagic acid at −6.194 kcal/mol, sulforaphane at −6.169 kcal/mol, and baicalein at −6.075 kcal/mol. By comparison, the co-crystallized reference antagonist IT1t produced a docking score of −5.155 kcal/mol. The complete docking-score ranking of the phytochemical library and reference ligand is presented in Table 1.

**Table 1:** Predicted AutoDock Vina docking scores of the 18 screened phytochemicals and the co-crystallized reference antagonist IT1t against the reconstructed human CXCR4 receptor.

Sr.	Compound	Predicted Binding Affinity (kcal/mol)
1.	Apigenin	−5.927
2.	Baicalein	−6.075
3.	Curcumin	−5.423
4.	Ellagic acid	−6.194
5.	Emodin	−5.668
6.	Genistein	−5.909
7.	Honokiol	−4.113
8.	Luteolin	−5.650
9.	Naringenin	−6.361
10.	Paeonol	−5.917
11.	Parthenolide	−3.843
12.	Quercetin	−5.216
13.	Resveratrol	−6.250
14.	Shikonin	−4.994
15.	Sulforaphane	−6.169
16.	Tanshinone IIA	−3.025
17.	Wogonin	−5.800
18.	Zerumbone	−6.301
19.	Isothiourea-1t	−5.155

Naringenin was therefore selected as the principal computational lead because it ranked first among the screened phytochemicals under the revised docking conditions. Apigenin, which displayed a moderately favorable docking score of −5.927 kcal/mol, was retained as a comparative flavonoid rather than as a co-lead. IT1t was included as the crystallographic reference ligand for docking validation and subsequent comparative analyses. This selection enabled the downstream comparison of the highest-ranking phytochemical, a moderately scoring phytochemical comparator, and an experimentally resolved CXCR4 antagonist.

Interaction profiling showed that apigenin formed two conventional hydrogen bonds with Arg188 and Tyr255, at distances of 2.47 and 2.56 Å, respectively. It also formed a π–π T-shaped interaction with Tyr255 at 5.35 Å. These residue-level contacts are shown in the Discovery Studio interaction map in Fig 3A and the LigPlot+ representation in Fig 3C. The three-dimensional orientation and final docked position of apigenin within the CXCR4 cavity are presented in Fig 4A and Fig 4C, respectively.

**Fig 3:**
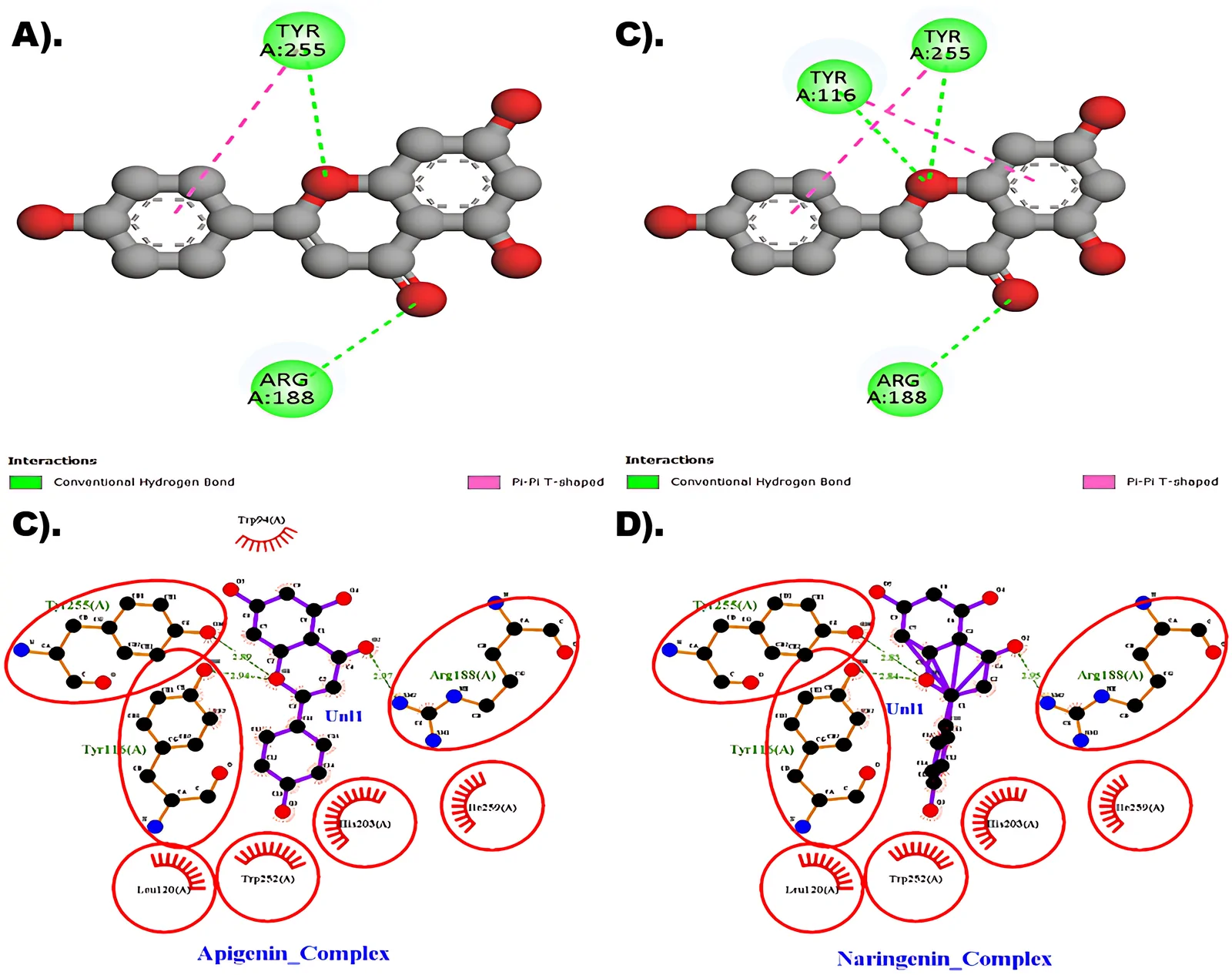
Two-dimensional interaction profiles of apigenin and naringenin within the CXCR4 binding cavity. (A) Discovery Studio interaction map of the CXCR4–apigenin complex. (B) Discovery Studio interaction map of the CXCR4–naringenin complex. (C) LigPlot+ representation of the CXCR4–apigenin interactions. (D) LigPlot+ representation of the CXCR4–naringenin interactions. Hydrogen bonds and non-covalent contacts are displayed according to the respective software conventions.

**Fig 4:**
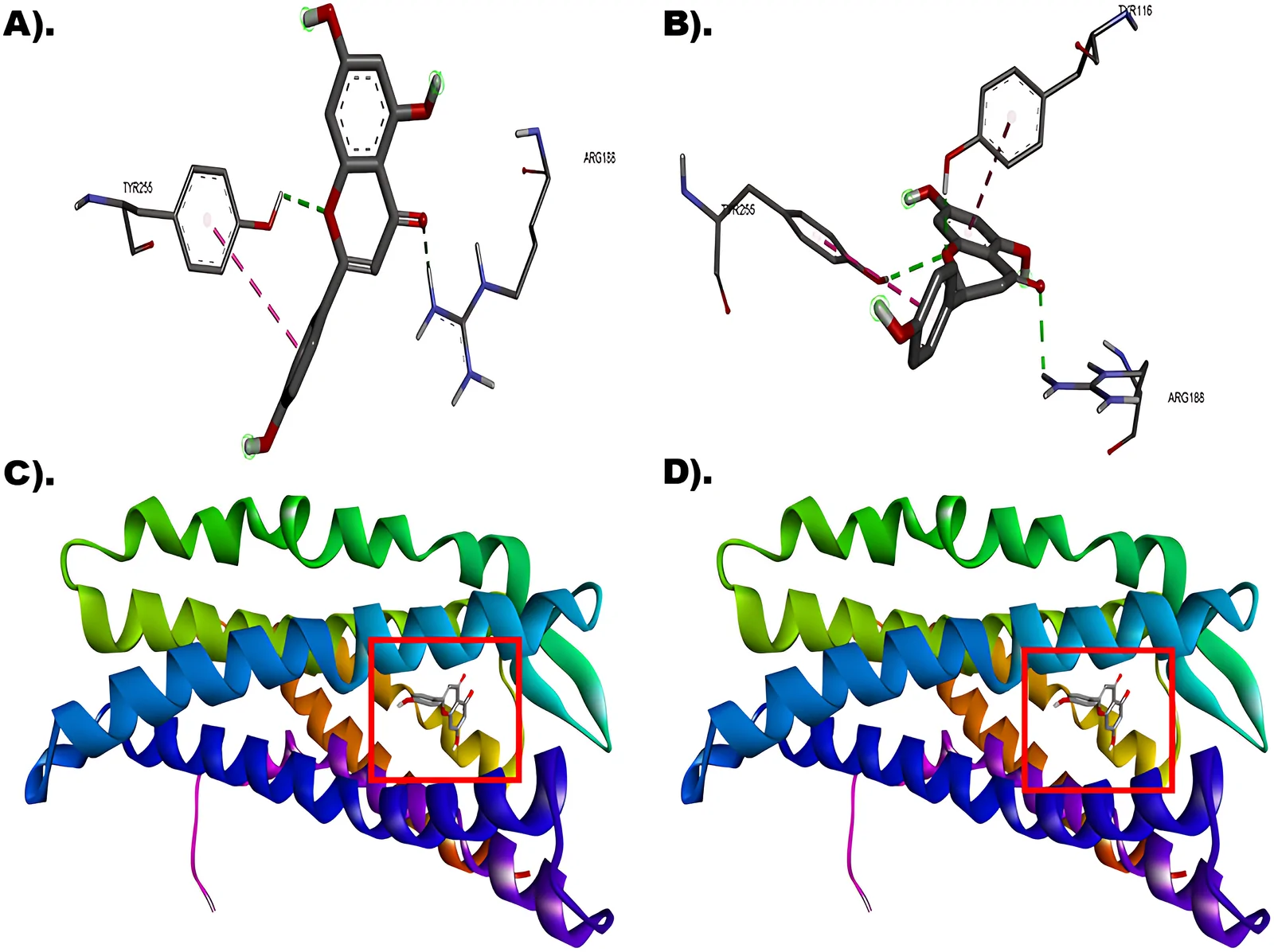
Three-dimensional binding orientations of apigenin and naringenin within CXCR4. (A) Three-dimensional interaction profile of apigenin within the receptor-binding cavity. (B) Three-dimensional interaction profile of naringenin within the receptor-binding cavity. (C) Selected docking pose of apigenin in the reconstructed CXCR4 receptor. (D) Selected docking pose of naringenin in the reconstructed CXCR4 receptor.

Naringenin formed three conventional hydrogen bonds involving Tyr116, Arg188, and Tyr255, with respective interaction distances of 2.22, 2.51, and 2.57 Å. In addition, π–π T-shaped contacts were observed with Tyr116 and Tyr255 at distances of 4.96 and 5.40 Å, respectively. The two-dimensional interaction patterns generated using Discovery Studio and LigPlot+ are shown in Fig 3B and Fig 3D, while the three-dimensional receptor–ligand interaction and docked orientation are presented in Fig 4B and Fig 4D. The complete interaction profiles of apigenin and naringenin are summarized in Table 2.

**Table 2:** Detailed interaction profile of Apigenin and Naringenin with CXCR4, including interacting residues, bond lengths, bond categories, and interaction types.

Residue	Bond Length (Å)	Bond Category	Bond Type
Apigenin with CXCR4			
ARG188	2.47	Hydrogen Bond	Conventional Hydrogen Bond
TYR255	2.56	Hydrogen Bond	Conventional Hydrogen Bond
TYR255	5.35	Hydrophobic	Pi-Pi T-shaped
Naringenin with CXCR4			
TYR116	2.22	Hydrogen Bond	Conventional Hydrogen Bond
ARG188	2.51	Hydrogen Bond	Conventional Hydrogen Bond
TYR255	2.57	Hydrogen Bond	Conventional Hydrogen Bond
TYR116	4.96	Hydrophobic	Pi-Pi T-shaped
TYR255	5.40	Hydrophobic	Pi-Pi T-shaped

Compared with apigenin, naringenin displayed one additional conventional hydrogen bond while retaining interactions with the shared residues Arg188 and Tyr255. Tyr116 was additionally involved in both hydrogen bonding and aromatic interaction with naringenin. These docking observations supported the inclusion of naringenin as the primary lead and apigenin as the moderate comparative compound for the subsequent replicated membrane-embedded molecular dynamics analyses.

The revised docking protocol was evaluated by redocking IT1t into the ligand-defined CXCR4 binding cavity. The experimentally resolved conformation of IT1t is shown in Fig 5A, whereas the comparison of the crystallographic and redocked ligand poses is presented in Fig 5B. Superposition analysis produced an RMSD of 1.364 Å between the crystallographic and predicted poses.

**Fig 5:**
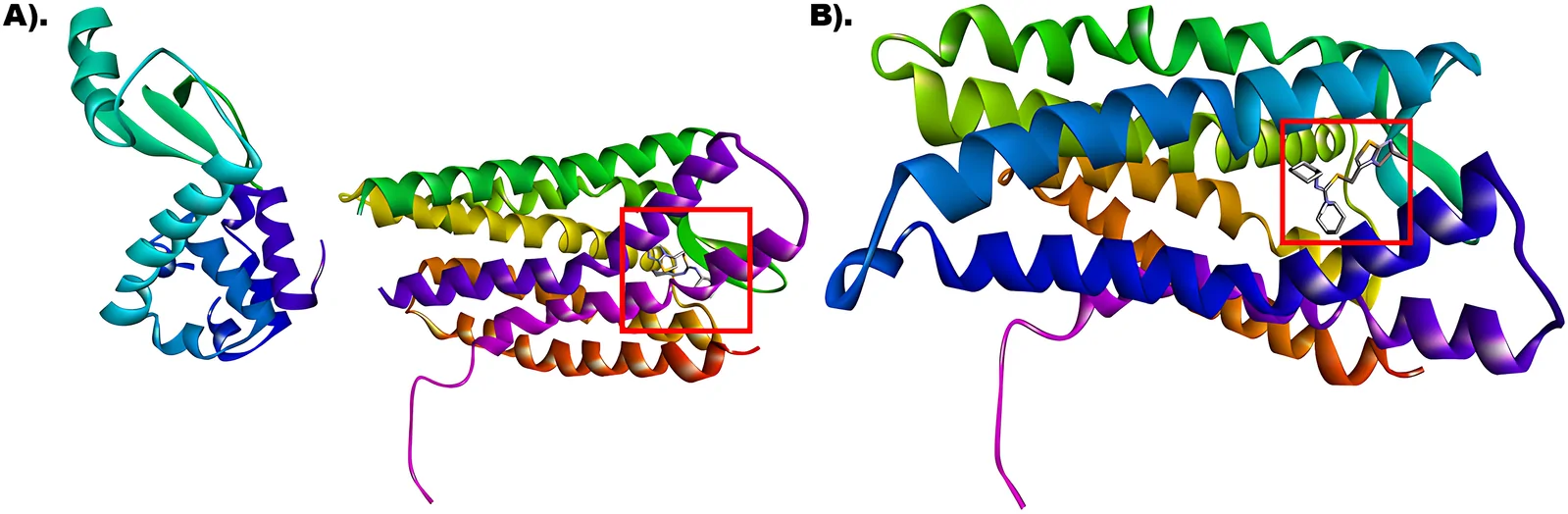
Validation of the revised CXCR4 docking protocol using the co-crystallized antagonist IT1t. (A) Experimentally resolved conformation of IT1t extracted from the 3OE6 crystallographic complex. (B) Structural comparison of the crystallographic and redocked IT1t poses within the ligand-defined CXCR4 binding cavity. The calculated heavy-atom RMSD between the crystallographic and redocked poses was 1.364 Å.

### *In silico* ADME screening and comparative pharmacokinetic profiling

The initial ADME screening showed that all 18 phytochemicals complied with Lipinski’s rule of five and were predicted to exhibit high gastrointestinal absorption. Consensus lipophilicity values ranged from 1.00 for sulforaphane to 4.63 for parthenolide, indicating variation in hydrophobic character across the screened library. Eleven compounds were classified as soluble, six as moderately soluble, and curcumin as poorly soluble. The compounds also differed in their predicted blood–brain barrier permeability and P-glycoprotein substrate status, demonstrating that docking performance alone was insufficient for comparative compound selection. The complete ADME screening outcomes for the 18 phytochemicals are summarized in Table 3.

**Table 3:** Comparative in silico ADME screening of the 18 phytochemicals, including consensus lipophilicity, predicted aqueous-solubility classification, gastrointestinal absorption, blood–brain barrier permeability, P-glycoprotein substrate status, and Lipinski rule-of-five compliance.

Phytochemical	Lipophilicity (Consensus Log *P*_o/w_)	Water Solubility	GI absorption	BBB permeant	P-gp	Lipinski
Apigenin	1.53	Soluble	High	No	No	Yes
Baicalein	2.55	M. Soluble	High	No	Yes	Yes
Curcumin	3.80	P. Soluble	High	Yes	Yes	Yes
Ellagic acid	1.93	Soluble	High	No	No	Yes
Emodin	2.11	Soluble	High	No	No	Yes
Genistein	2.48	Soluble	High	Yes	No	Yes
Honokiol	1.23	Soluble	High	No	No	Yes
Luteolin	1.73	Soluble	High	No	No	Yes
Naringenin	1.87	Soluble	High	No	Yes	Yes
Paeonol	1.73	Soluble	High	No	No	Yes
Parthenolide	4.63	M. Soluble	High	No	Yes	Yes
Quercetin	1.96	Soluble	High	Yes	No	Yes
Resveratrol	2.04	M. Soluble	High	No	No	Yes
Shikonin	1.87	Soluble	High	No	No	Yes
Sulforaphane	1.00	Soluble	High	No	No	Yes
Tanshinone IIA	3.03	M. Soluble	High	No	No	Yes
Wogonin	2.24	M. Soluble	High	No	No	Yes
Zerumbone	2.11	M. Soluble	High	No	No	Yes

Naringenin was retained as the principal computational lead because it combined the most favorable predicted docking score among the screened phytochemicals, −6.361 kcal/mol, with an acceptable predicted ADME profile. Apigenin was not selected as a second lead; rather, it was included as a structurally related, moderately scoring comparative compound. Apigenin produced a docking score of −5.927 kcal/mol, placing it below naringenin while retaining a chemically comparable flavonoid scaffold. This selection provided a more controlled comparison than using an unrelated compound because apigenin and naringenin have closely related molecular sizes, hydrogen-bonding capacities, lipophilicity, solubility, gastrointestinal absorption, and drug-likeness properties. Consequently, differences observed during subsequent molecular dynamics analyses could be interpreted in the context of relatively subtle scaffold and stereochemical differences rather than major differences in molecular size or chemical class.

Apigenin and naringenin had molecular weights of 270.24 and 272.25 g/mol, respectively, and both contained 20 heavy atoms, five hydrogen-bond acceptors, three hydrogen-bond donors, and one rotatable bond. Apigenin contained 16 aromatic heavy atoms and had a fraction Csp^3^ value of 0.00, whereas naringenin contained 12 aromatic heavy atoms and a fraction Csp^3^ value of 0.13. These findings indicate that apigenin possessed a more planar and fully aromatic scaffold, while naringenin contained a slightly greater proportion of three-dimensional, sp^3^-hybridized character. The complete comparative physicochemical profiles are presented in Table 4.

**Table 4:** Comparative physicochemical, lipophilicity, solubility, pharmacokinetic, and drug-likeness profiles of apigenin and naringenin predicted using SwissADME.

ADME parameters	Apigenin	Naringenin
Physicochemical Properties		
Formula	C_15_H_10_O_5_	C_15_H_12_O_5_
Molecular weight	270.24 g/mol	272.25 g/mol
Num. heavy atoms	20	20
Num. arom. heavy atoms	16	12
Fraction Csp3	0.00	0.13
Num. rotatable bonds	1	1
Num. H-bond acceptors	5	5
Num. H-bond donors	3	3
Molar Refractivity	73.99	71.57
TPSA	90.90 Å^2^	86.99 Å^2^
Lipophilicity		
Consensus Log *P*_o/w_	1.53	1.87
Water Solubility		
Log *S* (ESOL)	−3.23	−3.49
Class	Soluble	Soluble
Pharmacokinetics		
GI absorption	High	High
BBB permeant	No	No
P-gp substrate	No	Yes
Log *K*_p_ (skin permeation)	−6.61 cm/s	−6.17 cm/s
Drug-likeness		
Lipinski	Yes; 0 violation	Yes; 0 violation
Ghose	Yes	Yes
Veber	Yes	Yes
Egan	Yes	Yes
Muegge	Yes	Yes
Bioavailability Score	0.55	0.55

Both compounds displayed moderate predicted lipophilicity, with consensus Log P values of 1.53 for apigenin and 1.87 for naringenin. Naringenin was therefore predicted to be slightly more lipophilic than apigenin, although both remained within a comparable range. Their topological polar surface areas were 90.90 Å^2^ for apigenin and 86.99 Å^2^ for naringenin. The slightly lower polar surface area of naringenin was consistent with its marginally higher predicted lipophilicity. Apigenin and naringenin had ESOL Log S values of −3.23 and −3.49, respectively, and both were classified as soluble, as reported in Table 4.

Both compounds were predicted to have high gastrointestinal absorption and were not predicted to cross the blood–brain barrier. Their shared lack of predicted blood–brain barrier permeability suggests limited central nervous system exposure under the applied model, although this prediction requires experimental confirmation. A difference was observed in their predicted P-glycoprotein behavior: apigenin was classified as a non-substrate, whereas naringenin was classified as a P-glycoprotein substrate. This result identifies possible transporter-mediated efflux as a predicted pharmacokinetic consideration for naringenin, but it does not by itself exclude the compound from further investigation.

The predicted skin permeation coefficients were −6.61 cm/s for apigenin and −6.17 cm/s for naringenin, indicating low predicted skin permeation for both compounds. Apigenin and naringenin also satisfied the Lipinski, Ghose, Veber, Egan, and Muegge drug-likeness filters, with no Lipinski violations and identical predicted bioavailability scores of 0.55. Thus, the ADME comparison did not identify a major rule-based drug-likeness disadvantage for either compound. Naringenin remained the primary lead because of its stronger predicted CXCR4 docking performance, whereas apigenin served as a chemically and pharmacokinetically comparable moderate compound for evaluating whether the docking-based ranking was reproduced during dynamic simulations.

The acute oral toxicity profiles of apigenin and naringenin were computationally estimated using ProTox. Apigenin showed a predicted median lethal dose (LD_50_) of 2500 mg/kg and was assigned to toxicity class 5, whereas naringenin showed a predicted LD_50_ of 2000 mg/kg and was assigned to toxicity class 4. Based on these predictions, apigenin exhibited a comparatively lower predicted acute oral toxicity than naringenin, as indicated by its higher LD_50_ value and less severe toxicity classification. The predicted acute toxicity parameters of both compounds are summarized in Table 5.

**Table 5:** Predicted acute oral toxicity profiles of apigenin and naringenin, including prediction and probability.

Category	Target	Apigenin		Naringenin	
		Prediction	Probability	Prediction	Probability
Organ toxicity	Hepatotoxicity	Inactive	0.68	Inactive	0.67
	Neurotoxicity	Inactive	0.86	Inactive	0.84
	Nephrotoxicity	Active	0.60	Active	0.61
	Respiratory toxicity	Active	0.75	Active	0.82
	Cardiotoxicity	Inactive	0.63	Inactive	0.79
Toxicity end points	Carcinogenicity	Inactive	0.62	Inactive	0.62
	Immunotoxicity	Inactive	0.99	Inactive	0.88
	Mutagenicity	Inactive	0.57	Inactive	0.83
	Cytotoxicity	Inactive	0.87	Active	0.59
	BBB-barrier	Inactive	0.51	Active	0.52
	Ecotoxicity	Active	0.51	Active	0.51
	Clinical toxicity	Inactive	0.54	Active	0.50
	Nutritional toxicity	Inactive	0.55	Active	0.52
Tox21-Nuclear receptor signaling pathways	Aryl hydrocarbon Receptor (AhR)	Active	1.0	Inactive	0.54
	Androgen Receptor (AR)	Inactive	0.99	Inactive	0.90
	Androgen Receptor Ligand Binding Domain (AR-LBD)	Inactive	1.0	Inactive	0.98
	Aromatase	Active	0.61	Inactive	0.60
	Estrogen Receptor Alpha (ER)	Active	1.0	Active	0.74
	Estrogen Receptor Ligand Binding Domain (ER-LBD)	Active	1.0	Active	0.66
	Peroxisome Proliferator Activated Receptor Gamma (PPAR-Gamma)	Active	1.0	Active	0.56
	Nuclear factor (erythroid-derived 2)-like 2/ antioxidant responsive element (nrf2/ARE)	Inactive	0.99	Inactive	0.93
	Heat shock factor response element (HSE)	Inactive	0.99	Inactive	0.93
	Mitochondrial Membrane Potential	Active	1.0	Active	0.74
	Phosphoprotein (Tumor Suppressor) p53	Active	1.0	Active	0.69
	ATPase family AAA domain-containing protein 5 (ATAD5)	Active	0.96	Active	0.54
	Thyroid hormone receptor alpha (THRα)	Inactive	0.90	Inactive	0.90
Molecular Initiating	Thyroid hormone receptor beta (THRβ)	Inactive	0.78	Inactive	0.78
	Transthyretin (TTR)	Inactive	0.97	Inactive	0.97
	Ryanodine receptor (RYR)	Inactive	0.98	Inactive	0.98
	GABA receptor (GABAR)	Inactive	0.96	Inactive	0.96
	Glutamate N-methyl-D-aspartate receptor (NMDAR)	Inactive	0.92	Inactive	0.92
	alpha-amino-3-hydroxy-5-methyl-4-isoxazolepropionate receptor (AMPAR)	Inactive	0.97	Inactive	0.97
	Kainate receptor (KAR)	Inactive	0.99	Inactive	0.99
	Acetylcholinesterase (AChE)	Inactive	0.68	Inactive	0.67
	Constitutive androstane receptor (CAR)	Inactive	0.98	Inactive	0.98
	Pregnane X receptor (PXR)	Inactive	0.92	Inactive	0.92
	NADH-quinone oxidoreductase (NADHOX)	Inactive	0.97	Inactive	0.97
	Voltage gated sodium channel (VGSC)	Inactive	0.95	Inactive	0.95
	Na+/I- symporter (NIS)	Inactive	0.98	Inactive	0.98
Metabolism	Cytochrome CYP1A2	Active	1.0	Active	1.0
	Cytochrome CYP2C19	Active	0.99	Active	1.0
	Cytochrome CYP2C9	Active	0.81	Active	0.87
	Cytochrome CYP2D6	Inactive	0.89	Inactive	0.97
	Cytochrome CYP3A4	Active	0.99	Active	0.80
	Cytochrome CYP2E1	Inactive	0.98	Inactive	0.99

The apigenin prediction was generated with an average structural similarity of 81.24% and a prediction accuracy of 70.97%. For naringenin, the corresponding average similarity and prediction accuracy were 76.80% and 69.26%, respectively. Thus, the prediction for apigenin was supported by slightly higher similarity and accuracy estimates than that of naringenin. Nevertheless, the confidence metrics for both compounds remained within a comparable range.

Although naringenin was prioritized as the principal computational lead based on its more favorable predicted docking score and receptor-interaction profile, its lower predicted LD_50_ and toxicity class 4 assignment indicated a comparatively less favorable predicted acute-toxicity profile than apigenin. In contrast, apigenin served as a moderate comparative compound with a relatively favorable predicted acute oral toxicity classification. These findings demonstrate that the stronger predicted CXCR4 binding performance of naringenin did not correspond to a superior predicted acute-toxicity profile. However, the reported LD_50_ values and toxicity classes represent model-based estimates and should not be interpreted as evidence of experimental or clinical safety.

### Comparative pharmacophore feature characterization and relationship with CXCR4 interactions

Comparative pharmacophore mapping revealed related but distinct feature distributions for apigenin and naringenin. Apigenin contained three aromatic features, three hydrogen-bond donor features, four hydrogen-bond acceptor features, and three hydrophobic features, as illustrated in Fig 6A. Naringenin contained two aromatic features, three hydrogen-bond donor features, five hydrogen-bond acceptor features, and two hydrophobic features, as shown in Fig 6B. Thus, apigenin displayed a greater number of aromatic and hydrophobic features, whereas naringenin possessed one additional hydrogen-bond acceptor feature.

**Fig 6:**
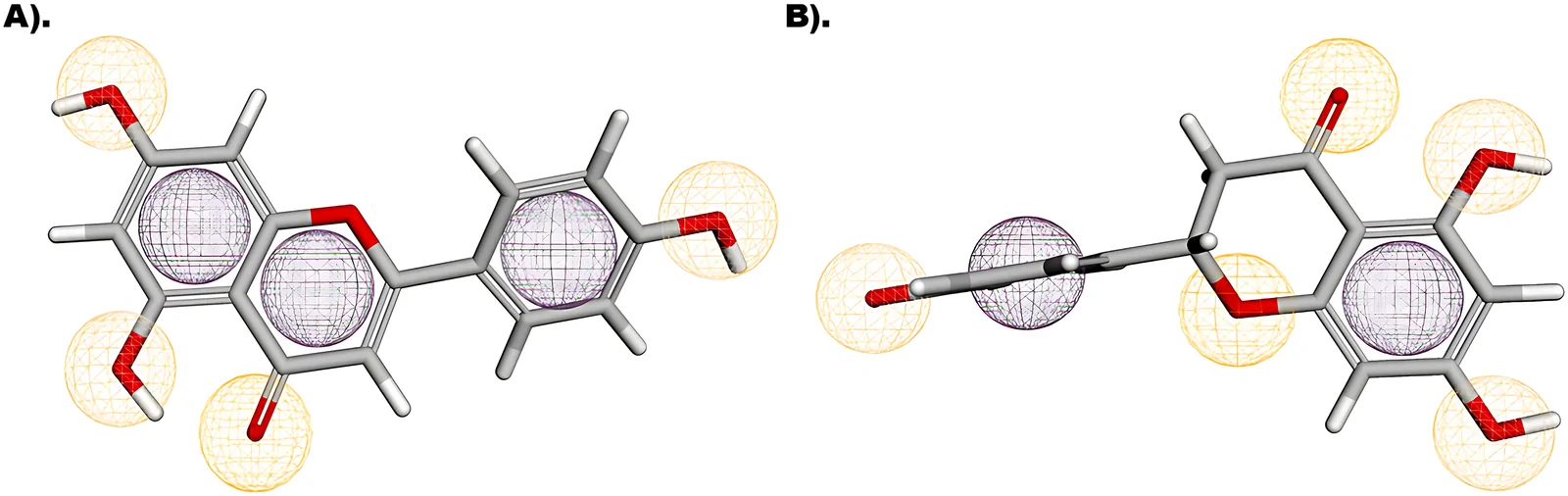
Comparative pharmacophore feature characterization of apigenin and naringenin. (A) Pharmacophore features mapped onto apigenin, comprising three aromatic features, three hydrogen-bond donor features, four hydrogen-bond acceptor features, and three hydrophobic features. (B) Pharmacophore features mapped onto naringenin, comprising two aromatic features, three hydrogen-bond donor features, five hydrogen-bond acceptor features, and two hydrophobic features. Aromatic features are shown in purple, hydrogen-bond acceptors in yellow, hydrogen-bond donors in grey, and hydrophobic features in green.

The pharmacophore characteristics were consistent with the interaction patterns identified from the selected docking poses. Apigenin formed conventional hydrogen bonds with Arg188 and Tyr255, together with a π–π T-shaped interaction involving Tyr255, as reported in Table 2. Its three mapped aromatic features provided potential regions for aromatic recognition, although only the interaction with Tyr255 was observed in the selected docking pose. The hydrogen-bond donor and acceptor features of apigenin similarly supported its capacity to participate in the two predicted polar interactions identified within the CXCR4 binding cavity.

Naringenin formed conventional hydrogen bonds with Tyr116, Arg188, and Tyr255, while π–π T-shaped interactions were observed with Tyr116 and Tyr255, as summarized in Table 2. The greater number of hydrogen-bond acceptor features identified for naringenin was consistent with its broader predicted polar-interaction pattern relative to apigenin. In addition, its two aromatic features corresponded with the aromatic contacts observed with Tyr116 and Tyr255. Therefore, naringenin exhibited a pharmacophore arrangement capable of supporting both polar and aromatic recognition within the CXCR4 binding cavity.

Naringenin showed a more extensive predicted residue-interaction network than apigenin, forming three conventional hydrogen bonds and two aromatic contacts compared with two hydrogen bonds and one aromatic contact for apigenin. This correspondence between the mapped pharmacophore features and the docking-interaction profiles further supported the prioritization of naringenin as the principal computational lead, while apigenin remained a structurally related moderate comparator. Nevertheless, the pharmacophore maps describe the potential interaction features of the ligands and should not be interpreted as independently validated predictive pharmacophore models.

### Comparative electronic-structure analysis of apigenin and naringenin

Density functional theory calculations were used to compare the frontier molecular orbital characteristics, dipole moments, and molecular electrostatic potential distributions of apigenin and naringenin. Apigenin exhibited a calculated HOMO energy of −0.22605 a.u. and a LUMO energy of −0.06293 a.u., producing a HOMO–LUMO energy gap of 0.16312 a.u., equivalent to approximately 4.44 eV. The frontier molecular orbital distributions of apigenin are presented in Fig 7A. Naringenin showed a HOMO energy of −0.22311 a.u. and a LUMO energy of −0.04208 a.u., resulting in a HOMO–LUMO energy gap of 0.18103 a.u., corresponding to approximately 4.93 eV. Its HOMO and LUMO distributions are shown in Fig 7B. The calculated electronic descriptors of both compounds are summarized in Table 6.

**Table 6:** Comparative frontier molecular orbital energies, HOMO–LUMO energy gaps, and dipole moments of apigenin and naringenin calculated through density functional theory.

Compound	HOMO energy (a.u.)	LUMO energy (a.u.)	HOMO–LUMO gap (a.u.)	HOMO–LUMO gap (eV)	Dipole moment (D)
Apigenin	−0.22605	−0.06293	0.16312	4.44	2.8727
Naringenin	−0.22311	−0.04208	0.18103	4.93	2.6979

**Fig 7:**
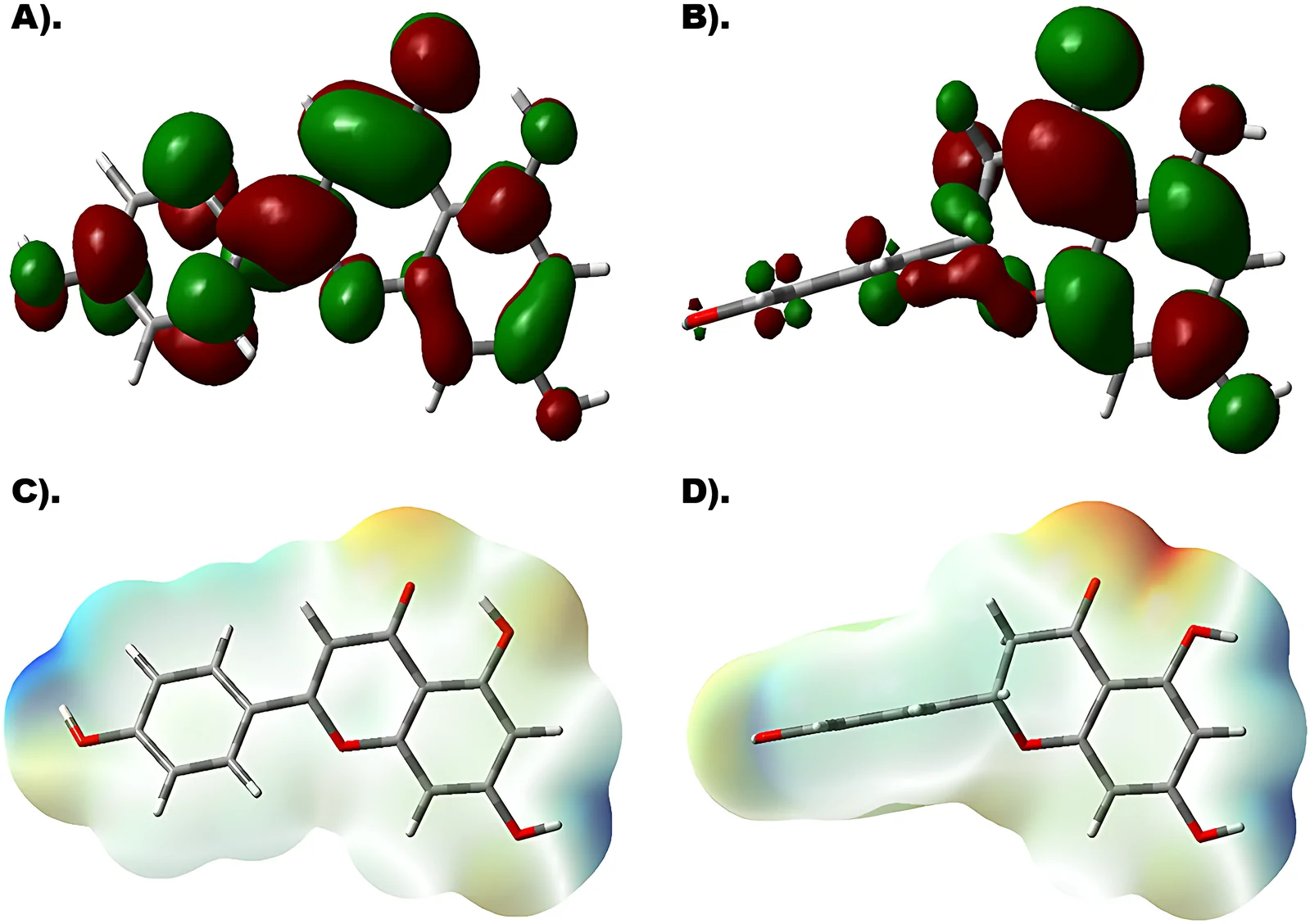
Comparative frontier molecular orbital and molecular electrostatic potential analyses of apigenin and naringenin. (A) HOMO and LUMO distributions of apigenin, with a calculated HOMO–LUMO energy gap of 0.16312 a.u. (4.44 eV). (B) HOMO and LUMO distributions of naringenin, with a calculated HOMO–LUMO energy gap of 0.18103 a.u. (4.93 eV). (C) Molecular electrostatic potential map of apigenin showing the distribution of electron-rich, electron-deficient, and relatively neutral surface regions. (D) Molecular electrostatic potential map of naringenin showing the corresponding electrostatic-potential distribution.

Apigenin displayed a smaller HOMO–LUMO gap than naringenin, with a difference of 0.01791 a.u., or approximately 0.49 eV. The comparatively narrower gap of apigenin suggests a greater predicted tendency toward electronic excitation or charge redistribution, whereas the wider gap of naringenin indicates comparatively greater predicted electronic stability under the applied computational conditions. These electronic descriptors should not be interpreted independently as evidence of stronger CXCR4 binding or biological activity. The calculated dipole moment was 2.8727 D for apigenin and 2.6979 D for naringenin. Apigenin therefore exhibited slightly greater overall molecular polarity, although the relatively small difference indicated broadly comparable charge separation between the two compounds.

The molecular electrostatic potential maps further illustrated the distribution of electron-rich, electron-deficient, and relatively neutral regions across the molecular surfaces of apigenin and naringenin. The electrostatic potential distribution of apigenin is shown in Fig 7C, whereas that of naringenin is presented in Fig 7D. These maps supported the presence of spatially differentiated electrostatic regions that may contribute to hydrogen-bond donor, hydrogen-bond acceptor, and other polar interactions within the CXCR4 binding cavity. However, the MEP distributions represent calculated electronic properties and do not independently establish receptor-binding strength.

### Comparative Molecular dynamics simulation

#### Molecular dynamics behavior of the CXCR4–apigenin complex.

**Protein backbone RMSD:** The two independent 100 ns simulations of the CXCR4–apigenin complex exhibited distinct but bounded protein-backbone RMSD profiles. In Replica 1, the RMSD increased progressively during the first approximately 30 ns, reaching nearly 0.50 nm, and subsequently fluctuated mainly between approximately 0.47 and 0.61 nm. After approximately 70 ns, the trajectory remained relatively consistent around 0.53–0.56 nm until the end of the simulation. In contrast, Replica 2 increased to approximately 0.30 nm within the first 10 ns and thereafter fluctuated predominantly between 0.25 and 0.34 nm, ending near 0.31 nm. The difference between the two profiles indicates replica-dependent conformational sampling, with Replica 2 showing a lower overall structural deviation than Replica 1, as shown in Fig 8A.

**Fig 8:**
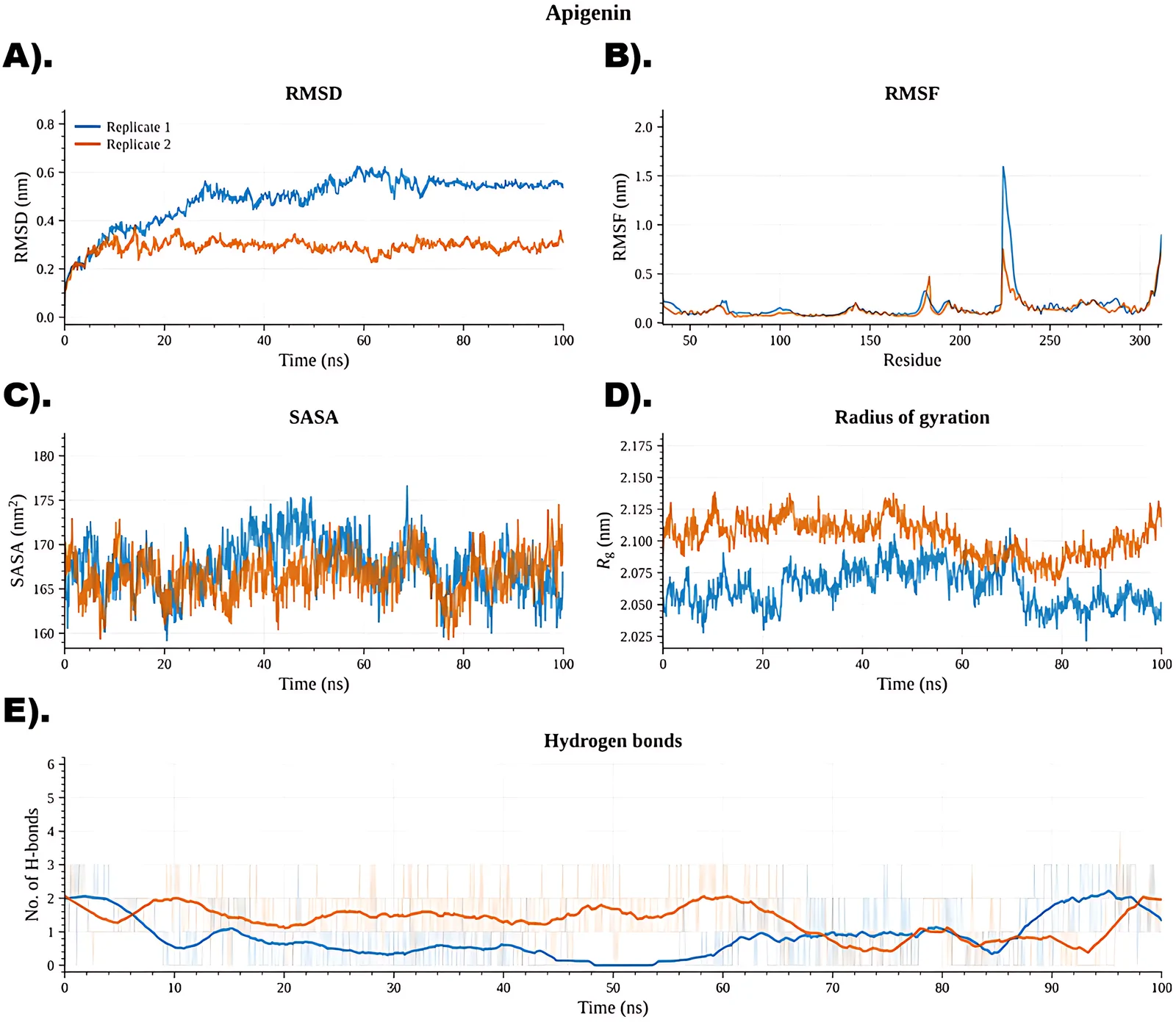
Structural and interaction dynamics of the CXCR4–apigenin complex during two independent 100 ns molecular dynamics simulations. (A) Protein-backbone root-mean-square deviation showing the time-dependent structural deviation of CXCR4. (B) Residue-level root-mean-square fluctuation showing localized receptor flexibility. (C) Solvent-accessible surface area of CXCR4 throughout the trajectories. (D) Radius of gyration reflecting changes in receptor compactness. (E) Number of hydrogen bonds formed between CXCR4 and apigenin during the simulations. Replica 1 and Replica 2 are represented by the blue and orange profiles, respectively.

**Residue-level RMSF:** Residue-level fluctuation analysis showed that most CXCR4 residues maintained RMSF values below approximately 0.20 nm in both replicas. Localized increases were observed around residues 175–190, while the most prominent flexibility occurred around residues 220–230. In this region, Replica 1 reached a maximum fluctuation of approximately 1.60 nm, whereas the corresponding peak in Replica 2 was approximately 0.75 nm. Increased fluctuation was also observed toward the terminal region near residues 305–310, reaching approximately 0.75–0.90 nm. Apart from these localized flexible regions, the RMSF profiles of the two replicas were broadly comparable, as presented in Fig 8B.

**Solvent-accessible surface area:** The solvent-accessible surface area of the CXCR4–apigenin complex fluctuated mainly between approximately 160 and 176 nm^2^ in both replicas. Replica 1 showed a modest increase in surface exposure between approximately 35 and 60 ns, whereas Replica 2 generally remained within a slightly narrower range over the same interval. The profiles overlapped extensively during the latter part of the simulations and showed no sustained increase or decrease in solvent exposure. These observations indicate that the overall solvent-accessible surface of the receptor remained broadly maintained throughout both trajectories, as shown in Fig 8C.

**Radius of gyration:** The radius-of-gyration profiles remained within relatively narrow ranges throughout the simulations. Replica 1 fluctuated mainly between approximately 2.03 and 2.10 nm, whereas Replica 2 generally remained between approximately 2.07 and 2.14 nm. Replica 2 therefore exhibited a marginally higher radius of gyration over most of the trajectory. A moderate decline was observed in both replicas after approximately 60 ns, followed by partial recovery in Replica 2 toward the end of the simulation. Neither replica showed a sustained progressive increase in radius of gyration, suggesting that the receptor did not undergo continuous structural expansion during the 100 ns simulations, as illustrated in Fig 8D.

**Receptor–ligand hydrogen bonds:** Hydrogen-bond analysis revealed dynamic and replica-dependent interactions between apigenin and CXCR4. The instantaneous number of receptor–ligand hydrogen bonds generally ranged from zero to three. In Replica 1, the average hydrogen-bond count decreased from approximately two at the beginning of the simulation to fewer than one during much of the 10–45 ns interval and approached zero between approximately 45 and 55 ns. Hydrogen-bond formation subsequently recovered, reaching approximately two interactions near 90–97 ns before ending at approximately one to two interactions.

Replica 2 maintained a comparatively more persistent hydrogen-bond profile, generally retaining approximately one to two interactions across much of the trajectory. Transient reductions were observed around 70–75 ns and 90–95 ns, followed by recovery to approximately two hydrogen bonds at 100 ns. These findings indicate that apigenin maintained intermittent polar contacts with CXCR4 in both simulations, although the interaction persistence differed between the two independent replicas, as shown in Fig 8E.

The SASA and radius-of-gyration profiles indicated broadly maintained receptor surface exposure and compactness across both simulations. However, the differences in backbone RMSD and receptor–ligand hydrogen bonding demonstrate that the CXCR4–apigenin complex sampled replica-specific conformational and interaction states. Therefore, the results should be interpreted using both trajectories rather than relying on a single simulation.

#### Molecular dynamics behavior of the CXCR4–naringenin complex.

**Protein backbone RMSD:** The CXCR4–naringenin complex displayed comparatively consistent backbone RMSD profiles across the two independent 100 ns simulations. Both replicas began at approximately 0.16–0.18 nm and increased during the initial equilibration period. Replica 1 reached approximately 0.30 nm within the first 15 ns, with a brief maximum of nearly 0.35 nm, and subsequently fluctuated predominantly between 0.27 and 0.34 nm. Replica 2 generally maintained lower RMSD values during the first 75 ns, fluctuating mainly between approximately 0.20 and 0.27 nm, before increasing to approximately 0.28–0.33 nm during the final 20 ns. Both trajectories converged toward similar RMSD values near 0.30 nm at the end of the simulations, indicating broadly comparable final conformational deviation despite minor replica-dependent fluctuations, as shown in Fig 9A.

**Fig 9:**
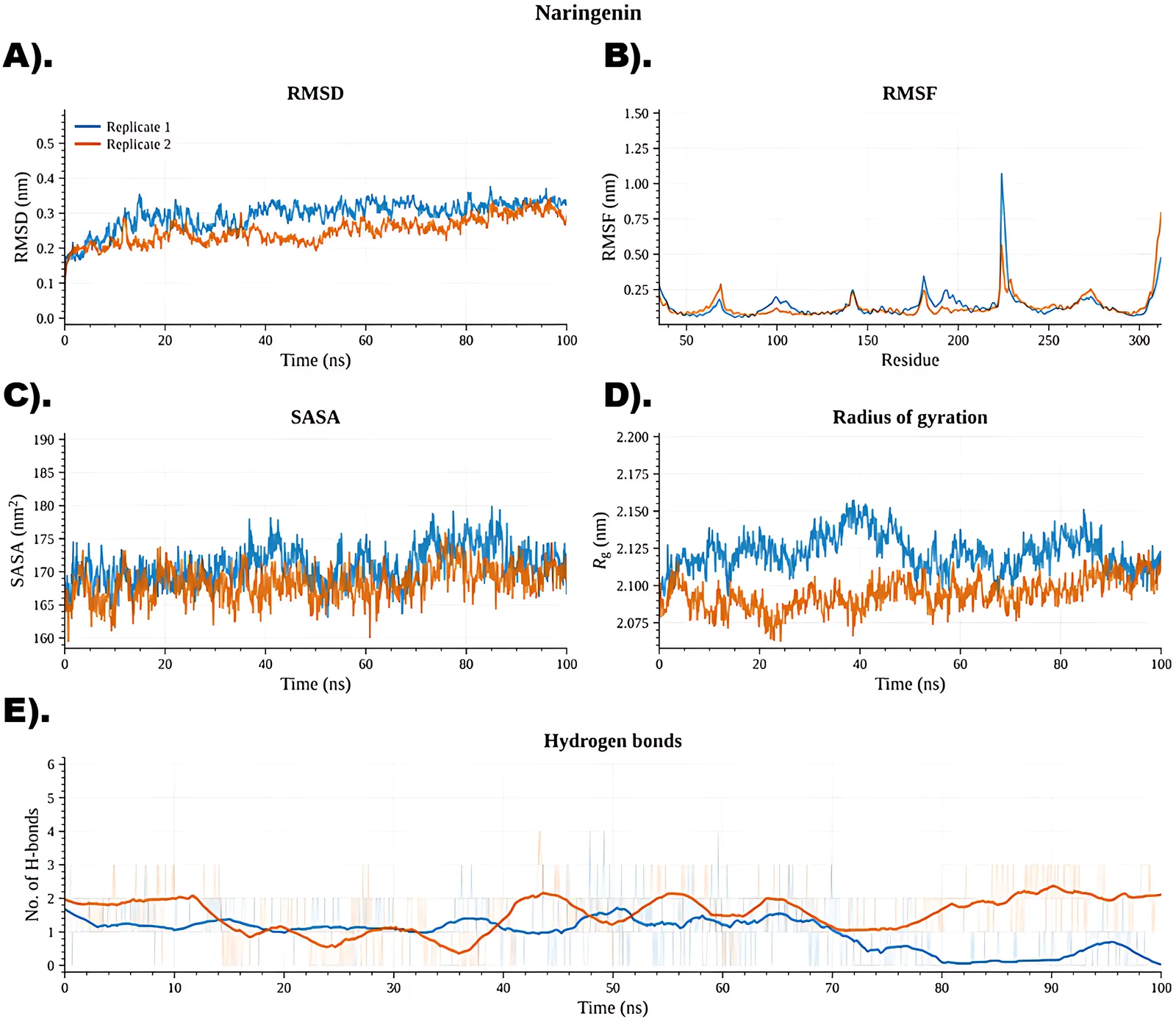
Structural and interaction dynamics of the CXCR4–naringenin complex during two independent 100 ns molecular dynamics simulations. (A) Protein-backbone root-mean-square deviation showing the time-dependent conformational deviation of CXCR4. (B) Residue-level root-mean-square fluctuation showing localized receptor flexibility. (C) Solvent-accessible surface area of CXCR4 throughout the simulations. (D) Radius of gyration reflecting changes in receptor compactness. (E) Number of hydrogen bonds formed between CXCR4 and naringenin during the trajectories. Replica 1 and Replica 2 are represented by the blue and orange profiles, respectively.

**Residue-level RMSF:** The RMSF profiles showed that most CXCR4 residues remained below approximately 0.20 nm in both replicas, indicating limited residue-level fluctuations throughout most of the receptor. Localized increases were observed around residues 60–70, 95–105, 135–145, and 175–205. The most prominent fluctuation occurred near residues 220–230, where Replica 1 reached approximately 1.07 nm and Replica 2 reached approximately 0.56 nm. Increased flexibility was also observed toward the terminal region, with the final residues reaching approximately 0.48 nm in Replica 1 and 0.80 nm in Replica 2. Apart from these localized regions, the two replicas showed broadly comparable fluctuation patterns, as presented in Fig 9B.

**Solvent-accessible surface area:** The solvent-accessible surface area of the CXCR4–naringenin complex remained within a relatively restricted range throughout both trajectories. Replica 1 fluctuated mainly between approximately 166 and 176 nm^2^, with transient increases approaching 179–180 nm^2^ around 40–50 ns and 75–90 ns. Replica 2 generally showed slightly lower values, fluctuating predominantly between approximately 164 and 173 nm^2^. The two profiles overlapped extensively, particularly during the early and final portions of the simulations, and neither replica exhibited a sustained increase or decrease in solvent exposure. These results indicate that the overall solvent-accessible surface of the receptor remained broadly maintained during the simulations, as shown in Fig 9C.

**Radius of gyration:** The radius-of-gyration profiles remained stable within relatively narrow ranges in both replicas. Replica 1 generally fluctuated between approximately 2.10 and 2.15 nm, reaching its highest values near 35–45 ns. Replica 2 showed slightly lower values during most of the simulation, remaining predominantly between approximately 2.07 and 2.11 nm. A gradual increase was observed in Replica 2 during the final part of the trajectory, resulting in similar radius-of-gyration values for both replicas near 100 ns. The absence of a continuous increase in either trajectory indicated that the receptor did not undergo progressive structural expansion during the simulations, as illustrated in Fig 9D.

**Receptor–ligand hydrogen bonds:** Hydrogen-bond analysis revealed dynamic and replica-dependent polar interactions between naringenin and CXCR4. The instantaneous number of receptor–ligand hydrogen bonds generally ranged from zero to four. Replica 1 maintained approximately one to two hydrogen bonds during most of the first 70 ns. However, the interaction count decreased after approximately 70 ns, approaching zero between approximately 80 and 90 ns and ending near zero at 100 ns.

Replica 2 displayed a comparatively more persistent hydrogen-bonding pattern. Approximately two hydrogen bonds were maintained during the first 12 ns, followed by intermittent reductions between approximately 15 and 40 ns. The hydrogen-bond count subsequently recovered to approximately two interactions around 42–45 ns and continued to fluctuate mainly between one and two interactions throughout the middle portion of the trajectory. After approximately 80 ns, Replica 2 maintained nearly two hydrogen bonds and ended with approximately two interactions at 100 ns. These findings indicate that naringenin retained intermittent polar contacts with CXCR4 in both simulations, although the late-stage persistence of these interactions was greater in Replica 2 than in Replica 1, as shown in Fig 9E.

The RMSD, SASA, and radius-of-gyration profiles indicated broadly maintained receptor conformation, solvent exposure, and compactness during the two simulations. The RMSF analysis identified localized flexible regions rather than widespread receptor destabilization. Nevertheless, the differing late-stage hydrogen-bond profiles demonstrate replica-dependent ligand interaction behavior, emphasizing the importance of evaluating both independent trajectories when interpreting the dynamic stability of the CXCR4–naringenin complex.

#### Molecular dynamics behavior of the CXCR4–IT1t complex.

**Protein backbone RMSD:** The CXCR4 complex with the co-crystallized reference antagonist isothiourea-1t (IT1t) displayed replica-dependent conformational adjustment during the initial portion of the two independent 100 ns simulations. In Replica 1, the protein-backbone RMSD increased rapidly from approximately 0.12 nm to 0.35 nm within the first 5 ns and subsequently remained near 0.27–0.32 nm until approximately 32 ns. The RMSD then increased to approximately 0.45–0.50 nm and fluctuated mainly within this range for the remainder of the trajectory, apart from a transient decline near 60 ns. Replica 2 showed larger early-stage fluctuations, increasing to approximately 0.40–0.50 nm between 15 and 35 ns and reaching a maximum of nearly 0.58 nm. After approximately 45–50 ns, both replicas converged toward similar RMSD values of approximately 0.40–0.46 nm and remained broadly comparable until 100 ns, as shown in Fig 10A.

**Fig 10:**
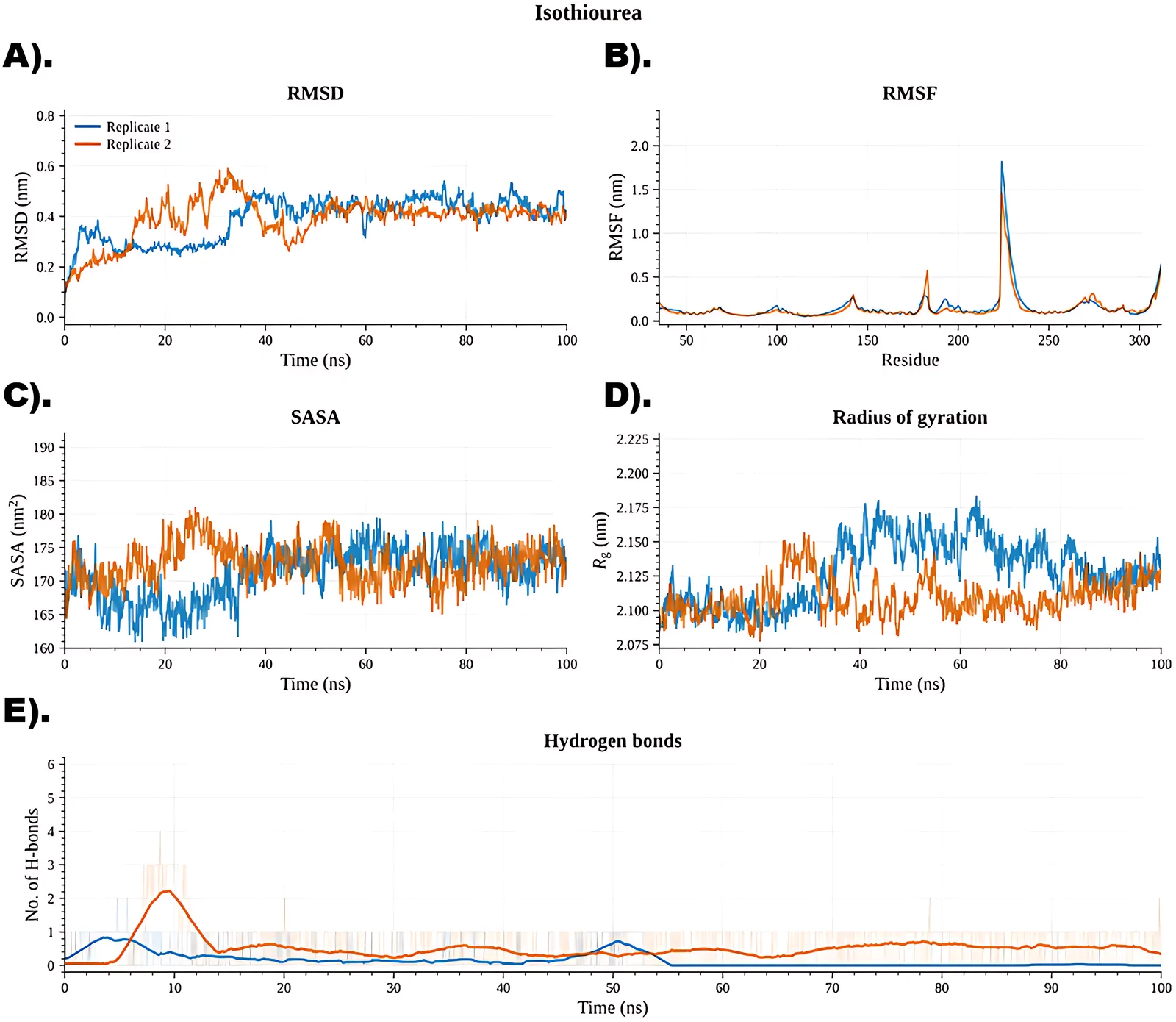
Structural and interaction dynamics of the CXCR4–isothiourea-1t (IT1t) complex during two independent 100 ns molecular dynamics simulations. (A) Protein-backbone root-mean-square deviation showing time-dependent conformational changes in CXCR4. (B) Residue-level root-mean-square fluctuation showing localized receptor flexibility. (C) Solvent-accessible surface area of CXCR4 throughout the trajectories. (D) Radius of gyration reflecting changes in receptor compactness. (E) Number of hydrogen bonds formed between CXCR4 and IT1t during the simulations. Replica 1 and Replica 2 are represented by the blue and orange profiles, respectively.

**Residue-level RMSF:** The RMSF profiles showed that most CXCR4 residues maintained fluctuations below approximately 0.20 nm in both replicas. Localized increases were observed around residues 135–145, 175–190, and 195–205. The most prominent flexible region occurred near residues 220–230, where the RMSF reached approximately 1.8 nm in Replica 1 and 1.5 nm in Replica 2. Increased mobility was also detected near residues 270–280 and toward the C-terminal region, where fluctuations approached approximately 0.6–0.7 nm. Despite differences in peak magnitude, both replicas exhibited similar overall residue-level fluctuation patterns, as presented in Fig 10B.

**Solvent-accessible surface area:** The solvent-accessible surface area of the CXCR4–IT1t complex fluctuated predominantly between approximately 162 and 180 nm^2^. During the first 30 ns, Replica 1 showed comparatively lower SASA values, particularly between approximately 10 and 25 ns, whereas Replica 2 exhibited increased solvent exposure and reached nearly 180 nm^2^ during the same interval. After approximately 35 ns, the two profiles overlapped more extensively and generally fluctuated between approximately 169 and 177 nm^2^. Neither replica displayed a sustained increase or decrease over the complete trajectory, indicating that the overall solvent-accessible surface of the receptor remained broadly maintained, as shown in Fig 10C.

**Radius of gyration:** The radius-of-gyration profiles remained within a relatively narrow range throughout both simulations. Replica 1 generally fluctuated between approximately 2.09 and 2.17 nm and displayed a moderate increase between approximately 35 and 70 ns. Replica 2 remained predominantly between approximately 2.09 and 2.13 nm, although a transient increase to approximately 2.15 nm occurred between 20 and 30 ns. During the final 20 ns, the two replicas approached similar values of approximately 2.11–2.13 nm. The absence of a continuous increase in radius of gyration indicated that CXCR4 did not undergo progressive structural expansion during the IT1t-bound simulations, as illustrated in Fig 10D.

**Receptor–ligand hydrogen bonds:** Hydrogen-bond analysis revealed relatively transient polar interactions between IT1t and CXCR4. In Replica 1, the hydrogen-bond count increased to approximately one interaction during the first 5–10 ns but subsequently declined and remained generally below one throughout most of the trajectory. A brief recovery was observed near 50 ns, after which the hydrogen-bond count approached zero and remained minimal until the end of the simulation.

Replica 2 showed a pronounced early increase, reaching approximately two hydrogen bonds near 9–10 ns. The interaction counts then declined rapidly and fluctuated mainly between approximately zero and one during the remainder of the trajectory. A modest increase to approximately 0.5–0.8 average interactions was observed between approximately 70 and 95 ns before declining near the simulation endpoint. These findings indicate that persistent hydrogen bonding was limited and replica dependent in the CXCR4–IT1t system, as shown in Fig 10E. However, the hydrogen-bond profile alone does not establish loss of ligand binding because other electrostatic, aromatic, and hydrophobic contacts may also contribute to retention of IT1t within the CXCR4 cavity.

The two trajectories showed greater conformational variation during the early simulation period but converged toward broadly comparable backbone RMSD, solvent exposure, and compactness profiles during the later stages. The residue-level analysis identified localized flexible regions, while hydrogen bonding remained comparatively transient. Interpretation of the dynamic stability of the reference complex should therefore incorporate both replicas and additional interaction and binding-energy analyses rather than relying solely on hydrogen-bond counts.

#### Principal component, free-energy landscape, and dynamic cross-correlation analyses of the CXCR4–apigenin complex.

**Replica 1:** The free-energy landscape of Replica 1 was consistent with the PCA distribution and showed multiple low-energy basins separated by higher-energy regions, as presented in Fig 11A. The deepest minimum was located in the negative PC1 and negative PC2 region, corresponding closely to the terminally sampled conformational zone. Additional lower-energy regions were also observed across the positive PC2 space, indicating that the complex accessed more than one energetically favorable state during the simulation. Overall, the FEL of Replica 1 therefore suggests a heterogeneous conformational landscape with several accessible metastable states rather than a single highly restricted free-energy minimum.

**Fig 11:**
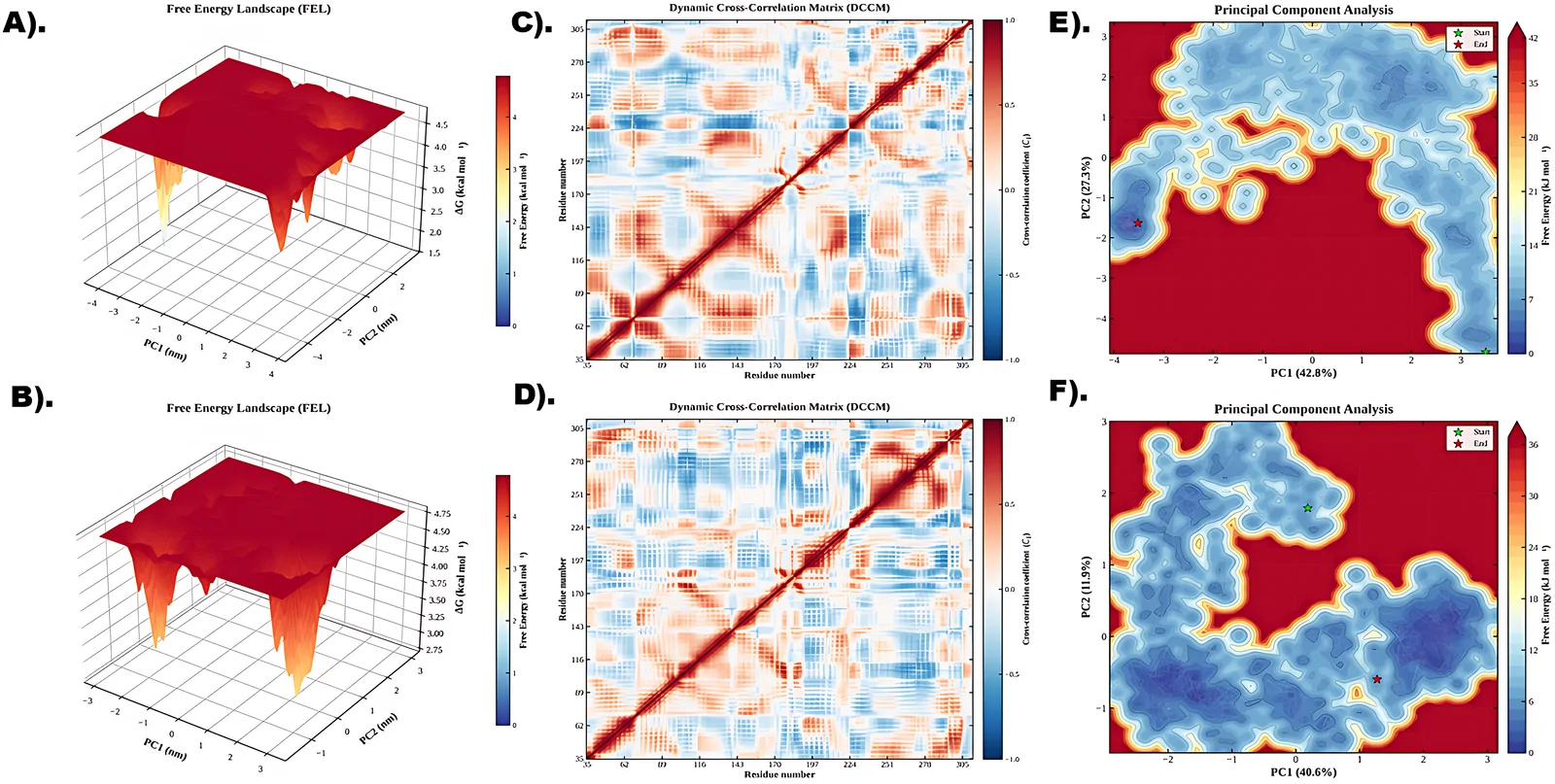
Conformational dynamics of the CXCR4–apigenin complex during two independent 100 ns simulations. (A, B) Free-energy landscapes of Replica 1 and Replica 2, respectively, showing multiple metastable conformational basins. (C, D) Dynamic cross-correlation matrices of Replica 1 and Replica 2, respectively, illustrating correlated and anti-correlated motions among CXCR4 residues. (E) PCA projection of Replica 1, with PC1 and PC2 accounting for 42.8% and 27.3% of the total variance, respectively. (F) PCA projection of Replica 2, with PC1 and PC2 accounting for 40.6% and 11.9% of the total variance, respectively. Green and red stars indicate the initial and final conformations.

The dynamic cross-correlation matrix of Replica 1 demonstrated a mixed pattern of correlated and anti-correlated residue motions, as shown in Fig 11C. Strong positive correlations were observed along the principal diagonal and within several off-diagonal residue blocks, indicating coordinated motions among multiple receptor segments. At the same time, distinct blue regions were present between separated residue ranges, reflecting moderate anti-correlated motions between different parts of the receptor. Collectively, these results indicate that Replica 1 exhibited a balanced internal dynamic network with both cooperative and opposing motions distributed across the CXCR4 structure.

Principal component analysis of Replica 1 showed that the first two eigenvectors accounted for a substantial proportion of the total motion, with PC1 contributing 42.8% and PC2 contributing 27.3%, giving a combined variance of 70.1%. The conformational projection revealed that the CXCR4–apigenin complex sampled a comparatively broad conformational space, with the trajectory extending from the initial region located at positive PC1 and negative PC2 values toward the final region located at negative PC1 and negative PC2 values, as shown in Fig 11E. The presence of multiple populated regions connected by transitional pathways indicates that Replica 1 explored several conformational states rather than remaining confined to a single narrow basin.

**Replica 2:** The corresponding free-energy landscape of Replica 2 also showed multiple minima, with two pronounced low-energy basins located approximately in the negative PC1/negative PC2 region and the positive PC1/negative PC2 region, as shown in Fig 11B. These minima were separated by higher-energy regions, indicating that the trajectory moved between distinct metastable conformations. Compared with Replica 1, the FEL of Replica 2 appeared somewhat more localized into discrete basins, supporting the interpretation that this replica sampled a more compartmentalized conformational space.

The dynamic cross-correlation matrix of Replica 2, presented in Fig 11D, also showed strong positively correlated motions along the diagonal and within several defined residue blocks. However, the anti-correlated regions appeared comparatively less extensive and less intense than those observed in Replica 1. The overall DCCM pattern therefore suggests that Replica 2 retained coordinated internal motions within the receptor while displaying a somewhat less pronounced anti-correlated dynamic network.

For Replica 2, principal component analysis showed that PC1 explained 40.6% of the total variance and PC2 explained 11.9%, corresponding to a combined variance of 52.5%. The conformational projection of Replica 2 is shown in Fig 11F. In comparison with Replica 1, the sampled conformational space appeared more compartmentalized, with two major densely populated regions connected through an intermediate pathway. The trajectory started in the upper central region of the PCA space and ended in the lower right region, indicating a conformational transition during the course of the simulation. The presence of two dominant clusters suggests that Replica 2 sampled a smaller number of major conformational states than Replica 1.

The PCA, FEL, and DCCM analyses indicate that the two apigenin-bound replicas shared the general feature of multi-state conformational sampling but differed in the extent and organization of that sampling. Replica 1 explored a broader conformational space with a higher cumulative contribution from PC1 and PC2 and a more widely distributed free-energy surface, whereas Replica 2 sampled a more compartmentalized landscape with two dominant metastable basins. The DCCM analyses of both replicas showed preserved networks of correlated motions, although Replica 1 exhibited comparatively more evident anti-correlated residue relationships than Replica 2. These findings support the conclusion that the CXCR4–apigenin complex sampled multiple dynamically accessible conformations in both replicas, while still showing replica-dependent variation in internal motion and conformational distribution.

#### Principal component, free-energy landscape, and dynamic cross-correlation analyses of the CXCR4–naringenin complex.

**Replica 1:** The free-energy landscape of Replica 1 contained several local minima distributed across the PC1–PC2 conformational space. A pronounced minimum was observed in the positive PC1 region, while additional shallower minima were distributed across the central and negative PC1 regions. This multi-basin landscape indicates that the CXCR4–naringenin complex sampled several energetically accessible conformational states rather than remaining restricted to a single minimum, as presented in Fig 12A.

**Fig 12:**
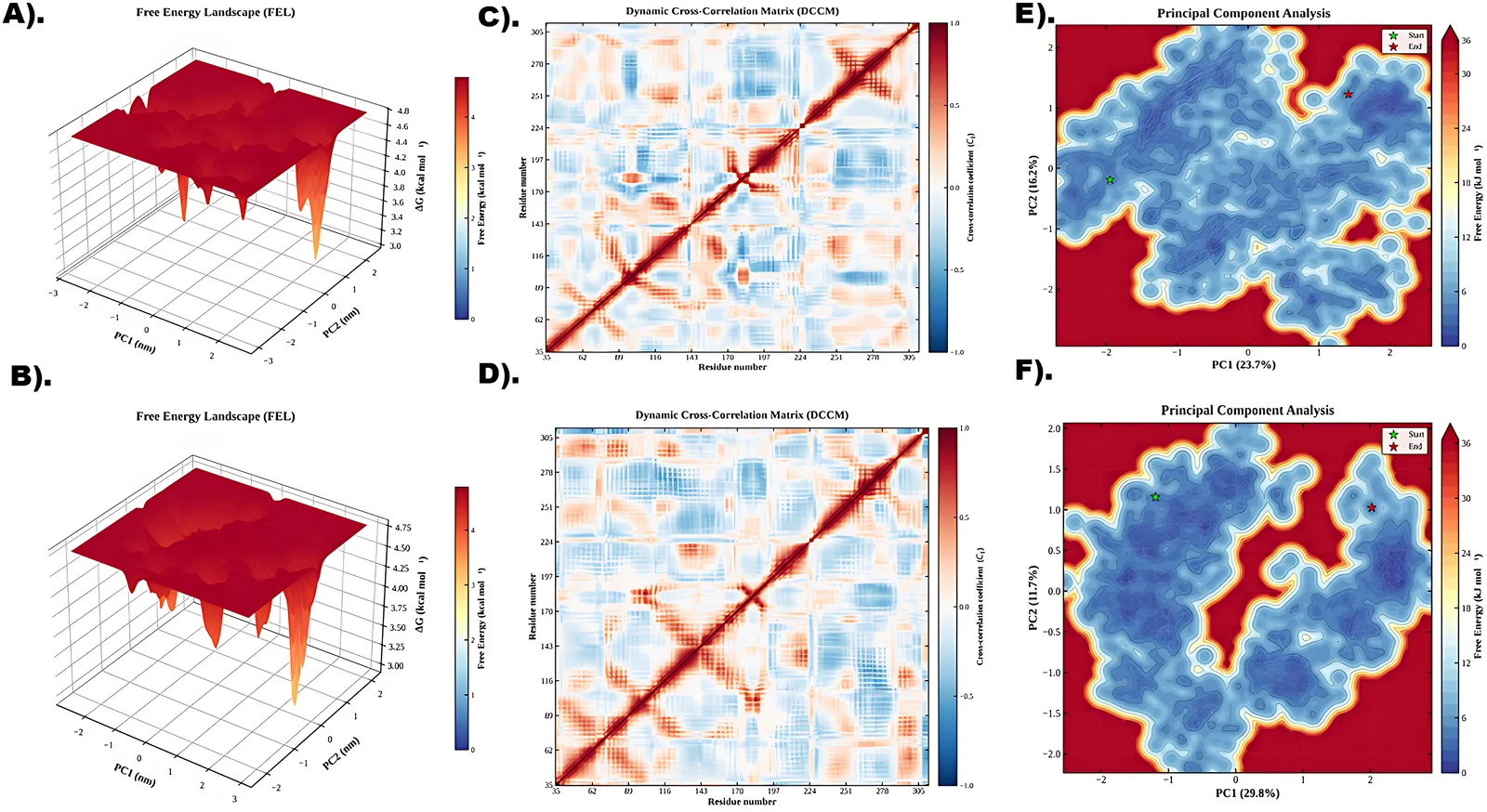
Conformational dynamics of the CXCR4–naringenin complex during two independent 100 ns simulations. (A, B) Free-energy landscapes of Replica 1 and Replica 2, respectively, showing multiple energetically accessible conformational basins. (C, D) Dynamic cross-correlation matrices of Replica 1 and Replica 2, respectively, showing coordinated and opposing residue motions. (E) PCA projection of Replica 1, with PC1 and PC2 accounting for 23.7% and 16.2% of the total variance, respectively. (F) PCA projection of Replica 2, with PC1 and PC2 accounting for 29.8% and 11.7% of the total variance, respectively. Green and red stars indicate the initial and final conformations.

The dynamic cross-correlation matrix of Replica 1 displayed a heterogeneous pattern of positively and negatively correlated residue motions. Strong positive correlations were observed along the principal diagonal and within several defined residue blocks, particularly among spatially or sequentially related receptor regions. Multiple blue off-diagonal regions indicated anti-correlated movements between separated receptor segments, while red off-diagonal blocks represented coordinated motions between distant residues. The coexistence of correlated and anti-correlated regions suggests a distributed internal-motion network within the naringenin-bound receptor, as shown in Fig 12C.

Principal component analysis of Replica 1 showed that PC1 and PC2 accounted for 23.7% and 16.2% of the total conformational variance, respectively, corresponding to a combined contribution of 39.9%. The trajectory sampled an extended conformational region containing several densely populated states. The simulation began near negative PC1 and slightly negative PC2 values and progressed toward a terminal state located at positive PC1 and positive PC2 values, indicating a transition between distinct conformational regions during the 100 ns trajectory. Both the initial and final conformations were located within populated low-energy regions, while the intervening trajectory passed through multiple intermediate states, as shown in Fig 12E.

**Replica 2:** The corresponding free-energy landscape showed multiple energetically accessible basins, including a pronounced minimum in the positive PC1 region and additional minima within the central and negative PC1 portions of the landscape. The presence of several separated depressions indicates that Replica 2 also sampled multiple metastable conformations. However, the conformational distribution appeared to be organized primarily around two broad ensembles, consistent with the clustering observed in the PCA projection in Fig 12B.

The DCCM of Replica 2 revealed strong positive correlations along the diagonal and within several residue blocks, together with extensive anti-correlated regions between spatially separated segments. Coordinated motions were particularly evident within the approximately 224–305 residue region and among several N-terminal and central receptor segments. In contrast, blue off-diagonal regions indicated opposing motions between these regions and other portions of CXCR4. These findings demonstrate that the naringenin-bound receptor retained an organized network of cooperative and opposing residue motions during Replica 2, as presented in Fig 12D.

In Replica 2, PC1 accounted for 29.8% of the total variance, whereas PC2 contributed 11.7%, resulting in a combined contribution of 41.5%. The PCA projection showed two broadly populated conformational regions positioned mainly along the negative and positive PC1 coordinates. The trajectory began in the upper region of the negative-PC1 cluster and ended in the upper region of the positive-PC1 cluster, demonstrating a transition between two major conformational ensembles. Intermediate conformations connected these populated regions, indicating that the receptor explored a continuous but heterogeneous conformational pathway during the simulation, as illustrated in Fig 12F.

Both naringenin-bound replicas sampled multiple conformational states and underwent transitions between their initial and terminal conformational regions. Replica 1 showed a combined PC1–PC2 contribution of 39.9%, whereas Replica 2 showed a slightly higher combined contribution of 41.5%. In both simulations, the free-energy landscapes contained several minima rather than a single isolated basin, indicating conformational heterogeneity within the CXCR4–naringenin complex.

The two DCCM profiles similarly showed coordinated and anti-correlated motions distributed throughout the receptor, although the detailed intensities and residue-block patterns differed between the independent simulations. These replica-dependent variations are consistent with independent conformational sampling and indicate that the dynamic behavior of the CXCR4–naringenin complex should be interpreted from both trajectories rather than from an individual simulation. Overall, the analyses support the presence of multiple energetically accessible receptor conformations without independently establishing ligand potency or experimental binding stability.

#### Principal component, free-energy landscape, and dynamic cross-correlation analyses of the CXCR4–IT1t complex.

**Replica 1:** Principal component analysis of Replica 1 showed that PC1 accounted for 53.0% of the total conformational variance, whereas PC2 contributed 16.2%, yielding a combined contribution of 69.2%. The trajectory sampled two major conformational regions separated primarily along PC1. The simulation began in the positive-PC1 and negative-PC2 region and ended within the larger negative-PC1 conformational ensemble. The movement between these regions indicates a substantial conformational transition during the 100 ns simulation, as shown in Fig 13E.

**Fig 13:**
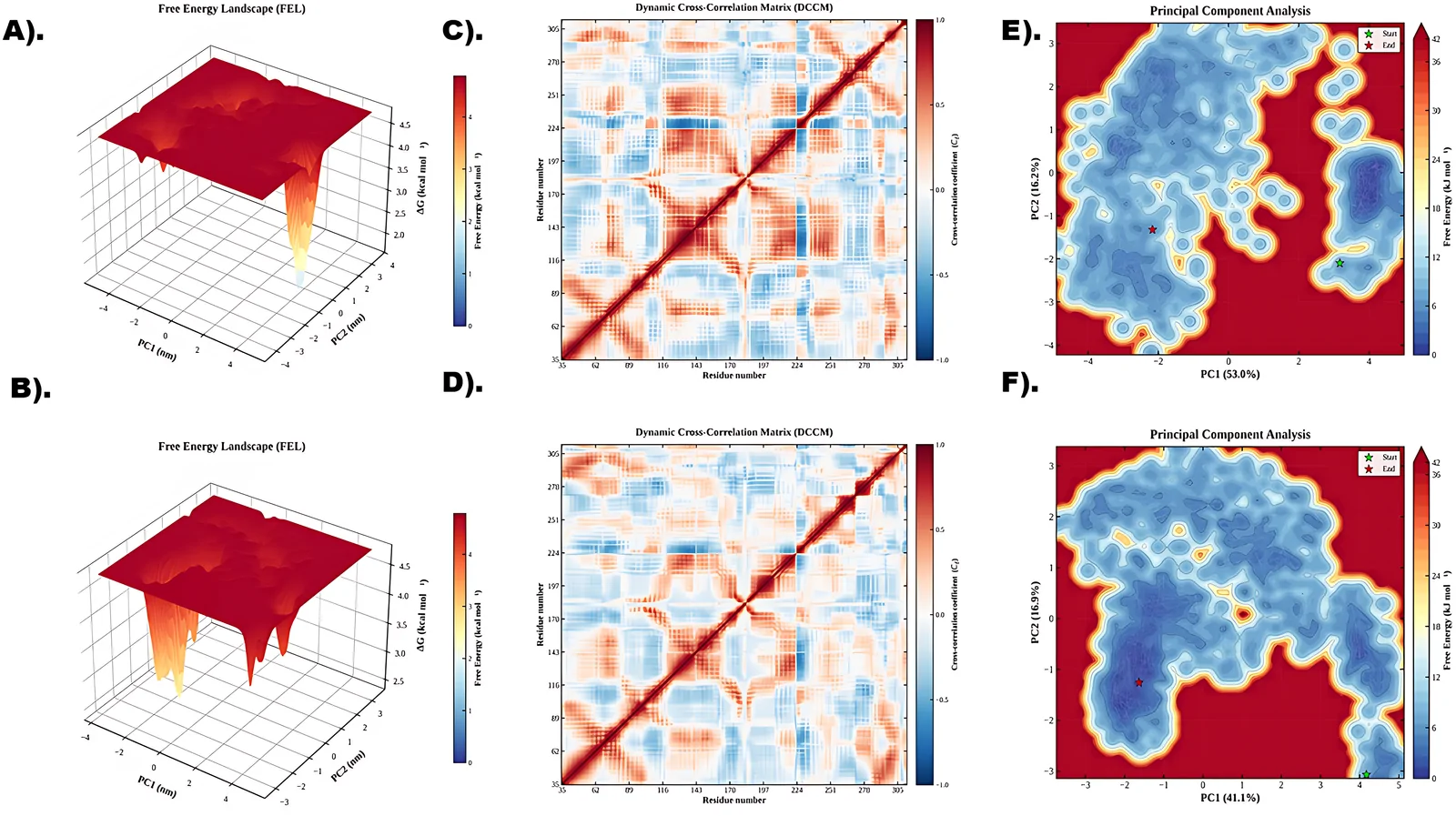
Conformational dynamics of the CXCR4–isothiourea-1t complex during two independent 100 ns simulations. (A, B) Free-energy landscapes of Replica 1 and Replica 2, respectively, showing multiple metastable conformational states. (C, D) Dynamic cross-correlation matrices of Replica 1 and Replica 2, respectively, illustrating correlated and anti-correlated residue motions. (E) PCA projection of Replica 1, with PC1 and PC2 accounting for 53.0% and 16.2% of the total variance, respectively. (F) PCA projection of Replica 2, with PC1 and PC2 accounting for 41.1% and 16.9% of the total variance, respectively. Green and red stars indicate the initial and final conformations.

The corresponding free-energy landscape contained one pronounced low-energy basin within the positive-PC1 region, together with additional shallower minima across the negative-PC1 conformational space. The prominent basin corresponded approximately to the initial conformational region, whereas the final structure was located within a broader set of energetically accessible states at negative PC1 values. This distribution indicates that Replica 1 sampled multiple metastable conformations rather than remaining confined to a single energy minimum, as presented in Fig 13A.

The dynamic cross-correlation matrix of Replica 1 displayed a heterogeneous pattern of correlated and anti-correlated residue motions. Strong positive correlations were evident along the principal diagonal and within several off-diagonal residue blocks, particularly among residues within the approximate 116–170, 190–220, and 250–290 regions. Distinct anti-correlated motions were also observed between separated receptor segments, including regions involving residues around 220–230 and several N- and C-terminal portions of the analyzed receptor. These patterns indicate the presence of coordinated and opposing internal motions within the IT1t-bound CXCR4 receptor, as shown in Fig 13C.

**Replica 2:** For Replica 2, PC1 and PC2 accounted for 41.1% and 16.9% of the total conformational variance, respectively, corresponding to a combined contribution of 58.0%. The trajectory began in a distinct positive-PC1 and strongly negative-PC2 region and progressed toward a terminal state located within the densely populated negative-PC1 and negative-PC2 conformational ensemble. Compared with Replica 1, Replica 2 sampled a more continuous central and negative-PC1 conformational region, although a spatially separated positive-PC1 population was also present, as illustrated in Fig 13F.

The free-energy landscape of Replica 2 showed several energetically accessible minima distributed across the PC1–PC2 space. A comparatively pronounced basin was observed in the negative-PC1 region, while additional minima occurred within the central and positive-PC1 regions. The presence of multiple depressions of varying depth suggests that Replica 2 also sampled several metastable conformational states during the simulation. The terminal conformation was positioned within one of the more densely populated negative-PC1 regions, as shown in Fig 13B.

The DCCM profile of Replica 2 revealed strong positive correlations along the diagonal and within several defined residue blocks. Coordinated motions were particularly evident among central receptor segments and within the C-terminal half of the analyzed sequence. Anti-correlated regions were observed between several spatially separated residue groups, including pronounced opposing motions across the approximate residue-224 boundary and between parts of the 190–220 region and other receptor segments. The combined positive and negative correlations indicate a structured network of cooperative and opposing motions within the CXCR4–IT1t complex, as presented in Fig 13D.

Both IT1t-bound simulations underwent transitions from their initial positive-PC1 regions toward terminal conformations located within negative-PC1 ensembles. Replica 1 showed a higher combined contribution from the first two principal components, 69.2%, than Replica 2, 58.0%, indicating that the dominant collective motions were more strongly concentrated within PC1 and PC2 in Replica 1. Both free-energy landscapes contained multiple minima, although Replica 1 displayed a particularly pronounced basin associated with the positive-PC1 region, while Replica 2 showed a broader distribution of metastable states.

The DCCM profiles of both replicas demonstrated extensive networks of correlated and anti-correlated residue motions, although the detailed strengths and locations of these patterns differed between the independent trajectories. These findings indicate replica-dependent conformational sampling of the CXCR4–IT1t complex and support interpretation based on both simulations rather than either trajectory alone.

#### MM/GBSA binding free-energy and per-residue decomposition analyses of the CXCR4–apigenin complex.

**Replica 1:** The MM/GBSA analysis of Replica 1 produced an estimated total binding free energy of approximately −17.3 kcal/mol, indicating an overall favorable calculated interaction between apigenin and CXCR4 under the applied model. The gas-phase contribution was strongly favorable at approximately −41.1 kcal/mol and was primarily composed of van der Waals and electrostatic contributions of approximately −28.2 and −12.6 kcal/mol, respectively. In contrast, the polar solvation term opposed complex formation, whereas the non-polar surface contribution was modestly favorable. Thus, the favorable gas-phase interactions were partially offset by the calculated solvation penalty, as shown in Fig 14A.

**Fig 14:**
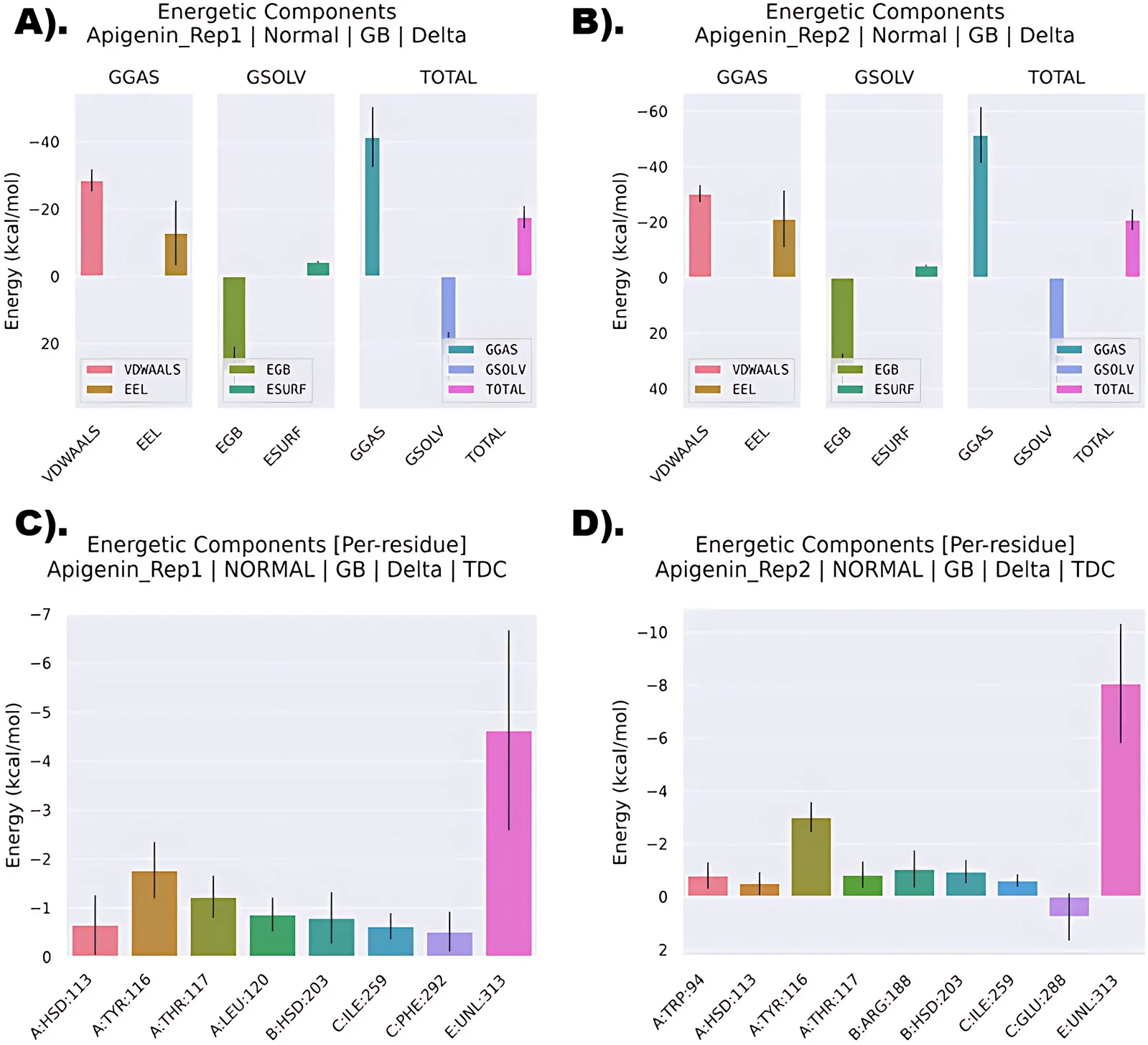
MM/GBSA and amino acid decomposition analyses of the CXCR4–apigenin complex. (A) MM/GBSA Replica 1. (B) MM/GBSA Replica 2. (C) Amino acid decomposition analysis of Replica 1. (D) Amino acid decomposition analysis of Replica 2. Negative values indicate favorable calculated contributions, whereas positive values indicate unfavorable contributions under the applied MM/GBSA model.

Per-residue energy decomposition identified Tyr116 as the strongest favorable receptor contributor in Replica 1, with an estimated contribution of approximately −1.8 kcal/mol. Other favorable contributions were observed for Thr117, Leu120, His203, His113, Ile259, and Phe292, with values ranging from approximately −0.5 to −1.2 kcal/mol. These residue-level contributions are presented in Fig 14C.

**Replica 2:** Replica 2 yielded a slightly more favorable estimated total binding free energy of approximately −20.5 kcal/mol. Its gas-phase contribution was approximately −51.0 kcal/mol, with van der Waals and electrostatic terms contributing approximately −29.9 and −20.7 kcal/mol, respectively. As observed for Replica 1, the favorable gas-phase interactions were opposed by the positive polar-solvation contribution, while the non-polar surface term remained modestly favorable, as shown in Fig 14B.

The per-residue decomposition profile of Replica 2 again identified Tyr116 as the largest favorable receptor contributor, with an estimated energy of approximately −3.0 kcal/mol. Arg188 and His203 each contributed approximately −1.0 kcal/mol, while favorable contributions were also observed for Thr117, Trp94, Ile259, and His113. By contrast, Glu288 displayed a small positive contribution of approximately +0.7 kcal/mol, indicating an unfavorable energetic contribution under the sampled conditions. The major residue-level contributions in Replica 2 are shown in Fig 14D.

Both replicas produced negative estimated total binding free energies, although Replica 2 was approximately 3.2 kcal/mol more favorable than Replica 1. The recurrence of favorable contributions from Tyr116, His113, Thr117, His203, and Ile259 across both independent trajectories indicated partial agreement between the residue-decomposition profiles. Tyr116 was consistently the strongest favorable receptor contributor, whereas Arg188, which participated in the initial docking interaction network, appeared among the major contributors only in Replica 2. These differences indicate replica-dependent rearrangement of the CXCR4–apigenin interaction network during the simulations.

#### MM/GBSA binding free-energy and per-residue decomposition analyses of the CXCR4–naringenin complex.

**Replica 1:** The MM/GBSA analysis of Replica 1 yielded an estimated total binding free energy of approximately −20.0 kcal/mol, indicating a favorable calculated interaction between naringenin and CXCR4. The gas-phase contribution was strongly favorable at approximately −43.8 kcal/mol and was mainly driven by van der Waals and electrostatic terms of approximately −29.5 and −14.3 kcal/mol, respectively. In contrast, the solvation contribution opposed binding overall, with a positive polar-solvation term partially offset by a modestly favorable non-polar surface contribution, as shown in Fig 15A.

**Fig 15:**
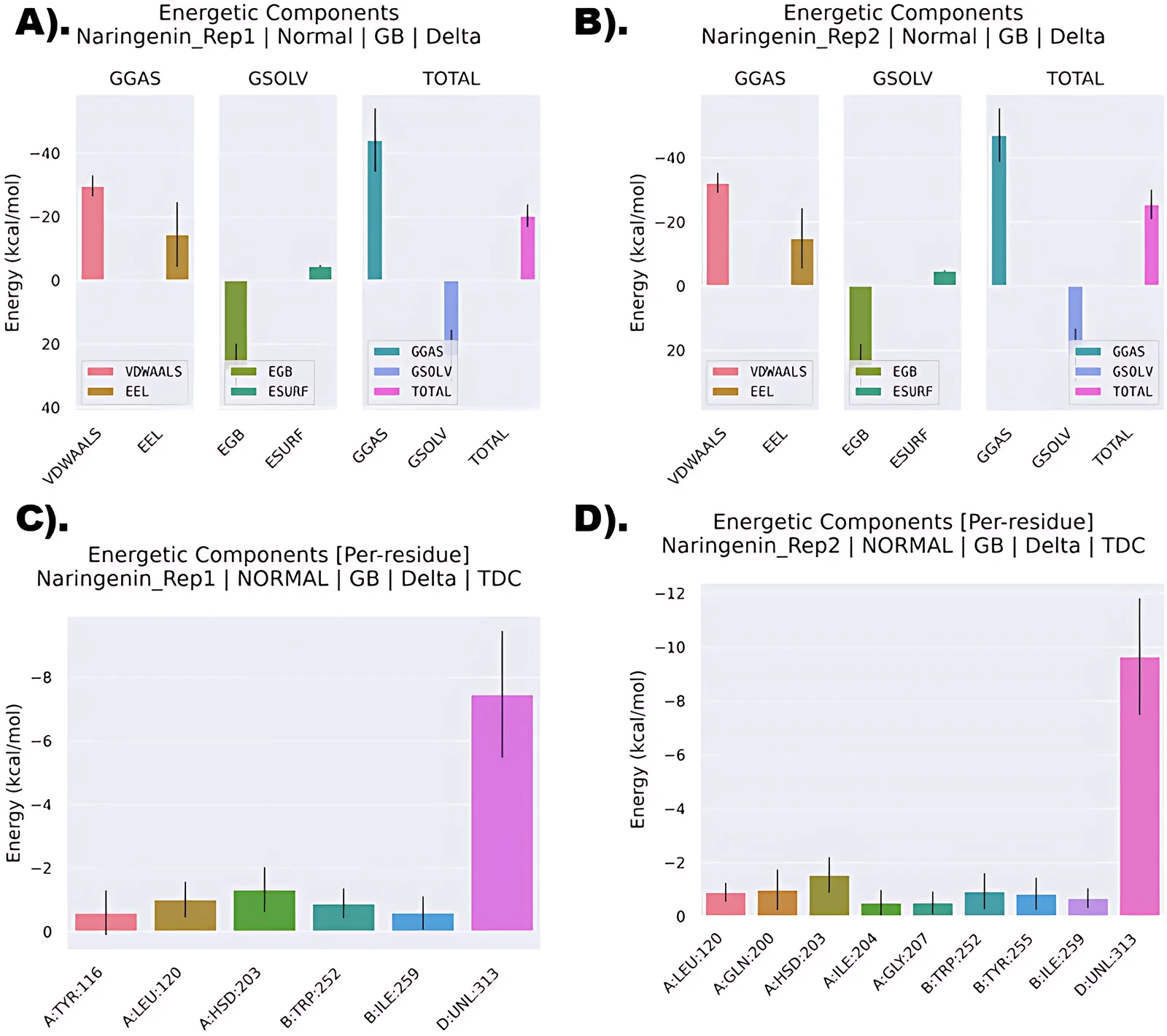
MM/GBSA and amino acid decomposition analyses of the CXCR4–naringenin complex. (A) MM/GBSA Replica 1. (B) MM/GBSA Replica 2. (C) Amino acid decomposition analysis of Replica 1. (D) Amino acid decomposition analysis of Replica 2. Negative values indicate favorable calculated contributions, whereas positive values indicate unfavorable contributions under the applied MM/GBSA model.

Per-residue decomposition of Replica 1 identified His203 as the strongest favorable receptor contributor, with an estimated contribution of approximately −1.3 kcal/mol. Additional favorable contributions were observed for Leu120, Trp252, Tyr116, and Ile259, with values generally ranging from approximately −0.6 to −1.0 kcal/mol, as presented in Fig 15C. These results indicate that the naringenin-bound interaction network in Replica 1 was supported by both hydrophobic and polar residue contributions.

**Replica 2:** For Replica 2, the MM/GBSA calculation produced a more favorable estimated total binding free energy of approximately −25.0 kcal/mol, suggesting stronger calculated binding than that observed in Replica 1. The gas-phase term was approximately −46.5 kcal/mol, with van der Waals and electrostatic contributions of approximately −31.5 and −14.8 kcal/mol, respectively. As in Replica 1, the favorable gas-phase interactions were partially opposed by the solvation penalty, although the overall balance remained favorable, as shown in Fig 15B.

The per-residue decomposition profile of Replica 2 again highlighted His203 as a major favorable contributor, with an estimated value of approximately −1.5 kcal/mol. Other notable favorable contributions were provided by Gln200, Leu120, Trp252, Tyr255, and Ile259, while smaller favorable contributions were also observed for Ile204 and Gly207, as shown in Fig 15D. The recurrence of these residues suggests that naringenin maintained a partially conserved interaction pattern across the two independent trajectories.

Both naringenin-bound replicas produced negative estimated total binding free energies, although Replica 2 was approximately 5 kcal/mol more favorable than Replica 1. Across both trajectories, His203, Leu120, Trp252, and Ile259 appeared repeatedly among the major favorable receptor contributors, whereas Tyr116 was prominent in Replica 1 and Gln200 and Tyr255 were more evident in Replica 2. These findings indicate that the CXCR4–naringenin complex preserved a core set of favorable residue contacts while still showing replica-dependent variation in its detailed energetic pattern.

When compared with the apigenin complex, the naringenin complex showed a more favorable estimated total binding free energy in both replicas, supporting the prioritization of naringenin as the principal computational lead. However, these values represent model-based estimates and should be interpreted as comparative in silico binding-energy trends rather than experimental binding affinities.

#### MM/GBSA binding free-energy and amino acid decomposition analyses of the CXCR4–isothiourea-1t complex.

**Replica 1:** The MM/GBSA analysis of the CXCR4–isothiourea-1t complex in Replica 1 yielded an estimated total binding free energy of approximately −7 kcal/mol, indicating a comparatively weaker but still favorable calculated interaction under the applied model. The gas-phase interaction energy remained favorable at approximately −29 kcal/mol and was dominated by a strong van der Waals contribution of about −34 kcal/mol, whereas the electrostatic term was slightly unfavorable at approximately +5 kcal/mol. The solvation term opposed binding overall, with a positive polar-solvation contribution partially offset by a modestly favorable non-polar surface term, as shown in Fig 16A.

**Fig 16:**
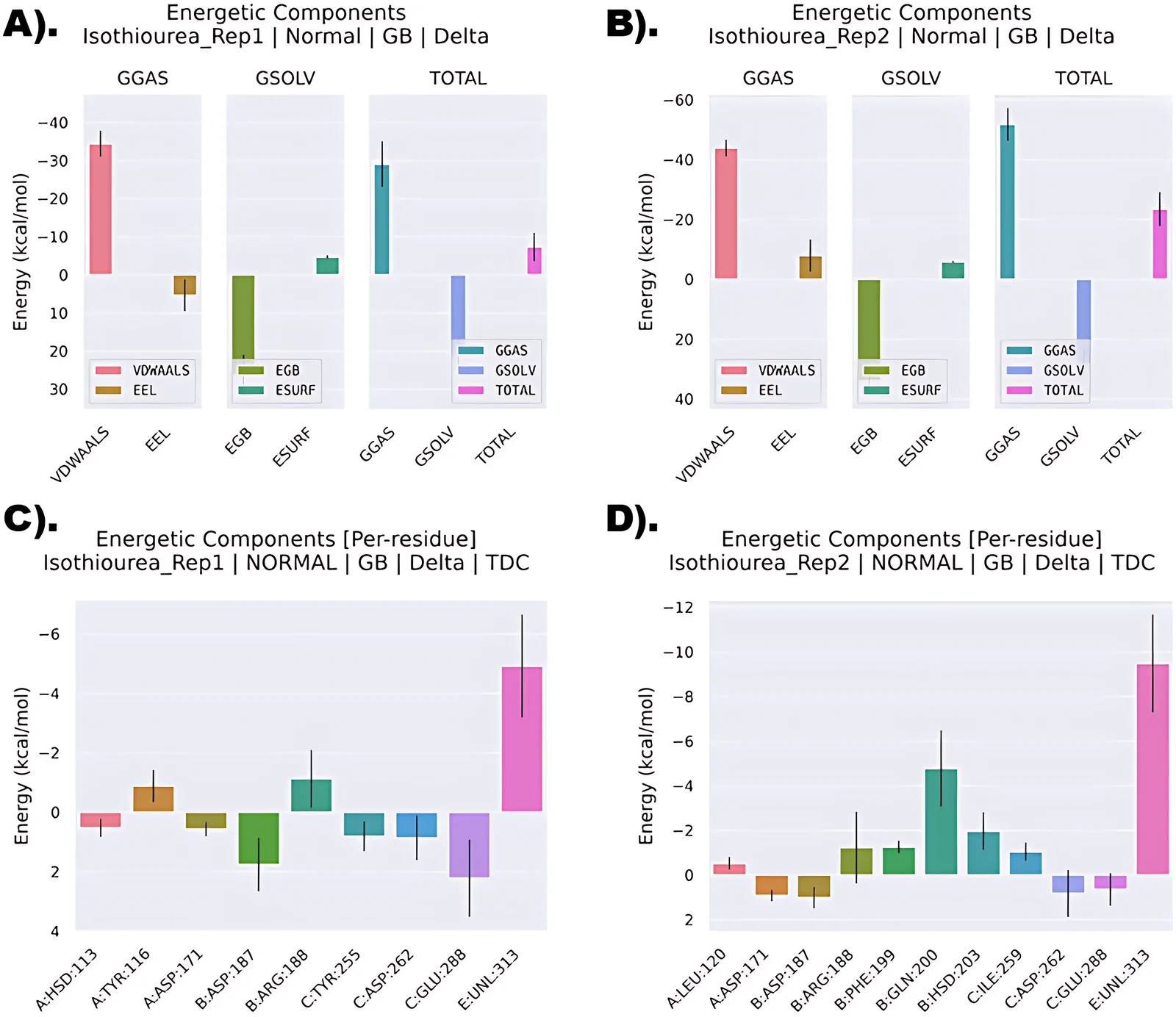
MM/GBSA and amino acid decomposition analyses of the CXCR4–isothiourea-1t complex. (A) MM/GBSA Replica 1. (B) MM/GBSA Replica 2. (C) Amino acid decomposition analysis of Replica 1. (D) Amino acid decomposition analysis of Replica 2. Negative values indicate favorable calculated contributions, whereas positive values indicate unfavorable contributions under the applied MM/GBSA model.

The amino acid decomposition analysis of Replica 1 identified Arg188 and Tyr116 as the principal favorable receptor contributors, with estimated contributions of approximately −1.1 and −0.9 kcal/mol, respectively. In contrast, Asp187 and Glu288 showed notable positive contributions, indicating unfavorable energetic effects in this replica. Smaller unfavorable contributions were also observed for His113, Asp171, Tyr255, and Asp262, as presented in Fig 16C.

**Replica 2:** In Replica 2, the MM/GBSA analysis produced a more favorable estimated total binding free energy of approximately −23 kcal/mol, suggesting stronger calculated binding than in Replica 1. The gas-phase interaction term was markedly favorable at approximately −51 kcal/mol, arising from both van der Waals and electrostatic contributions of about −44 and −8 kcal/mol, respectively. As in Replica 1, the polar-solvation term opposed binding and was partially compensated by a favorable non-polar surface term, as shown in Fig 16B.

The amino acid decomposition analysis of Replica 2 highlighted a more pronounced favorable receptor interaction pattern. Gln200 was the strongest favorable contributor, with an estimated value of approximately −4.8 kcal/mol, followed by His203, Phe199, Arg188, and Ile259, which also contributed favorably to the calculated binding energy. By contrast, Leu120, Asp171, Asp187, Asp262, and Glu288 displayed positive contributions, indicating unfavorable energetic effects in this replica, as shown in Fig 16D.

Comparison of the two replicas showed that Replica 2 had a substantially more favorable estimated total binding free energy than Replica 1, indicating a stronger calculated interaction pattern for the CXCR4–isothiourea-1t complex in the second trajectory. Across the residue-level profiles, Arg188 emerged as a recurring favorable contributor in both replicas, whereas Gln200, His203, Phe199, and Ile259 were especially important in Replica 2. In contrast, acidic residues such as Asp171, Asp187, Asp262, and Glu288 tended to contribute unfavorably. These findings indicate that the energetic profile of the reference ligand was more replica dependent than that observed for the flavonoid complexes.

When compared with apigenin and naringenin, the reference ligand showed a less favorable estimated total binding free energy in Replica 1 but a more favorable value in Replica 2. Therefore, the CXCR4–isothiourea-1t complex also exhibited trajectory-dependent energetic behavior, and its binding pattern should be interpreted comparatively rather than as a fixed affinity value.

## Discussion

Endometriosis and ovarian cancer progression remain major clinical concerns due to their multifactorial nature, shared molecular pathways, and limited effectiveness of current therapeutic strategies [42]. Endometriosis is characterized by ectopic implantation of endometrial tissue, leading to chronic inflammation, pain, and infertility, while ovarian cancer is associated with high mortality due to late diagnosis and metastasis [43,44]. Increasing evidence supports a molecular link between these two conditions, particularly through dysregulated chemokine signaling pathways [42]. Among these, the CXCR4–CXCL12 axis plays a central role in promoting cell proliferation, migration, invasion, and angiogenesis in both diseases, making it a promising therapeutic target [43,45].

The present computational pipeline integrated receptor reconstruction, ligand-guided binding-site definition, molecular docking, pharmacokinetic and toxicity predictions, pharmacophore mapping, electronic-structure calculations, replicated membrane-embedded molecular dynamics simulations, conformational analyses, and MM/GBSA calculations to prioritize a phytochemical candidate against CXCR4. The reconstructed native human CXCR4 model showed high sequence identity and near-complete template coverage, together with favorable stereochemical and non-bonded interaction-quality indicators. These validation results supported its use for the subsequent structure-based analyses. The experimentally resolved position of IT1t in the 3OE6 complex was then transferred to the reconstructed receptor through structural alignment, allowing the docking grid to be defined directly from the crystallographic ligand-binding region rather than from an independently predicted pocket.

Docking of the phytochemical library identified naringenin as the highest-ranking compound, with a predicted docking score of −6.361 kcal/mol. Apigenin showed a moderately favorable score of −5.927 kcal/mol, whereas the co-crystallized reference antagonist IT1t produced a score of −5.155 kcal/mol under the same docking conditions. Naringenin was therefore selected as the principal computational lead, while apigenin was retained as a structurally related moderate comparator and IT1t served as the crystallographic reference ligand. This three-system design enabled comparison of the top-ranked phytochemical with both a chemically related flavonoid and an experimentally resolved CXCR4 antagonist.

The residue-level docking profiles further differentiated the two flavonoids. Naringenin formed predicted conventional hydrogen bonds with Tyr116, Arg188, and Tyr255, together with aromatic interactions involving Tyr116 and Tyr255. Apigenin retained interactions with Arg188 and Tyr255 but formed a less extensive predicted interaction network. The pharmacophore maps were consistent with these docking patterns. Naringenin contained five hydrogen-bond acceptor features and supported a broader predicted polar-contact network, whereas apigenin contained more aromatic and hydrophobic features. These complementary results suggested that the prioritization of naringenin was not based solely on its docking score but also on the distribution of interaction features capable of supporting polar and aromatic recognition within the CXCR4 cavity.

The predicted ADME profiles showed that both naringenin and apigenin satisfied the evaluated rule-based drug-likeness filters, were classified as soluble, and were predicted to exhibit high gastrointestinal absorption without blood–brain barrier permeation. Their physicochemical properties were also closely comparable, making apigenin a suitable baseline compound for assessing the dynamic consequences of the structural differences between the two flavonoids. A predicted distinction was observed in P-glycoprotein behavior, as naringenin was classified as a substrate whereas apigenin was not. This result identifies possible transporter-mediated efflux as a pharmacokinetic consideration for naringenin, although it does not negate its prioritization from the receptor-binding analyses. The predicted CYP-related outcomes should be interpreted cautiously because SwissADME and ProTox apply different algorithms, training datasets, endpoint definitions, and confidence frameworks. Apparent disagreement between platforms therefore reflects model-dependent uncertainty rather than experimental confirmation of enzyme inhibition. Dedicated microsomal and recombinant-enzyme assays will be required to determine whether naringenin or apigenin meaningfully inhibits the predicted CYP isoforms.

The predicted acute oral toxicity results introduced an additional distinction between the compounds. Apigenin had a predicted LD_50_ of 2500 mg/kg and was assigned to toxicity class 5, whereas naringenin had a predicted LD_50_ of 2000 mg/kg and was assigned to toxicity class 4. Thus, apigenin showed the comparatively more favorable predicted acute-toxicity profile. However, these results did not parallel the docking ranking, demonstrating that predicted receptor interaction and predicted systemic toxicity represent separate computational dimensions. Naringenin was consequently prioritized for its predicted CXCR4 interaction profile rather than because it was predicted to be less toxic than apigenin. Both apigenin and naringenin were predicted to interact with estrogen receptor-related endpoints. Because endometriosis and several ovarian tumor subtypes are hormonally responsive, these predictions represent potential off-target liabilities rather than evidence of therapeutic benefit. The direction and biological consequence of receptor modulation cannot be inferred from the binary activity predictions and require receptor-binding, transcriptional, and estrogen-responsive proliferation assays.

The DFT analysis provided further differentiation of their electronic behavior. Naringenin displayed a wider HOMO–LUMO gap of 0.18103 a.u. compared with 0.16312 a.u. for apigenin, indicating comparatively greater predicted electronic stability under the applied calculation conditions. Apigenin showed a slightly higher dipole moment, suggesting somewhat greater overall molecular polarity. The molecular electrostatic potential maps of both compounds also showed spatially separated electron-rich and electron-deficient regions capable of supporting polar recognition. These electronic descriptors complemented the docking and pharmacophore findings but were not used independently to infer receptor-binding strength.

The replicated membrane-embedded molecular dynamics simulations provided the most detailed comparison of the three receptor–ligand systems. The CXCR4–naringenin complex showed relatively consistent protein-backbone RMSD profiles across the two 100 ns trajectories, with both replicas approaching similar final deviation ranges. Its SASA and radius-of-gyration profiles also remained within comparatively restricted intervals, indicating broadly maintained receptor surface exposure and compactness. The naringenin-bound receptor displayed localized residue flexibility, particularly around the highly mobile region near residues 220–230, but did not show widespread structural destabilization. Hydrogen bonds were dynamic in both replicas, although Replica 2 retained approximately one to two interactions throughout much of the trajectory and showed stronger late-stage persistence than Replica 1.

The CXCR4–apigenin system also maintained broadly stable SASA and radius-of-gyration profiles, but its protein-backbone RMSD values differed more clearly between the two replicas. Replica 1 sampled a higher-deviation state, whereas Replica 2 remained within a lower RMSD range. The hydrogen-bond profiles similarly showed replica-dependent persistence. These findings indicate that apigenin remained associated with a structurally maintained receptor but induced or accompanied more variable conformational sampling than naringenin across the independent trajectories.

The IT1t-bound reference complex showed the greatest variation between the early and later portions of the simulations. Both replicas underwent initial conformational adjustments and subsequently converged toward similar backbone RMSD ranges, although these values remained higher than those observed for the naringenin complex. Its hydrogen bonds were comparatively transient, particularly during the later stages of Replica 1. Nevertheless, the absence of persistent hydrogen bonding should not be interpreted as loss of binding because IT1t may also be retained through van der Waals, electrostatic, and hydrophobic interactions. The comparative structural and interaction profiles of all three systems are summarized in S2 Table.

The PCA, FEL, and DCCM analyses showed that none of the systems remained confined to a single rigid state. Instead, all three complexes sampled multiple conformational regions and metastable energy basins. The apigenin replicas differed in the breadth and compartmentalization of their sampled conformational spaces, consistent with the replica-dependent RMSD profiles. The naringenin replicas also sampled multiple states, but their global structural parameters remained comparatively consistent, suggesting that transitions within the principal-component space occurred without progressive loss of receptor compactness. The IT1t simulations showed substantial collective motions concentrated within the first two principal components and transitions between separated conformational ensembles. DCCM analysis further demonstrated that each complex retained networks of both correlated and anti-correlated residue motions, with variations in the strength and distribution of these motions across replicas.

The MM/GBSA results provided an energetic comparison consistent with the broader prioritization pattern. Naringenin produced estimated total binding free energies of approximately −20 and −25 kcal/mol in Replicas 1 and 2, respectively. These values were more favorable than those obtained for apigenin, approximately −17.3 and −20.5 kcal/mol, in the corresponding simulations. IT1t showed the greatest energetic variation, with estimated values of approximately −7 and −23 kcal/mol across its two replicas. The favorable binding estimates for all systems were driven primarily by gas-phase interactions, particularly van der Waals contributions, and were partially opposed by polar-solvation penalties.

Per-residue decomposition identified recurring receptor regions that contributed to the calculated interaction energies. His203, Leu120, Trp252, and Ile259 appeared repeatedly among the favorable contributors for naringenin, while Tyr116 was prominent in its first replica and Tyr255 appeared in the second. Apigenin showed recurring favorable contributions from Tyr116, His113, Thr117, His203, and Ile259. The IT1t complex displayed a more replica-dependent energetic network, with Arg188 contributing favorably in both trajectories and Gln200, His203, Phe199, and Ile259 becoming particularly important in Replica 2. These profiles indicate that the three ligands occupied the same general CXCR4 cavity but relied on partially different dynamic residue networks. A consolidated comparison of the replicated molecular dynamics and MM/GBSA results for the three CXCR4–ligand complexes is presented in S2 Table.

The integrated results prioritized naringenin because it combined the strongest predicted docking score, a broader predicted polar and aromatic interaction network, acceptable predicted ADME and drug-likeness properties, comparatively consistent replicated structural behavior, and the most favorable MM/GBSA estimates across both trajectories. Apigenin remained a useful comparator because of its related scaffold and similar physicochemical characteristics, but it showed a less favorable docking score, less extensive initial interaction network, and greater replica-dependent structural variation. IT1t provided an experimentally grounded reference for binding-site definition and validation but displayed more variable dynamic and energetic behavior under the reconstructed-receptor simulation conditions. Accordingly, naringenin should be described as a computationally prioritized CXCR4-binding candidate, rather than as a confirmed antagonist or experimentally validated inhibitor.

The computational prioritization of naringenin is supported by disease-relevant experimental evidence reported in cellular and animal models of endometriosis. Park et al. demonstrated that naringenin reduced the proliferation of human endometriosis-derived cells and promoted mitochondria-mediated apoptosis through loss of mitochondrial membrane potential, increased reactive oxygen species production, induction of endoplasmic-reticulum stress, activation of MAPK signaling, and suppression of the PI3K/AKT pathway [8]. These experimentally observed effects complement the present structure-based findings, in which naringenin showed the most favorable predicted docking score, a broader polar and aromatic interaction network within CXCR4, and favorable binding-energy estimates across both molecular dynamics replicas. Although the current calculations do not establish that the experimentally reported cellular effects were mediated through CXCR4, they provide a plausible target-level hypothesis through which naringenin may influence migration-, survival-, and proliferation-associated signaling.

Further support was provided by Kapoor et al., who evaluated naringenin in an experimental rat model of endometriosis and in primary endometrial cell cultures. Naringenin treatment reduced endometriotic lesion volume and weight, serum TNF-α concentrations, and histopathological scores. It also altered the expression of TAK1, PAK1, VEGF, and PCNA, modulated the Nrf2/Keap1/HO-1 axis in the endometriotic lesions, promoted apoptosis, and reduced cellular invasion through downregulation of MMP-2 and MMP-9 [9]. The reduction of VEGF and matrix metalloproteinases is particularly relevant because angiogenesis, extracellular-matrix remodeling, and invasive migration contribute to the establishment and persistence of ectopic endometriotic lesions. These experimental observations are consistent with the present pharmacophore and docking results, which indicated that naringenin possesses a combination of hydrogen-bond donor, hydrogen-bond acceptor, aromatic, and hydrophobic features capable of supporting recognition within a signaling receptor associated with cell migration. Nevertheless, the agreement should be interpreted as biological convergence rather than direct proof that naringenin inhibited CXCR4 in the reported animal model.

The relevance of naringenin also extends to ovarian cancer. Lin et al. reported that naringenin suppressed the proliferation and migration of A2780 and ES-2 ovarian cancer cells and reduced PI3K expression in vitro. In an ES-2 xenograft model, naringenin decreased tumor volume and weight, while oral administration produced stronger effects than intraperitoneal administration under the conditions examined [46]. This experimental oral activity is broadly compatible with the present SwissADME prediction of high gastrointestinal absorption. However, the predicted P-glycoprotein substrate status of naringenin indicates that transporter-mediated efflux may influence its exposure and should be examined experimentally. Therefore, the predicted ADME findings support continued investigation of oral delivery but do not establish human oral bioavailability or an effective therapeutic dose.

The functional relationship between these pharmacological effects and the selected molecular target is supported by previous studies of the CXCL12–CXCR4 axis in ovarian cancer. Guo et al. showed that CXCL12 stimulation increased the proliferation, migration, invasion, and peritoneal metastatic behavior of CXCR4-expressing ovarian cancer cells, whereas CXCR4-directed antibody treatment or the antagonist AMD3100 reduced these responses [4]. In addition, analysis of human epithelial ovarian tumors identified CXCR4 expression as a factor associated with tumor progression [47]. These findings establish CXCR4 as a biologically relevant target in ovarian cancer and provide a rationale for evaluating compounds capable of occupying its ligand-binding cavity.

Within this context, the present docking and simulation results suggest a possible molecular basis for investigating naringenin as a CXCR4-directed candidate. Naringenin formed predicted contacts with Tyr116, Arg188, and Tyr255 in the selected docking pose. During the replicated simulations, the CXCR4–naringenin system maintained broadly consistent backbone RMSD, SASA, and radius-of-gyration profiles, while His203, Leu120, Trp252, and Ile259 repeatedly contributed favorably to the calculated MM/GBSA energies. The estimated binding energies were also more favorable for naringenin than for apigenin in both corresponding replicas. These observations suggest that the initial docking contacts reorganized into a broader dynamic residue network rather than remaining limited to the original static pose.

The electronic and pharmacokinetic findings provide additional, although secondary, support for this prioritization. Naringenin had a wider predicted HOMO–LUMO gap than apigenin, indicating comparatively greater electronic stability under the applied DFT conditions, while retaining suitable polar regions for receptor recognition in the molecular electrostatic potential analysis. It also complied with the evaluated drug-likeness filters, was predicted to be soluble, and showed high gastrointestinal absorption. Its predicted LD_50_ of 2000 mg/kg and toxicity class 4 were less favorable than the corresponding apigenin estimates, demonstrating that naringenin was not selected because of predicted toxicological superiority. Instead, it was prioritized because its stronger docking performance, replicated dynamic behavior, favorable MM/GBSA estimates, and existing experimental activity in endometriosis and ovarian cancer collectively provided the most coherent evidence among the screened phytochemicals.

Importantly, the available experimental studies demonstrate the anti-proliferative, pro-apoptotic, anti-invasive, and tumor-suppressive activities of naringenin, but they do not directly demonstrate binding to or antagonism of CXCR4. The present work therefore extends the existing pharmacological evidence by proposing CXCR4 as a candidate molecular target. Confirmation will require receptor-specific binding assays, CXCL12-induced signaling experiments, CXCR4-dependent migration assays, and comparisons with established antagonists such as IT1t or plerixafor.

The present study provides a computational prioritization of naringenin as a candidate CXCR4-binding phytochemical; however, several limitations should be considered when interpreting the findings. First, the analyses were performed using a reconstructed CXCR4 model rather than a complete experimentally resolved native receptor structure. Although the model showed high sequence identity, near-complete template coverage, and favorable structural-validation parameters, uncertainties may remain in reconstructed loop regions, side-chain orientations, and local binding-site geometry. In addition, the receptor was assigned fixed protonation states representing pH 7.4; therefore, possible changes in residue protonation during ligand binding were not evaluated.

The docking results represent predicted binding poses and scoring estimates rather than experimentally measured binding affinities. AutoDock Vina scores cannot directly establish CXCR4 antagonism, and the stronger score obtained for naringenin relative to apigenin and IT1t should therefore be interpreted as a prioritization criterion only. Similarly, the pharmacophore and DFT analyses describe ligand interaction features and electronic properties but do not independently demonstrate receptor binding or biological activity. The predicted ADME and acute-toxicity outcomes were derived from computational models and require experimental confirmation. In particular, the predicted P-glycoprotein substrate status of naringenin may influence its intracellular exposure, while its predicted LD_50_ and toxicity classification cannot establish safety in humans.

The membrane-embedded molecular dynamics simulations improved the biological relevance of the study compared with simulations conducted in aqueous solvent alone. Nevertheless, each complex was evaluated using only two independent 100 ns replicas. This sampling duration and replica number may not fully capture slower receptor conformational transitions, ligand rebinding events, or rare activation-associated states. The membrane contained POPC but did not reproduce the full compositional complexity of mammalian membranes, particularly cholesterol and other lipid species known to influence GPCR organization and dynamics. Replica-dependent differences in RMSD, hydrogen bonding, conformational landscapes, and MM/GBSA energies further indicate that additional sampling is necessary before drawing firm conclusions regarding convergence.

The MM/GBSA calculations were also subject to methodological limitations. These end-state estimates depend on implicit-solvent approximations, selected dielectric constants, trajectory sampling, and ligand force-field parameters. Entropic contributions were not included, and the calculated energies should therefore not be interpreted as absolute experimental binding free energies. The residue-decomposition results identify predicted energetic contributors but do not prove that individual residues are essential for ligand binding. Moreover, the analysis focused on CXCR4 and did not assess selectivity against other chemokine receptors or unrelated molecular targets.

Future computational work should use a complete experimentally resolved native CXCR4 structure when available and examine alternative receptor conformations, protonation states, and ligand orientations. Longer simulations of at least 200–500 ns with a minimum of three independent replicas per system would provide improved conformational sampling. CXCR4 should also be simulated in more physiologically representative membranes containing cholesterol and additional phospholipid species. Enhanced-sampling methods, ligand-pocket distance analyses, ligand heavy-atom RMSD, interaction-occupancy calculations, and replicate-level statistical comparisons would further clarify ligand retention and receptor-state transitions. More rigorous free-energy methods, including alchemical calculations, could be applied to compare naringenin, apigenin, IT1t, and established CXCR4 antagonists.

Experimental validation is required to determine whether naringenin directly binds to and functionally modulates CXCR4. Receptor-binding assays, such as radioligand displacement, surface plasmon resonance, or isothermal titration calorimetry, should be used to measure binding affinity. Functional assays should evaluate CXCL12-induced calcium signaling, β-arrestin recruitment, receptor internalization, downstream AKT and ERK signaling, and CXCR4-dependent cellular migration. Site-directed mutagenesis of predicted contributors such as Tyr116, Arg188, His203, Tyr255, and Ile259 could test the residue-level interaction hypotheses generated by docking and MM/GBSA decomposition.

Subsequent biological studies should examine concentration-dependent effects of naringenin in endometriosis-derived cells and CXCR4-expressing ovarian cancer models, together with normal-cell controls. CXCR4 knockdown, knockout, or antagonist-rescue experiments would be necessary to determine whether any observed anti-proliferative, anti-invasive, or pro-apoptotic effects are specifically mediated through this receptor. Experimental pharmacokinetic studies should assess intestinal permeability, P-glycoprotein efflux, metabolic stability, cytochrome P450 interactions, plasma protein binding, and systemic exposure. Finally, efficacy and tolerability should be evaluated in relevant animal models of endometriosis and ovarian cancer before naringenin can be considered for translational development.

The present findings establish naringenin as an in silico prioritized candidate for experimental CXCR4 investigation, rather than a confirmed CXCR4 antagonist. The proposed computational and experimental studies would help determine whether the predicted interaction profile translates into reproducible receptor binding, pathway modulation, disease-relevant activity, and an acceptable pharmacological profile.

## Conclusion

This study applied an integrated computational workflow to prioritize phytochemicals targeting CXCR4 in the context of endometriosis and ovarian cancer. Among the screened compounds, naringenin showed the most favorable predicted docking score, an extensive CXCR4 interaction network, acceptable predicted ADME and drug-likeness properties, and comparatively consistent behavior across two independent 100 ns membrane-embedded molecular dynamics simulations. Its MM/GBSA estimates were also more favorable than those of apigenin across both replicas, while IT1t provided a crystallographic reference for binding-site definition and comparative evaluation. Pharmacophore and electronic-structure analyses further supported the molecular characteristics of naringenin relevant to receptor recognition. Collectively, these findings identify naringenin as an in silico prioritized CXCR4-binding candidate rather than a confirmed antagonist. Experimental binding, functional signaling, pharmacokinetic, toxicity, and disease-model studies are required to validate its predicted CXCR4-directed activity and therapeutic relevance.

## Supporting information

S1 Table Selected phytochemicals and the crystallographic reference ligand, with PubChem Compound Identifiers, sources, and reported biological roles. (DOCX)

S2 Table Comparative molecular dynamics and MM/GBSA profiles of the CXCR4 complexes with apigenin, naringenin, and IT1t across two independent 100 ns simulations. The table summarizes the replica-specific protein-backbone RMSD, residue-level RMSF, solvent-accessible surface area, radius of gyration, receptor–ligand hydrogen bonding, PCA, FEL, DCCM, estimated MM/GBSA binding energies, and major favorable residue contributions for the three CXCR4–ligand systems.
(XLSX)

## Abbreviations

ADME: absorption, distribution, metabolism, and excretion
ADMET: absorption, distribution, metabolism, excretion, and toxicity
AKT: protein kinase B
AMD3100: plerixafor
BBB: blood–brain barrier
CHARMM: Chemistry at Harvard Macromolecular Mechanics
CXCL12: C-X-C motif chemokine ligand 12
CXCR4: C-X-C chemokine receptor type 4
CYP: cytochrome P450
DCCM: dynamic cross-correlation matrix
DFT: density functional theory
ERRAT: non-bonded atomic interaction verification program
ESOL: estimated aqueous solubility
FEL: free-energy landscape
GI: gastrointestinal
GPCR: G protein-coupled receptor
GROMACS: Groningen Machine for Chemical Simulations
HOMO: highest occupied molecular orbital
IT1t: co-crystallized small-molecule CXCR4 antagonist of Protein Data Bank entry 3OE6
LD50: median lethal dose
LINCS: linear constraint solver
LUMO: lowest unoccupied molecular orbital
MAPK: mitogen-activated protein kinase
MD: molecular dynamics
MEP: molecular electrostatic potential
MMP: matrix metalloproteinase
MM/GBSA: molecular mechanics with generalized Born and surface area solvation
PAK1: p21-activated kinase 1
PCA: principal component analysis
PC1 and PC2: first and second principal components
PCNA: proliferating cell nuclear antigen
PDB: Protein Data Bank
P-gp: P-glycoprotein
PI3K: phosphoinositide 3-kinase
POPC: 1-palmitoyl-2-oleoyl-sn-glycero-3-phosphocholine
PPM: Positioning of Proteins in Membranes
QMEANDisCo: qualitative model energy analysis with distance constraints
RMSD: root-mean-square deviation
RMSF: root-mean-square fluctuation
ROS: reactive oxygen species
SASA: solvent-accessible surface area
SMILES: Simplified Molecular Input Line Entry System
TAK1: transforming growth factor-β-activated kinase 1
TIP3P: transferable intermolecular potential with three points
TNF-α: tumor necrosis factor alpha
VEGF: vascular endothelial growth factor
VEGFR: vascular endothelial growth factor receptor
