# Supplementary material for: Computational identification of natural CXCR4-binding candidates relevant to endometriosis and ovarian cancer progression
Source: PLoS One. 2026 Oct 7;21(10):e0359833. doi: 10.1371/journal.pone.0359833 (PMC13645088; doi:10.1371/journal.pone.0359833)
Supplement: Supplementary file 1 [file pone.0359833.s001.docx]

**S1 Table.** Selected phytochemicals and the crystallographic reference ligand, with PubChem Compound Identifiers, sources, and reported biological roles.

| Sr. | Compound Name | PubChem ID | 2D Structure | Compound Source | Pharmacological Activities |
| --- | --- | --- | --- | --- | --- |
| 1. | Apigenin | 5280443 |  | Parsley, celery, chamomile | Anti-cancer, anti-inflammatory, antioxidant, estrogen-modulatory |
| 2. | Baicalein | 5281605 |  | *Scutellaria baicalensis* | Anti-cancer, anti-inflammatory, antioxidant, pro-apoptotic |
| 3. | Curcumin | 969516 |  | *Curcuma longa* (turmeric rhizome) | Anti-cancer, anti-inflammatory, antioxidant, anti-angiogenic, anti-metastatic |
| 4. | Ellagic acid | 5281855 |  | Pomegranate, berries, walnuts, pecans | Anti-cancer, antioxidant, anti-inflammatory, anti-metastatic |
| 5. | Emodin | 3220 |  | Rhubarb, *Polygonum* spp., *Aloe* spp. | Anti-cancer, anti-inflammatory, antimicrobial, pro-apoptotic |
| 6. | Genistein | 5280961 |  | Soybean and soy products | Anti-cancer, anti-inflammatory, antioxidant, phytoestrogenic, anti-angiogenic |
| 7. | Honokiol | 72303 |  | *Magnolia officinalis* | Anti-cancer, anti-inflammatory, antioxidant, anti-metastatic |
| 8. | Luteolin | 5280445 |  | Celery, parsley, green pepper, thyme | Anti-cancer, anti-inflammatory, antioxidant, anti-angiogenic |
| 9. | Naringenin | 439246 |  | Citrus fruits, especially grapefruit and orange | Anti-cancer, antioxidant, anti-inflammatory, estrogen-modulatory |
| 10. | Paeonol | 11092 |  | *Paeonia suffruticosa* | Anti-cancer, anti-inflammatory, antioxidant, analgesic |
| 11. | Parthenolide | 6473881 |  | *Tanacetum parthenium* (feverfew) | Anti-cancer, anti-inflammatory, anti-migratory, pro-apoptotic |
| 12. | Quercetin | 5280343 |  | Onion, apple, berries, *Sophora japonica* | Anti-cancer, antioxidant, anti-inflammatory, anti-metastatic, anti-angiogenic |
| 13. | Resveratrol | 445154 |  | Grapes, berries, peanuts, *Polygonum cuspidatum* | Anti-cancer, anti-inflammatory, antioxidant, pro-apoptotic, chemo-sensitizing |
| 14. | Shikonin | 479503 |  | *Lithospermum erythrorhizon* | Anti-cancer, anti-inflammatory, wound-healing, pro-apoptotic |
| 15. | Sulforaphane | 5350 |  | Broccoli and other cruciferous vegetables | Anti-cancer, antioxidant, anti-inflammatory, chemo-sensitizing |
| 16. | Tanshinone IIA | 164676 |  | *Salvia miltiorrhiza* | Anti-cancer, anti-inflammatory, antioxidant, anti-fibrotic |
| 17. | Wogonin | 5281703 |  | *Scutellaria baicalensis* | Anti-cancer, anti-inflammatory, antioxidant, pro-apoptotic |
| 18. | Zerumbone | 5470187 |  | *Zingiber zerumbet* | Anti-cancer, anti-inflammatory, pro-apoptotic, anti-metastatic |
| Co-Crystal Ligand (Control) | | | | | |
| 19. | Isothiourea-1t | 25147749 |  | Synthetic | Chemokine receptor antagonist / GPCR inhibitor |
