# Supplementary material for: Computational identification of natural CXCR4-binding candidates relevant to endometriosis and ovarian cancer progression
Source: PLoS One. 2026 Oct 7;21(10):e0359833. doi: 10.1371/journal.pone.0359833 (PMC13645088; doi:10.1371/journal.pone.0359833)
Supplement: Supplementary file 2 [file pone.0359833.s003.docx]

PONE-D-26-26813

Computational Identification of Natural inhibitor Targeting CXCR4 in Endometriosis and Ovarian Cancer Progression

Dear Editor,

The authors have carried out different types of computational calculations to determine various parameters in the course of selecting a natural molecule capable of inhibiting a target protein. All the steps seems to be routine in nature with thorough discussion of the obtained data. Following points were derived after reading the manuscript-

1. The language and flow of information is good. However, some errors are present. Eg. Figure 6A, the unit is ps. Symbol for Angstrom was not used.
2. The native ligand of the protein crystal has been used as control. Please use a drug (FDA approved) for this purpose. Comparing the results of the hit molecule with the native ligand and other approved drug would be more convincing.
3. Superimposition of the pose of native ligand in the crystal structure with the one obtained from calculation was not shown. An RMSD of 0.026 Å is to be verified by figure and by the process. How was it derived?
4. The sampling of the chemical space seems to be tiny. Please justify.
5. Exhaustiveness of 8 was chosen. Please provide the results of convergence studies. The spacing of 1 Å seems to be large. Please explain these selections.
6. 12^th^ molecule in terms of binding affinity was chosen as a hit. This seems strange. The discussion is not enough or correct based on the obtained results. Please revise as the bias is not scientifically appropriate.
7. “The control ligand instead interacted with residues such as ARG188, TYR116, VAL196, ILE259, and PHE199, suggesting a somewhat different binding orientation inside the same pocket.”. Please explain why were the residues not same as that seen with the hit ligand. The explanation is not that clear.
8. Caption of Figure 3C and 3D are interchanged. Please adjust.
9. 11 amino acid residues are shown for the native ligand interacting with the protein in Figure 3 but only 6 are provided in the table. Please clarify.
10. “Its interaction pattern was more structurally elaborate than the control, with a combination of short distance hydrogen bonding and extensive aromatic stabilization.” The results from molecular docking could be fleeting in nature and cannot be taken for sure. Structurally elaborate does not make the adduct stable in nature. This interpretation is to be supported by appropriate references for reliability.
11. RMSD has been calculated. Is it ligand RMSD relative to protein backbone or protein backbone RMSD relative to the protein backbone? Without this distinction the results carry little to no meaning.
12. How do the result (RMSD) compare with the apo form of the protein? This would help to discern the effect of ligand binding.
13. After about 380 ns the equilibrium is lost in the production run as seen in the RMSD plot of the luteolin-protein complex. This hints that the adduct is not stable with time. Please rationalize the data.
14. RMSD > 12 Å is well above the accepted range. Please add relevant references supporting the claims.
15. Please add an image from the trajectory showing that the docked luteolin is still at the active site of the protein even at 500 ns of simulation run. It will prove the stability of the protein-ligand complex at physiological conditions. Without the picture the extent of the conservation of the pose cannot be confirmed.
16. Please fix the discontinuity in Figure 5B.
17. The protein is the same but still the gyradius and SASA are different for two systems as seen in Figure 6. Please provide sufficient explanation.
18. Please add scree plot in PCA section. Why were only two components chosen?
19. How is MMGBSA better than MMPBSA? Why was the former chosen?
20. Please justify the choice of exchange correlation functional and the basis set. Why is DFT at 0 Kelvin appropriate to model the system at 310 Kelvin?
21. HOMO-LUMO gap of 4.35 eV means the molecule is an insulator? How is electron transfer capacity related to adduct formation? Please add a strong reference for support.
22. “A relatively moderate energy gap suggests that the molecule can participate in charge transfer interactions without being overly reactive or unstable, which is favorable for ligand binding.” This statement is vague. No redox reaction could be seen for ligand binding with protein. How is this correct?
23. “The dipole moment of luteolin was calculated to be 4.2384 Debye, reflecting a balanced polarity that supports both hydrophilic and hydrophobic interactions.” The polarity is high. The molecule has electronegative centers making it hydrophilic. How is the dipole moment related to hydrophobic nature? Please add more discussion for supporting the claim.
24. ADMET of just one molecule is not sufficient. Please revise.
25. Stopped here.

Considering all the above points, it seems obvious that the manuscript is premature and lacks methodological rigor. The bias nature of choosing just one molecule seems to be unfounded. I would not recommend for publication in its current form as the hit molecule is not uncommon. The data interpretation is not sufficient to be scientifically convincing. I could not be more positive than this.

Thank you.

Reviewer
