# Supplementary material for: Computational identification of natural CXCR4-binding candidates relevant to endometriosis and ovarian cancer progression
Source: PLoS One. 2026 Oct 7;21(10):e0359833. doi: 10.1371/journal.pone.0359833 (PMC13645088; doi:10.1371/journal.pone.0359833)
Supplement: Supplementary file 3 [file pone.0359833.s005.docx]

**Point by point response**

We sincerely thank the Editor and all reviewers for their careful evaluation of our manuscript and for providing us with the opportunity to undertake a comprehensive revision. Their constructive comments identified important limitations in the original receptor preparation, docking setup, lead-selection strategy, and molecular dynamics workflow, and these concerns guided a substantial reanalysis of the study. Rather than making only textual changes, we reconstructed the native CXCR4 receptor without the T4 lysozyme fusion, corrected the ligand-guided docking grid, redocked the complete phytochemical library, and repeated the molecular dynamics analyses in an explicit membrane environment using independent replicas. Importantly, the principal lead was changed from luteolin to naringenin because the corrected docking analysis ranked naringenin highest among the screened phytochemicals, whereas luteolin was no longer supported as the preferred candidate. Naringenin was subsequently evaluated through comparative ADME, toxicity, pharmacophore, DFT, membrane-embedded molecular dynamics, and MM/GBSA analyses. Apigenin was retained as a structurally related moderate comparator, while IT1t served as the crystallographic reference ligand. The revised manuscript now presents naringenin cautiously as an in silico prioritized CXCR4-binding candidate and avoids claims of experimentally confirmed antagonism, therapeutic efficacy, or safety.

**Journal Requirements:**

When submitting your revision, we need you to address these additional requirements.

1.Please ensure that your manuscript meets PLOS ONE's style requirements, including those for file naming. The PLOS ONE style templates can be found at

<https://journals.plos.org/plosone/s/file?id=wjVg/PLOSOne_formatting_sample_main_body.pdf>

[https://journals.plos.org/plosone/s/file?id=ba62/PLOSOne_formatting_sample_title_authors_affiliations.pdf](https://track.editorialmanager.com/CL0/https:%2F%2Fjournals.plos.org%2Fplosone%2Fs%2Ffile%3Fid=ba62%2FPLOSOne_formatting_sample_title_authors_affiliations.pdf/1/010f019f3bee1fd3-d56d550b-8dc4-48cf-984f-67c286358ec1-000000/N24Ggz45A3A35LA4n4w1M8Ux0RgRmlILOmHPyLUXi_E=258)

**Response: Thank you for highlighting the PLOS ONE formatting requirements. The revised manuscript and title page have been formatted in accordance with the journal’s style templates. The manuscript structure, headings, author affiliations, tables, references, figure legends, and in-text figure and table citations have been reviewed for consistency with PLOS ONE requirements. All figures have been removed from the main manuscript file and uploaded separately as individual, sequentially named figure files, while their corresponding legends remain in the manuscript. Journal-specific declarations have also been removed from the manuscript and entered in the relevant fields of the online submission system.**

2. Please note that PLOS One has specific guidelines on code sharing for submissions in which author-generated code underpins the findings in the manuscript. In these cases, we expect all author-generated code to be made available without restrictions upon publication of the work. Please review our guidelines at [https://PLOS](https://plos/) One%/s/materials-and-software-sharing#loc-sharing-code and ensure that your code is shared in a way that follows best practice and facilitates reproducibility and reuse.

**Response: Thank you for drawing our attention to the PLOS ONE code-sharing policy. No new software package or original computational algorithm was developed specifically for this study; the analyses were performed using established third-party computational tools, with their versions and principal parameters reported in the Methods section. To facilitate reproducibility and reuse, all author-prepared analysis scripts, input and configuration files, relevant structural files, and supporting computational outputs have been made publicly available without restriction through the Zenodo repository identified in the Data Availability Statement. Documentation describing the deposited files and their relationship to the reported analyses has also been provided in the repository.**

3. Thank you for stating the following in the Competing Interests section:

“The authors have declared that no competing interests exist.”

We note that one or more of the authors are employed by a commercial company: Özel Nev Hospital, Sanliurfa, Türkiye

4. Please provide an amended Funding Statement declaring this commercial affiliation, as well as a statement regarding the Role of Funders in your study. If the funding organization did not play a role in the study design, data collection and analysis, decision to publish, or preparation of the manuscript and only provided financial support in the form of authors' salaries and/or research materials, please review your statements relating to the author contributions, and ensure you have specifically and accurately indicated the role(s) that these authors had in your study. You can update author roles in the Author Contributions section of the online submission form.

**Response: Thank you for identifying the commercial affiliation of Dr. Sinan Karageçili and Dr. Deniz Inan with Özel Nev Hospital, Şanlıurfa, Türkiye. We have updated the Competing Interests declaration in the online submission system to transparently disclose this employment relationship. Özel Nev Hospital did not fund, sponsor, or influence the present study and had no role in the study design, data collection and analysis, interpretation of the findings, decision to publish, or preparation of the manuscript. This commercial affiliation does not alter the authors’ adherence to PLOS ONE policies on sharing data and materials.**

**Reviewer #1:**

The manuscript investigates the in-silico identification of natural CXCR4 antagonists to address shared molecular mechanisms in endometriosis and ovarian cancer progression. While the pathological rationale linking the CXCL12/CXCR4 biological axis to both diseases is sound and clinically relevant, the computational execution of the study is fatally compromised.

A rigorous biophysical analysis reveals catastrophic errors in structural preparation, system equilibration, and molecular docking. The authors simulated a transmembrane G protein-coupled receptor (GPCR) in a water-only environment, retained a massive crystallographic fusion partner, and performed docking with a coordinate sign-flip error.

These cumulative errors caused full structural denaturation, which the authors mischaracterize as "adaptive flexibility". Consequently, the reported binding energies, trajectory metrics, and thermodynamic predictions are unscientific artifacts that do not reflect a physiologically realistic state of the receptor.

The title clearly identifies the therapeutic target and disease contexts. However, the abstract reports a backbone root-mean-square deviation (RMSD) of 2.1 to 2.4 nm (21 to 24 Å) for the CXCR4-luteolin complex. In GPCR biophysics, an RMSD of this magnitude indicates complete denaturation and unfolding of the transmembrane domain rather than stable, adaptive flexibility. This structural collapse is further supported by the highly swollen radius of gyration (Rg ~ 3.0 nm) and elevated solvent-accessible surface area (SASA ~ 250 to 255 nm²). The introduction provides a reasonable pathological overview of CXCR4 in gynecological pathologies. However, it fails to discuss the established landscape of CXCR4 antagonists (such as Plerixafor or IT1t) and provides no biochemical justification for curating the specific panel of eighteen phytochemicals.

**Response: We sincerely thank the reviewer for the rigorous biophysical assessment of the original submission. We agree that the original use of the T4 lysozyme-containing crystallographic construct, the incorrect docking-grid coordinate, and the aqueous simulation environment prevented reliable interpretation of the reported structural and energetic results. We therefore did not attempt to defend or reinterpret the original luteolin trajectories. Instead, the receptor preparation, docking, lead selection, molecular dynamics simulations, and post-simulation analyses were comprehensively repeated.**

**In the revised manuscript, the T4 lysozyme fusion was excluded and the native human CXCR4 sequence, including intracellular loop 3, was reconstructed using SWISS-MODEL. The binding site was redefined from the experimentally resolved IT1t position using the corrected positive Z coordinate, and the complete ligand panel was redocked. The previous water-only 500 ns simulations and all associated RMSD, RMSF, SASA, radius-of-gyration, PCA, FEL, DCCM, and MM/GBSA results were removed. New membrane-embedded simulations were conducted for the CXCR4 complexes with naringenin, apigenin, and IT1t using two independently seeded 100 ns replicas per complex. The title, abstract, Introduction, Results, Discussion, and Conclusion were also rewritten to reflect the revised workflow and to describe naringenin only as an in silico prioritized CXCR4-binding candidate, not as a confirmed antagonist.**

**The Introduction now discusses the established CXCR4 antagonist landscape, including plerixafor and IT1t, and explains that the 18 phytochemicals were selected based on peer-reviewed experimental evidence relevant to inflammatory signaling, proliferation, apoptosis, invasion, angiogenesis, and oxidative-stress-associated processes in endometriosis or ovarian cancer.**

Following are the Critical Methodological Flaws

The methodology contains three fatal issues that invalidate the study's conclusions:

1. Retained Crystallographic Chimera (T4 Lysozyme): The crystal structure of human CXCR4 (PDB: 3OE6) was engineered as a chimera where the third intracellular loop (ICL3) was replaced with a bacteriophage T4 Lysozyme (T4L) domain (residues 900–1201) to facilitate crystallization. The authors neglected to remove this domain or model the native loop. Retaining a highly basic bacterial protein on the intracellular face introduces severe steric and electrostatic artifacts. This oversight is exposed by residue indices extending up to 1100 in the RMSF and cross-correlation plots, whereas monomeric human CXCR4 contains only 352 residues.

**Response: We agree with the reviewer. The original crystallographic construct was unsuitable for direct downstream modelling because PDB ID 3OE6 contains an engineered T4 lysozyme fusion in place of the native third intracellular loop. In the revised workflow, the crystallographic chimera was not used directly for docking or molecular dynamics simulations. The T4 lysozyme sequence was excluded, and the native human CXCR4 amino-acid sequence, including intracellular loop 3, was used as the modelling target in SWISS-MODEL.**

**The selected reconstructed model contained 306 CXCR4 residues and did not contain the T4 lysozyme fusion partner or the co-crystallized ligand. The model showed 98.2% template coverage, 100% sequence identity across the aligned region, a GMQE of 0.92, and a QMEANDisCo global score of 0.89 ± 0.05. PROCHECK and ERRAT validation were also performed before receptor preparation. Consequently, all revised RMSF, PCA, and DCCM analyses now use the native CXCR4 residue range and no longer contain residue indices extending above 1000.**

2. Docking Coordinate Sign-Flip Error: The authors report setting the docking grid center to X = 19.7 Å, Y = 30.2 Å, and Z = -71.0 Å. However, the true coordinates of the predicted active pocket (Pocket 1) have a positive Z-coordinate of +71.09 Å. This sign-flip of the Z-coordinate represents a physical displacement of 142 Å. This error placed the docking grid entirely outside the receptor's physical boundaries, meaning the docking was executed in empty vacuum or on the non-functional surface of the T4L chimera.

**Response: We agree that the previously reported negative Z coordinate placed the docking grid incorrectly. The earlier PrankWeb-based grid definition and its associated docking results were therefore discarded.**

**In the revised workflow, the binding site was defined directly from the experimentally resolved position of the co-crystallized IT1t ligand in PDB ID 3OE6. The reconstructed, protonated, and energy-minimized CXCR4 model was structurally aligned with the CXCR4 component of the original 3OE6–IT1t complex in Discovery Studio Visualizer. The geometric centre of the aligned IT1t ligand was determined as X = 17.6 Å, Y = 28.8 Å, and Z = +73.4 Å, and a grid of 20 × 20 × 20.1 Å was used. The grid was visually inspected to confirm that it encompassed the complete IT1t position and the surrounding transmembrane binding cavity. The complete phytochemical library and IT1t were then redocked using the corrected ligand-guided grid.**

3. The Water-Only GPCR Simulation Fatality: The authors executed a 500 ns molecular dynamics simulation in explicit water without a lipid membrane bilayer. CXCR4 is an integral membrane protein with a highly hydrophobic outer surface. Simulating this receptor in water exposes these non-polar helices to polar water molecules. The hydrophobic effect immediately drives the rapid collapse and denaturation of the helical bundle as the system minimizes unfavorable water interactions.

**Response: We agree that the original aqueous simulation environment was inappropriate for a seven-transmembrane GPCR. The original 500 ns water-only trajectories and all analyses derived from them were removed from the manuscript.**

**The complete molecular dynamics workflow was repeated with CXCR4 embedded in an explicit pre-equilibrated POPC membrane. Each CXCR4–ligand complex was oriented using the PPM server and incorporated into a symmetric membrane containing 204 POPC molecules using CHARMM-GUI Membrane Builder. The systems were solvated with CHARMM-compatible TIP3P water, neutralized with Na⁺ and Cl⁻ ions, and adjusted to 0.15 M ionic strength. The CHARMM36m force field was used for CXCR4, CHARMM36 for the membrane, and CGenFF for the ligands. Each system underwent energy minimization and the standard six-step CHARMM-GUI membrane-equilibration protocol before production simulation.**

**We acknowledge that the revised membrane contained POPC but not cholesterol. This is now stated explicitly in the limitations, together with the need for future simulations using mixed POPC/cholesterol and more physiologically representative membrane compositions.**

4. Docking Validation Failure: The authors claim a docking validation RMSD of 0.026 Å but fail to reproduce the native binding mode of the control ligand Isothiourea-1t (IT1t). In the crystal structure, IT1t forms critical salt bridges with Asp97 and Glu288. The reported docked pose completely misses these electrostatic anchors.

**Response: We agree that the previously reported RMSD of 0.026 Å and the associated validation interpretation were not reliable. The previous redocking result was removed, and docking validation was repeated after reconstructing the native receptor and correcting the ligand-guided docking grid.**

**IT1t was extracted from the original 3OE6 complex and redocked into the corrected binding cavity using the same parameters applied to the phytochemical library. The crystallographic and redocked poses were superimposed, and the revised heavy-atom RMSD calculated with DockRMSD was 1.364 Å. The crystallographic and redocked orientations are now presented in Figure 5A and Figure 5B, respectively.**

**The revised manuscript does not claim that every specific crystallographic contact, including the individual Asp97 and Glu288 interactions, was exactly reproduced. The validation is limited to reproduction of the overall crystallographic pose, supported by the reported heavy-atom RMSD and visual comparison. The language concerning docking validation has therefore been moderated accordingly.**

5. Arbitrary Compound Prioritization: Curation of luteolin (binding affinity -5.650 kcal/mol) as the single lead is highly arbitrary. Multiple screened phytochemicals (e.g., naringenin at -6.361 kcal/mol, zerumbone at -6.301 kcal/mol, resveratrol at -6.250 kcal/mol) demonstrated significantly stronger predicted binding energies than luteolin.

**Response: We agree with the reviewer that the previous prioritization of luteolin was not supported by the docking ranking. The complete ligand panel was redocked using the corrected native receptor and ligand-guided binding cavity. Following this reanalysis, luteolin was not retained as the lead and was not subjected to comparative pharmacophore, DFT, molecular dynamics, or MM/GBSA analyses.**

**Naringenin achieved the most favorable predicted docking score among the screened phytochemicals at −6.361 kcal/mol, compared with −6.301 kcal/mol for zerumbone, −6.250 kcal/mol for resveratrol, and −5.650 kcal/mol for luteolin. Naringenin was therefore selected as the primary computational lead. Apigenin, with a moderate score of −5.927 kcal/mol, was included as a structurally related flavonoid comparator, while IT1t was used as the crystallographic reference ligand.**

**The prioritization of naringenin was subsequently evaluated using its residue-level docking interactions, predicted ADME and toxicity profile, pharmacophore features, electronic properties, two independent membrane-embedded simulations, and MM/GBSA estimates. Thus, the revised selection is based on the integrated workflow rather than on an arbitrary preference for luteolin.**

6. The Estrogen Receptor Alpha (ERα) Paradox: Toxicity profiling predicted luteolin to be active against ERα with a probability of 0.76. Endometriosis is a chronic, estrogen-dependent inflammatory disorder. Clinical therapies focus on suppressing estrogen signaling. Recommending an ERα agonist raises a severe biological contraindication—exacerbating ectopic tissue growth—which the authors fail to discuss.

**Response: We agree that recommending luteolin without addressing its predicted estrogen-receptor activity was inappropriate in the context of an estrogen-dependent disorder. Following the complete reanalysis, luteolin was removed from lead prioritization and is retained only as one member of the initial 18-compound screening panel. No therapeutic recommendation, advanced pharmacophore analysis, DFT analysis, molecular dynamics simulation, or MM/GBSA analysis is now presented for luteolin.**

**The revised comparative toxicity analysis focuses on naringenin and apigenin and reports their predicted nuclear-receptor-related endpoints transparently in Table 6. These predictions are treated as model-based alerts rather than evidence of agonism, antagonism, therapeutic benefit, or clinical safety. The manuscript also clearly states that the selected compounds require experimental pharmacological and toxicological validation before their biological relevance can be established.**

7. PAINS and Redox Liabilities: Luteolin is a well-known Pan-Assay Interference Compound (PAINS). Its catechol moiety is prone to auto-oxidation, reactive oxygen species generation, and non-specific colloidal aggregation, which cause false-positive results in cell-free biochemical assays.

**Response: We agree with the reviewer’s concern regarding the PAINS and redox liabilities associated with luteolin. Because the corrected docking workflow did not support luteolin as the principal candidate, it was removed from all lead-specific analyses and conclusions. Luteolin now appears only in the initial screening table and is not proposed as a CXCR4-directed lead.**

**Accordingly, the revised manuscript no longer bases its conclusions on luteolin or recommends its experimental advancement. Naringenin was instead prioritized following the corrected docking analysis and was comparatively evaluated against apigenin and IT1t using pharmacokinetic, toxicity, pharmacophore, DFT, replicated membrane-dynamics, and MM/GBSA analyses.**

8. Modern computational biology requires independent, replicated molecular dynamics runs (minimum of three replicates starting with randomized velocity seeds) to confirm conformational convergence. Reporting a single 500 ns trajectory of an unfolding chimeric protein in water means all post-simulation analyses—including free energy landscapes, PCA, DCCM, and MMGBSA binding energies—are physically meaningless artifacts of protein denaturation.

**Response: We agree that the original single 500 ns trajectory could not establish reproducibility and that all post-simulation analyses derived from the structurally inappropriate aqueous system required replacement. The original trajectory and all associated analyses were therefore removed.**

**In the revised study, three receptor–ligand systems were evaluated: CXCR4–naringenin, CXCR4–apigenin, and CXCR4–IT1t. Each system was simulated for 100 ns in two independent membrane-embedded replicas initiated with different randomized velocity seeds. This resulted in six production trajectories and an aggregate production time of 600 ns. RMSD, RMSF, SASA, radius of gyration, receptor–ligand hydrogen bonds, PCA, FEL, DCCM, MM/GBSA, and per-residue decomposition analyses were performed separately for each replica.**

**We acknowledge that two replicas remain below the reviewer’s recommended minimum of three. We have therefore not claimed definitive statistical convergence or complete removal of stochastic uncertainty. Replica-level differences are reported transparently, all comparisons are interpreted descriptively, and the need for at least three longer independent trajectories is explicitly stated as a future requirement in the limitations section.**

**Mandatory Revisions:**

1. Critical Environment Correction: Re-run the entire molecular dynamics simulation pipeline. The receptor must be embedded in a pre-equilibrated POPC/cholesterol lipid bilayer to stabilize the transmembrane helices.

**Response: The entire molecular dynamics workflow was repeated in an explicit pre-equilibrated lipid membrane rather than in water alone. Each CXCR4–ligand complex was embedded in a symmetric POPC bilayer containing 204 lipid molecules using CHARMM-GUI, followed by solvation, ion addition, energy minimization, six-stage membrane equilibration, and production simulation under semi-isotropic NPT conditions.**

**Cholesterol was not included in the revised membrane. We have not claimed that the POPC-only system reproduces the full compositional complexity of a mammalian membrane. This remaining limitation is explicitly acknowledged in the revised Discussion, and mixed POPC/cholesterol membranes are identified as an important requirement for future simulations.**

2. Critical Structure Correction: Delete the T4 Lysozyme domain (residues 900–1201 in 3OE6) and reconstruct the native intracellular loop 3 (ICL3) using homology loop modeling software before initiating docking.

**Response: This revision was implemented. The T4 lysozyme-containing 3OE6 crystallographic construct was not used directly for docking or molecular dynamics simulations. The T4 lysozyme sequence was excluded, and a native human CXCR4 model containing intracellular loop 3 was reconstructed using SWISS-MODEL. The selected model was evaluated using GMQE, QMEANDisCo, PROCHECK, and ERRAT before receptor preparation. All downstream analyses were performed using the reconstructed 306-residue CXCR4 model.**

3. Critical Docking Correction: Correct the Z-axis coordinate sign-flip error (+71.09 Å instead of -71.0 Å) and re-dock the entire ligand panel in the true binding pocket.

**Response: This revision was implemented. The previous negative Z coordinate and PrankWeb-derived setup were discarded. The corrected docking grid was defined using the geometric centre of the aligned crystallographic IT1t ligand at X = 17.6 Å, Y = 28.8 Å, and Z = +73.4 Å. The complete phytochemical panel and IT1t reference ligand were redocked using this ligand-guided binding site. The revised docking results, interaction analyses, lead ranking, and IT1t redocking validation now derive exclusively from the corrected workflow.**

4. Implement a minimum of three independent, membrane-bound MD trajectories starting with randomized velocity seeds to ensure statistical reproducibility.

**Response: We agree with the reviewer that three or more independent trajectories would provide stronger reproducibility. In the revised study, computational-resource limitations permitted completion of two independently seeded 100 ns membrane-bound replicas for each of three complexes, producing six trajectories and 600 ns of aggregate production sampling.**

**We have transparently reported this design and have not described it as definitive proof of convergence. All analyses were performed and presented separately for Replica 1 and Replica 2, and the Discussion explicitly identifies the use of only two replicas as a limitation. Longer simulations with a minimum of three independent replicas per system are specified as a future priority.**

5. Add dedicated discussion sections addressing: (a) the clinical paradox of utilizing an ERα agonist in endometriosis, (b) the PAINS status of luteolin and biochemical strategies to rule out false positives, and (c) the thermodynamic justification for prioritizing luteolin over stronger-binding candidate compounds.

**Response: The reviewer’s three concerns were addressed primarily by removing luteolin from lead prioritization following complete reanalysis of the corrected docking workflow.**

**First, luteolin is no longer recommended as a CXCR4-directed candidate, and its predicted ERα activity is no longer used to support any therapeutic conclusion. Second, luteolin was not advanced to pharmacophore, DFT, molecular dynamics, or MM/GBSA analyses, so its PAINS and redox liabilities no longer affect the principal interpretation of the study. Third, the revised manuscript does not attempt to provide a thermodynamic justification for selecting luteolin over stronger-scoring compounds because luteolin was not selected.**

**Naringenin was prioritized because it achieved the most favorable corrected docking score among the screened phytochemicals and subsequently showed a broader predicted CXCR4 interaction network, acceptable predicted ADME properties, comparatively consistent behavior across two membrane-embedded replicas, and more favorable MM/GBSA estimates than apigenin in both corresponding trajectories. Apigenin was retained as a structurally and physiochemically related moderate comparator, while IT1t served as the crystallographic reference. The revised Discussion also emphasizes that this integrated computational evidence supports only the prioritization of naringenin for experimental evaluation and does not establish direct CXCR4 antagonism, therapeutic efficacy, or safety.**

**Reviewer #2:**

The writing is up to the mark but the methodological rigor is below average. Over interpretation of data and the biasness has rendered the manuscript unsuitable in its current form. Relevant references to support the claims would be required.

**Response: We thank the reviewer for this important assessment. We agree that the original manuscript lacked sufficient methodological rigor and contained interpretations that exceeded the strength of the computational evidence. The study was therefore comprehensively revised rather than being limited to editorial changes.**

**The crystallographic T4 lysozyme fusion construct was replaced with a reconstructed native human CXCR4 model containing intracellular loop 3. The corrected receptor model was structurally evaluated using SWISS-MODEL quality estimates, PROCHECK, and ERRAT before downstream analyses. The docking grid was redefined from the experimentally resolved position of the co-crystallized IT1t ligand using the corrected positive Z coordinate, and the complete phytochemical panel was redocked. The docking protocol was revalidated through IT1t redocking, producing a revised heavy-atom RMSD of 1.364 Å.**

**The original water-only molecular dynamics simulation and all associated post-simulation results were removed. New membrane-embedded simulations were conducted for the CXCR4 complexes with naringenin, apigenin, and IT1t in an explicit POPC bilayer. Two independently seeded 100 ns replicas were completed for each complex, and RMSD, RMSF, SASA, radius of gyration, hydrogen bonding, PCA, FEL, DCCM, MM/GBSA, and per-residue decomposition analyses were evaluated separately for each replica. Replica-dependent differences are now reported transparently, and the manuscript does not claim definitive statistical convergence.**

**The previous arbitrary prioritization of luteolin was also corrected. Naringenin was selected as the principal computational lead because it achieved the most favourable predicted docking score and was subsequently evaluated together with the structurally related comparator apigenin and the crystallographic reference IT1t. Candidate selection was based on the integrated docking, interaction, ADME, toxicity, pharmacophore, DFT, replicated molecular dynamics, and MM/GBSA results rather than docking score alone.**

**To address overinterpretation and potential bias, definitive terms such as “confirmed antagonist,” “stable inhibitor,” “therapeutically effective,” and “clinically safe” were removed. The revised manuscript consistently describes naringenin as an in silico prioritized CXCR4-binding candidate and explicitly states that docking scores, predicted toxicity, MM/GBSA energies, and molecular dynamics profiles do not establish experimental binding, receptor antagonism, efficacy, or safety. The limitations section now discusses the reconstructed receptor, POPC-only membrane composition, use of two replicas, force-field and MM/GBSA approximations, and absence of biochemical, cellular, pharmacokinetic, and in vivo validation.**

**Relevant peer-reviewed references were added throughout the Introduction and Discussion to support the biological relevance of the CXCL12–CXCR4 axis, the established CXCR4 antagonist landscape, the selection of the phytochemical panel, and the experimentally reported pharmacological activities of naringenin in endometriosis and ovarian cancer models. The revised Discussion clearly distinguishes published experimental evidence from the target-level hypotheses generated by the present computational study.**

**Reviewer #3:**

**Revision Main Points**

1. Rationale for lead selection: The authors screened 18 phytochemicals and naringenin (-6.361 kcal/mol) and zerumbone (-6.301 kcal/mol) exhibited significantly more favorable docking scores than luteolin (-5.650 kcal/mol).

**Response: We thank the reviewer for identifying the inconsistency in the original lead-selection strategy. We agree that the previous selection of luteolin was not supported by the docking ranking. The complete phytochemical panel was therefore redocked after correction of the receptor structure and docking-grid coordinates, and luteolin was not retained as the lead.**

**In the revised analysis, naringenin achieved the most favorable predicted docking score among the 18 phytochemicals at −6.361 kcal/mol, compared with −6.301 kcal/mol for zerumbone, −6.250 kcal/mol for resveratrol, and −5.650 kcal/mol for luteolin. Naringenin was consequently selected as the principal computational lead. Apigenin, with a moderate docking score of −5.927 kcal/mol, was included as a structurally related flavonoid comparator, while IT1t was retained as the crystallographic reference ligand. The revised manuscript no longer presents luteolin as the prioritized compound.**

2. The authors mention that docking scores are not the only criteria for lead quality, but no explicit data (e.g. initial MD or toxicity filters) is provided to justify why the top scoring compounds were skipped in favor of luteolin for the 500 ns simulation.

**Response: We agree with the reviewer. The original manuscript did not provide sufficient evidence to justify advancing luteolin instead of the higher-ranking compounds. Rather than attempting to retrospectively defend that selection, we repeated the docking analysis using the corrected native CXCR4 model and ligand-guided binding pocket and removed luteolin from all lead-specific analyses.**

**Naringenin was advanced because it ranked first in the corrected docking screen and also demonstrated an extensive predicted interaction network involving Tyr116, Arg188, and Tyr255. Its prioritization was subsequently evaluated using predicted ADME and toxicity properties, pharmacophore characterization, DFT calculations, two independent membrane-embedded molecular dynamics replicas, and MM/GBSA analysis. Thus, the revised lead selection is supported by an integrated comparative workflow rather than by docking score alone.**

3. The manuscript would be improved by a clearer rationale or a comparative MD study of the top three candidates.

**Response: We thank the reviewer for this recommendation. A comparative molecular dynamics design was implemented, although the systems were selected to answer complementary questions rather than simply simulating the three highest docking scores.**

**The revised study evaluates:**

- **naringenin as the highest-ranking phytochemical and principal computational lead;**
- **apigenin as a structurally and physiochemically related moderate-scoring flavonoid comparator; and**
- **IT1t as the experimentally resolved crystallographic reference ligand.**

**Apigenin was selected instead of an unrelated high-ranking compound because it has a closely related flavonoid scaffold, comparable molecular weight, hydrogen-bonding capacity, lipophilicity, solubility, gastrointestinal absorption, and drug-likeness profile. This enabled a more controlled comparison of how relatively subtle structural differences influenced CXCR4 interactions and dynamic behavior. Each of the three complexes was evaluated using two independently seeded 100 ns membrane-embedded simulations, followed by RMSD, RMSF, SASA, radius-of-gyration, hydrogen-bond, PCA, FEL, DCCM, MM/GBSA, and per-residue decomposition analyses.**

4. Simulation Environment (Lipid Bilayer): The study correctly identifies CXCR4 as a membrane signaling protein (GPCR). But the methodology mentions that the protein-ligand complex is placed in a cubic simulation box containing water.

**Response: We agree that the original aqueous simulation environment was inappropriate for CXCR4, which is a seven-transmembrane GPCR. The original water-only 500 ns trajectories and all results derived from them were removed.**

**In the revised workflow, each CXCR4–ligand complex was oriented using the PPM server and embedded in a symmetric, pre-equilibrated POPC lipid bilayer containing 204 POPC molecules using CHARMM-GUI Membrane Builder. The systems were solvated with CHARMM-compatible TIP3P water, neutralized with sodium and chloride ions, and adjusted to an ionic concentration of 0.15 M. The CHARMM36m force field was applied to CXCR4, CHARMM36 parameters were used for POPC, and CGenFF parameters were used for the ligands. Energy minimization and the standard six-step CHARMM-GUI membrane-equilibration protocol were completed before production simulation.**

5. Simulating a transmembrane GPCR in a purely aqueous environment without explicit lipids (such as POPC or DPPC) is a major methodological flaw. Structural stability and realistic conformational dynamics of membrane proteins depends on the lipid environment. The authors should justify their decision not to use a membrane environment, or acknowledge this as a major limitation of the biological relevance of the 500 ns trajectory.

**Response: We agree with the reviewer’s assessment. The original water-only trajectory was not retained or used to support any conclusion in the revised manuscript. The complete simulation pipeline was repeated in an explicit POPC membrane environment, and all previous RMSD, RMSF, SASA, radius-of-gyration, PCA, FEL, DCCM, and MM/GBSA results were replaced.**

**The revised Discussion also acknowledges that a POPC-only membrane does not reproduce the complete lipid complexity of a mammalian plasma membrane. Future work using cholesterol-containing and more compositionally representative membranes is therefore identified as an important extension of the present simulations.**

**Statistical Significance & Reproducibility:**

6. The authors report results based on one 500 ns trajectory for luteolin and control complexes.

A single trajectory is not enough to remove stochastic bias in modern computational drug discovery. Reviewers typically want independent replicates (e.g. n=3) with different initial velocity seeds to confirm robustness of the observed stability, PCA clusters, and binding free energy.

**Response: We agree that a single trajectory cannot adequately assess reproducibility or distinguish persistent behaviour from stochastic trajectory-specific variation. The original single 500 ns simulations were removed.**

**In the revised study, the CXCR4 complexes with naringenin, apigenin, and IT1t were each simulated for 100 ns in two independent replicas initiated with different randomized velocity seeds. This produced six membrane-embedded production trajectories and an aggregate simulation time of 600 ns. All structural, conformational, interaction, and energetic analyses were conducted separately for Replica 1 and Replica 2, and replica-dependent differences are reported explicitly.**

**We acknowledge that two replicas remain below the reviewer’s example of three independent runs. Accordingly, the revised manuscript does not claim definitive statistical convergence or complete removal of stochastic uncertainty. The comparisons are interpreted descriptively, and the need for a minimum of three longer independently seeded simulations is stated as a future priority.**

**Discrepancies in Residue Indexing:**

7. The RMSF analysis (Figure 5B) indicates residue indices > 1000, whereas the protein-ligand interaction and DCCM maps show a residue range of about 300–360. This causes confusion about the sequence numbering. Could you please clarify if the high residue numbers in the RMSF plot correspond to certain chains, water molecules, or some other numbering used in the GROMACS analysis?

**Response: We thank the reviewer for identifying this inconsistency. The residue indices above 1000 in the original plots arose from the use of the engineered 3OE6 crystallographic construct containing the T4 lysozyme fusion and its associated residue numbering. They did not represent water molecules or additional native CXCR4 residues.**

**This issue was corrected by excluding the T4 lysozyme sequence and reconstructing a native human CXCR4 model containing intracellular loop 3. The revised model contains 306 CXCR4 residues, and all new RMSF, PCA, and DCCM calculations use the reconstructed receptor and its native residue range. The original plots with indices above 1000 were removed.**

**The high residue numbers in the original analysis corresponded to the non-native T4 lysozyme fusion region retained from the crystallographic construct. They did not correspond to water, ions, or a separate native CXCR4 chain. Because inclusion of this fusion partner was methodologically inappropriate, the complete receptor model and all associated analyses were replaced. The revised manuscript now reports only residues belonging to the reconstructed native CXCR4 receptor.**

**Minor revision notes**

RMSD Stability Interpretation:

1. The RMSD of the luteolin complex shows a substantial increase towards the end of the simulation, with values of 2.1 to 2.4 nm. Authors call this "adaptive flexibility" and "induced fit". But RMSD >2.0 nm (20 A) is very high for a stable complex. The authors should provide structural snapshots from the end of the simulation to show that the ligand remains in the binding pocket and that the protein has not partially unfolded or undergone significant structural distortion.

**Response: We agree that the previously reported RMSD values of approximately 2.1–2.4 nm were incompatible with the claim of a conventionally stable GPCR complex and should not have been described as “adaptive flexibility” or “induced fit.” These descriptions and the complete original luteolin trajectory were removed.**

**The revised membrane-embedded simulations produced substantially lower receptor-backbone RMSD ranges. For the prioritized CXCR4–naringenin system, the two replicas generally fluctuated within approximately 0.20–0.34 nm and approached similar values near 0.30 nm at the simulation endpoint. SASA and radius-of-gyration profiles remained within comparatively restricted ranges without sustained progressive expansion. Representative receptor–ligand structures were extracted from the final 100 ns frames for structural inspection. The revised interpretation is deliberately cautious and describes replica-dependent conformational sampling rather than claiming induced fit, complete convergence, or experimentally confirmed ligand retention.**

2. Differences in ADMET platforms:

There is a clear discrepancy between SwissADME and the ProTox 3.0 results in Cytochrome P450 inhibition (CYP1A2, CYP2C19, and CYP2C9). The authors should elaborate more on these contradictions. It would be helpful to rely on multiple consensus models or explain the variation in the training datasets for these tools, to add scientific rigor to the ADMET section.

**Response: We thank the reviewer for highlighting the limitations of directly comparing predictions from different platforms. The previous luteolin-focused comparison was removed because luteolin was no longer retained as the lead.**

**In the revised workflow, SwissADME was used for physicochemical, solubility, absorption, distribution, transporter, and rule-based drug-likeness predictions, whereas ProTox 3.0 was used separately for acute toxicity, organ-toxicity, toxicity-endpoint, nuclear-receptor, molecular-initiating-event, and metabolic alerts. The CYP outputs from ProTox are reported as model-based predictions and are not treated as experimentally confirmed enzyme inhibition. The revised Discussion emphasizes that predicted CYP interactions and other pharmacokinetic alerts require validation using microsomal stability and recombinant-enzyme assays. This separation avoids treating outputs generated from different algorithms, endpoint definitions, and training frameworks as directly interchangeable.**

3. Estrogen Receptor Activity:

For luteolin, ADMET predictions identified estrogen receptor alpha activity with relatively high confidence (0.76 to 0.78). This off-target prediction is important because endometriosis and ovarian cancer are both highly sensitive to hormonal signaling. Further discussion should take into consideration whether this activity is potentially synergistic (as a phytoestrogen) or a liability in the specific context of CXCR4-mediated cancer progression.

**Response: We agree that the predicted estrogen-receptor activity of luteolin was an important concern in the original disease context. Following correction of the docking workflow, luteolin was removed from lead prioritization and was not advanced to comparative ADME, toxicity, pharmacophore, DFT, molecular dynamics, or MM/GBSA analyses.**

**The revised toxicity assessment focuses on apigenin and naringenin and transparently reports their predicted estrogen receptor and estrogen receptor ligand-binding-domain activity in Table 6. These binary predictions are treated as potential off-target alerts and not as evidence of agonism, antagonism, therapeutic benefit, or clinical safety. Because the direction and biological consequences of estrogen-receptor modulation cannot be determined from these computational classifications, receptor-specific and estrogen-responsive functional assays are identified as necessary before either compound can be considered for disease-directed development.**

4. Formatting and References:

There are some internal inconsistencies in the manuscript, e.g., specific funding instructions and ethics statements are given which are then marked as "N/A"
. Delete all journal-specific instructions from the draft stages to enhance the professional appearance of the final manuscript.

**Response: Thank you for identifying these presentation issues. Journal-specific instructions, placeholder text, and declarations marked as “N/A” were removed from the manuscript. The required declarations are now provided in the relevant fields of the online submission system. Figures have been separated from the manuscript and prepared as individual files, while their legends and sequential in-text citations remain in the main document.**

**The title, headings, tables, figure legends, abbreviations, software citations, and reference list were also reviewed and revised for consistency. Claims in the Introduction and Discussion are now supported with relevant peer-reviewed literature, and the manuscript distinguishes published experimental evidence from the hypotheses generated by the present computational analyses.**

5. Conclusion of Review: The breadth of the work is technically well done, but the lack of a lipid bilayer in the simulation of a GPCR and lack of MD replicates are serious shortcomings that need to be addressed to validate the lead candidate.

**Response: We thank the reviewer for recognizing the breadth of the computational work and agree that the absence of a lipid bilayer and independent replicas were major weaknesses of the original submission. Both aspects were substantially revised.**

**The water-only simulations were replaced with explicit POPC membrane-embedded simulations, and two independently seeded 100 ns replicas were performed for each of the CXCR4 complexes with naringenin, apigenin, and IT1t. All previous luteolin-based trajectory analyses were removed and replaced with replica-specific structural, conformational, interaction, and energetic analyses.**

**We acknowledge that two replicas do not fully meet the commonly recommended design of three or more independent trajectories. The revised manuscript therefore avoids definitive claims of convergence and explicitly presents this as a limitation. Naringenin is described only as an in silico prioritized CXCR4-binding candidate requiring receptor-binding, functional, pharmacokinetic, toxicological, and disease-model validation.**
