# Supplementary material for: Computational identification of natural CXCR4-binding candidates relevant to endometriosis and ovarian cancer progression
Source: PLoS One. 2026 Oct 7;21(10):e0359833. doi: 10.1371/journal.pone.0359833 (PMC13645088; doi:10.1371/journal.pone.0359833)
Supplement: Supplementary file 4 [file pone.0359833.s006.docx]

**Point by point response**

**We sincerely thank the editor and the reviewer for the careful evaluation of our manuscript and for the constructive comments provided during this round of review. The suggestions have helped us to improve the clarity, presentation, and methodological transparency of the work. We have addressed each comment individually, and all corresponding revisions have been incorporated into the manuscript and highlighted for ease of identification. Our point-by-point responses are provided below.**

**Reviewer #3:**

1. Change the 3D structure to 2D structure in table 1.

**Response:** **We thank the reviewer for this helpful suggestion. All compound depictions in Table 1 have been replaced with two-dimensional chemical structures. The revised table now presents each phytochemical, and the crystallographic reference ligand, as a standard 2D structural diagram, which makes the substitution patterns and functional groups directly readable and is consistent with the presentation style used in comparable computational reports. The revised Table 1 is highlighted in the resubmitted manuscript.**

2. End of the conclusion give the list of abbreviation used in this manuscript

**Response:** **We agree, and a dedicated "Abbreviations" section has been added immediately after the Conclusion and before the Supporting information section. The section lists 47 abbreviations used throughout the manuscript, from ADME to VEGFR, with one abbreviation per line in the format "ABBR: expansion" in accordance with PLOS ONE formatting requirements. The list was compiled by screening the complete manuscript text, so that every abbreviation appearing in the abstract, main text, tables, and figure captions is defined. The new section is highlighted in the revised manuscript.**

3. Give proper subscript in the Chemical molecular formula

**Response:** **We thank the reviewer for noting this formatting error. The molecular formulas of apigenin and naringenin in the "Formula" row of Table 5 have been reformatted so that all numerical indices are now true subscripts, and they read C_15_H_10_O_5_ and C_15_H_12_O_5_ with subscripted numerals in the revised file. We also re-checked the entire manuscript, including the abstract, main text, tables, figure captions, and supporting information, and confirmed that these were the only chemical molecular formulas presented; no other formula therefore required correction. The corrected cells are highlighted.**

4. Make sure free from grammatical mistakes

**Response:** **The complete manuscript has been re-read and language-edited paragraph by paragraph. During this pass we corrected a redundant space preceding a full stop in the Introduction, two instances of double spacing in the Methods and Results text, and a bold formatting run that incorrectly extended beyond the in-text figure citation into the following sentence. Sentence construction, subject-verb agreement, article usage, hyphenation of compound modifiers, and consistency of technical terminology were also checked throughout, and en-US spelling has been applied uniformly. All newly written passages were drafted in the same academic register as the existing text.**

5. Give high-quality pictures

**Response:** **All figures have been regenerated and replaced with high-resolution versions prepared in accordance with the PLOS ONE figure guidelines. The replacement images were exported at publication resolution so that receptor renderings, interaction diagrams, and simulation plots remain legible at print size, and axis labels, residue labels, legends, and panel letters are now clearly readable without magnification. The figure files are supplied separately in the required format with the resubmission.**

6. In what based you selected the phytochemicals

**Response:** **We appreciate this important question and have clarified the selection basis explicitly in the Methods. A new paragraph has been added to the subsection "Selection and retrieval of phytochemical structures" describing the predefined criteria that were applied before any structural or docking analysis was performed. A compound was eligible when peer-reviewed in vitro or in vivo evidence described an effect on at least one disease-relevant biological process in endometriosis, ovarian cancer, or a mechanistically related gynecological or oncological model; when it originated from a dietary or medicinal plant source and was therefore obtainable for subsequent experimental follow-up; when an unambiguous PubChem Compound Identifier and a complete three-dimensional structure were available for retrieval; and when its physicochemical properties fell within the chemical space typically associated with orally administered small molecules, so that the downstream pharmacokinetic and toxicity predictions would remain interpretable. The eligible compounds were then grouped by chemical class, and representatives of flavones, flavanones, flavonols, stilbenes, phenolic acids, terpenoids, and alkaloids were retained so that the final panel of 18 compounds sampled structurally distinct scaffolds rather than close analogues of a single class. Compounds were excluded when the supporting evidence originated from unrelated disease contexts, when the reported activity could not be traced to a primary experimental study, when the deposited structure was incomplete or stereochemically ambiguous, or when the compound belonged to a chemical class associated with non-specific assay interference. Because the panel was defined solely by literature-based and structural criteria, and because none of the selected compounds had an established CXCR4-directed mechanism of action, the screen was designed to be hypothesis-generating rather than confirmatory. The added paragraph is highlighted in the revised manuscript.**
